# Pacific Biosciences assembly with Hi-C mapping generates an improved, chromosome-level goose genome

**DOI:** 10.1093/gigascience/giaa114

**Published:** 2020-10-24

**Authors:** Yan Li, Guangliang Gao, Yu Lin, Silu Hu, Yi Luo, Guosong Wang, Long Jin, Qigui Wang, Jiwen Wang, Qianzi Tang, Mingzhou Li

**Affiliations:** Institute of Animal Genetics and Breeding, College of Animal Science and Technology, Sichuan Agricultural University, No.211 Huimin Road, Wenjiang District, Chengdu 611130, China; Institute of Animal Genetics and Breeding, College of Animal Science and Technology, Sichuan Agricultural University, No.211 Huimin Road, Wenjiang District, Chengdu 611130, China; Institute of Poultry Science, Chongqing Academy of Animal Science, No. 51 Changlong Avenue, Rongchang District, Chongqing 402460, China; Institute of Animal Genetics and Breeding, College of Animal Science and Technology, Sichuan Agricultural University, No.211 Huimin Road, Wenjiang District, Chengdu 611130, China; Institute of Animal Genetics and Breeding, College of Animal Science and Technology, Sichuan Agricultural University, No.211 Huimin Road, Wenjiang District, Chengdu 611130, China; Institute of Animal Genetics and Breeding, College of Animal Science and Technology, Sichuan Agricultural University, No.211 Huimin Road, Wenjiang District, Chengdu 611130, China; Institute of Animal Genetics and Breeding, College of Animal Science and Technology, Sichuan Agricultural University, No.211 Huimin Road, Wenjiang District, Chengdu 611130, China; Department of Animal Science, Texas A&M University, 2471 TAMU, College Station, Texas 77843, USA; Institute of Animal Genetics and Breeding, College of Animal Science and Technology, Sichuan Agricultural University, No.211 Huimin Road, Wenjiang District, Chengdu 611130, China; Institute of Poultry Science, Chongqing Academy of Animal Science, No. 51 Changlong Avenue, Rongchang District, Chongqing 402460, China; Institute of Animal Genetics and Breeding, College of Animal Science and Technology, Sichuan Agricultural University, No.211 Huimin Road, Wenjiang District, Chengdu 611130, China; Institute of Animal Genetics and Breeding, College of Animal Science and Technology, Sichuan Agricultural University, No.211 Huimin Road, Wenjiang District, Chengdu 611130, China; Institute of Animal Genetics and Breeding, College of Animal Science and Technology, Sichuan Agricultural University, No.211 Huimin Road, Wenjiang District, Chengdu 611130, China

**Keywords:** goose genome, chromosome-length assembly, hybrid *de novo* assembly approaches, annotation, PacBio, Hi-C

## Abstract

**Background:**

The domestic goose is an economically important and scientifically valuable waterfowl; however, a lack of high-quality genomic data has hindered research concerning its genome, genetics, and breeding. As domestic geese breeds derive from both the swan goose (*Anser cygnoides*) and the graylag goose (*Anser anser*), we selected a female Tianfu goose for genome sequencing. We generated a chromosome-level goose genome assembly by adopting a hybrid *de novo* assembly approach that combined Pacific Biosciences single-molecule real-time sequencing, high-throughput chromatin conformation capture mapping, and Illumina short-read sequencing.

**Findings:**

We generated a 1.11-Gb goose genome with contig and scaffold N50 values of 1.85 and 33.12 Mb, respectively. The assembly contains 39 pseudo-chromosomes (2n = 78) accounting for ∼88.36% of the goose genome. Compared with previous goose assemblies, our assembly has more continuity, completeness, and accuracy; the annotation of core eukaryotic genes and universal single-copy orthologs has also been improved. We have identified 17,568 protein-coding genes and a repeat content of 8.67% (96.57 Mb) in this genome assembly. We also explored the spatial organization of chromatin and gene expression in the goose liver tissues, in terms of inter-pseudo-chromosomal interaction patterns, compartments, topologically associating domains, and promoter-enhancer interactions.

**Conclusions:**

We present the first chromosome-level assembly of the goose genome. This will be a valuable resource for future genetic and genomic studies on geese.

## Data Description

### Context

The goose is a member of the family Anatidae and is an economically important waterfowl with distinctive characters. Domesticated geese derive from the swan goose (*Anser cygnoides*) and the graylag goose (*Anser anser*) [1], and ∼6,000 years of artificial selection have led to significant alterations in their body size, reproductive performance, egg production, feather color, and other features [2]. Currently, >181 domesticated breeds are reared globally to supply meat, eggs, and valuable by-products (feathers, fatty liver) for human consumption [2–4]. The domestic goose is also well suited to sustainable production practices because fiber can form part of its diet, which then lessens competition for human food [5]. Its excellent disease resistance and behavioral patterns also allow for large-scale farming and easy management [6]. Interestingly, despite the liver weight of goose increasing 5–10 times after 2–3 weeks of overfeeding, the amount of fat in hepatic cells (and other biomedical parameters) returns to normal levels when overfeeding ceases. This suggests that the goose liver could provide a novel animal model for the study of human non-alcoholic fatty liver disease [6].

The goose was one of the earliest animals to be domesticated [2, 7], and wide-ranging genomic and breeding research has been conducted to study its domestication process and the unique morphological and physiological features of these animals. For example, recently published goose genome sequences have been assembled into scaffolds using short reads from the Illumina platform [8, 9]; however, the genetic basis of the fatty liver of goose and their selective breeding remains largely unknown. To address such issues, a high-quality genome sequence is required. Currently, there are many advantages to using hybrid *de novo* assembly approaches to improve the quality of genome assemblies. This is because short, accurate reads from the Illumina platform can be combined with the longer, less accurate reads generated by the single-molecule real-time (SMRT) sequencing platform [10]. With Hi-C, linking information can then be ordered and oriented into scaffolds, after which assembly errors can be identified and corrected [11]. This approach has been applied to improve the genome assemblies of many species, including humans [12], goats [13], rockfish [14], *Aedes aegypti* [11], and barley [15].

Here, we have generated a chromosome-level goose assembly with chromosome-length scaffolds by adopting a hybrid *de novo* assembly approach using a combination of short reads from the Illumina platform, long reads from the Pacific Biosciences (PacBio) platform, and Hi-C–based chromatin interaction maps. Our chromosome-level goose genome comprises longer scaffolds than currently available goose genome assemblies, and these scaffolds are of a higher quality and are more continuous and accurate. Our new genome assembly thus provides a valuable resource for exploring the molecular basis of the morphological and physiological features of the goose and will facilitate further genomic, genetic, and breeding studies of this domesticated waterfowl.

### Methods

#### Sample collection and sequencing

**Figure 1:**
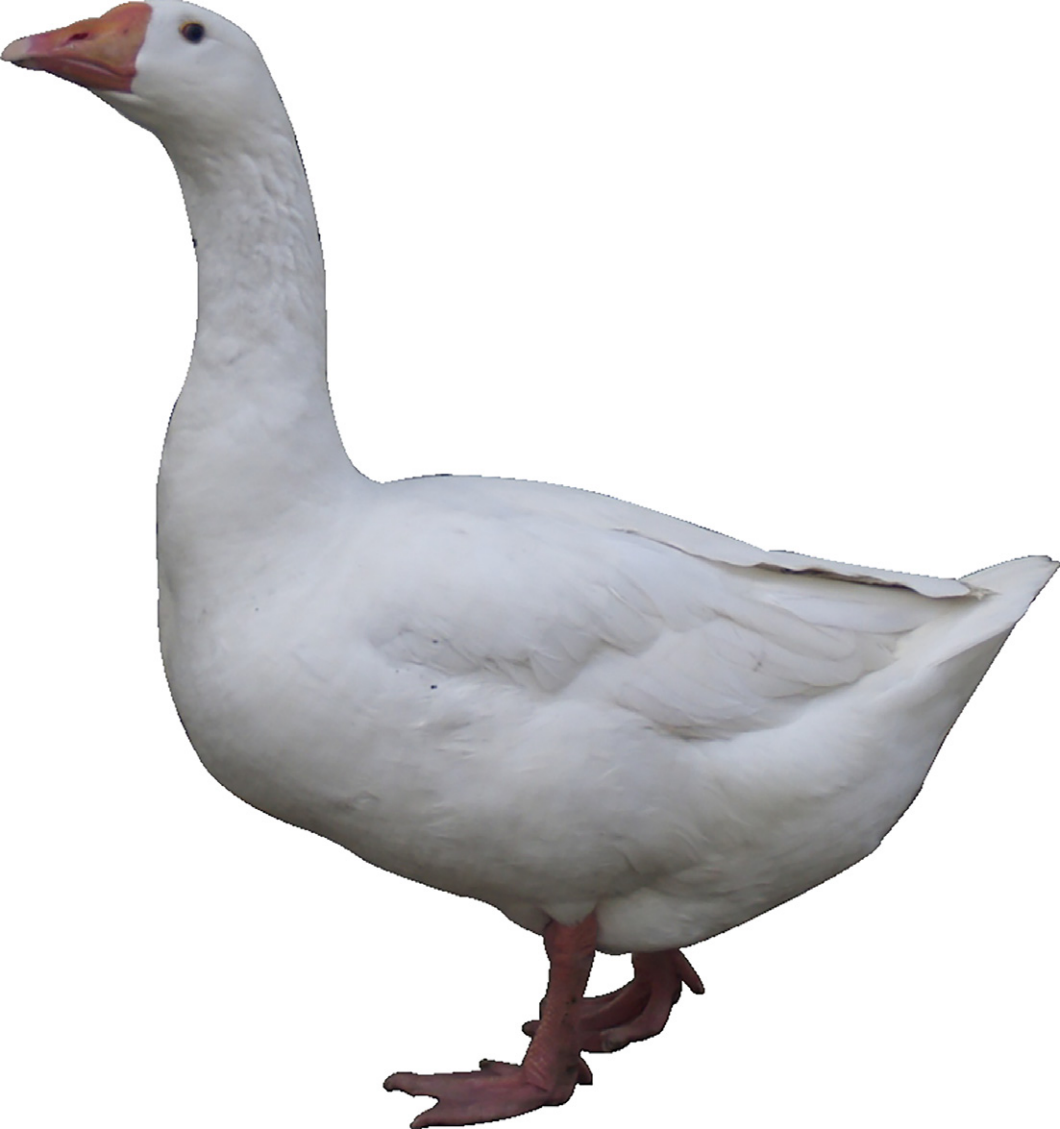
A picture of a female adult goose used for genome sequencing.

We extracted genomic DNA from the liver tissue of a healthy adult female (136 days old) from the Tianfu goose maternal line (NCBI:txid381198), which was provided by the Experimental Farm of Waterfowl Breeding of Sichuan Agricultural University (Chengdu, Sichuan, China; Fig. 1). We then carried out SMRT DNA sequencing of ∼20-kb inserts using the PacBio Sequel platform. This yielded ∼84.31 Gb of high-quality sequencing data that were used to initially assemble the genome (Table 1). Next, 149.70 Gb of high-quality sequencing data were generated from a 350-bp insert size Hi-C library, as previously reported [13]. Finally, 350-bp paired-end libraries constructed from the same genomic DNA were sequenced on the Illumina HiSeq platform, producing a further 181.52 Gb of sequence data. In total, we obtained ∼415.53 Gb sequencing data (∼324.63× coverage) for our chromosome-level goose genome assembly (Table 1).

**Table 1:** Summary of sequencing data for goose genome assembly

Paired-end libraries	Insert size (bp)	Total data (Gb)	Read length (bp)	Sequence coverage ($\times $)
Illumina reads	350	181.52	150	141.81
PacBio reads	20,000	84.31	7,732	65.86
Hi-C	350	149.70	150	116.95
Total		415.53		324.63

#### *De novo* assembly of the goose genome

The size of the goose genome was estimated by *k*-mer distribution analysis to be 1.28 Gb. To assemble the genome, we first performed an initial assembly with the PacBio long reads alone, using Falcon (Falcon, RRID:SCR_016089) [16] software. We used the pbsmrtpipe pipeline of the smrtlink (smrtlink, RRID:SCR_002942) software to assemble the genome sequence, which resulted in a draft assembly with a contig N50 of 1.72 Mb (Supplementary Table S1). Next, we used the single-molecule sequence reads to scaffold these contigs and fill gaps, using SSPACE-Long (SSPACE-Long, RRID:SCR_005056) [17] and PBJelly (PBJelly, RRID:SCR_012091) [18], respectively. Pilon (Pilon, RRID:SCR_014731) [19] software was then used to map the short reads to the assembly (Supplementary Table S1). Finally, 39 pseudo-chromosomes were assembled with the Hi-C reads aligned using Lachesis (Lachesis, RRID:SCR_017644) [20] software (Supplementary Table S2, Supplementary Fig. S1); this is consistent with the number of goose chromosomes (2n = 78) reported in previous studies [21]. With these methods, we generated a chromosome-level goose assembly with a contig N50 of 1.85 Mb and scaffold N50 of 33.12 Mb (Table 2). The average GC content is 42.15% and the total genome size is 1.11 Gb, which is consistent with previous studies [8, 9] and suggests that our goose assembly is reliable.

**Table 2:** Comparison of quality metrics of this study and the previous goose genome assemblies

Genomic features	This study	Lu et al. [8]	Gao et al. [9]
Estimate of genome size (bp)	1,277,099,016	1,208,661,181	1,198,802,839
Total length of assembled contigs (bp)	1,113,842,245	1,086,838,604	1,100,859,441
Total size of assembled scaffolds (bp)	1,113,913,845	1,122,178,121	1,130,663,797
Number of contigs (>2 kb)	2,771	60,979	53,336
Number of scaffolds (>2 kb)	2,055	1,050	1,837
Contig N50 (bp)	1,849,874	27,602	35,032
Scaffold N50 (bp)	33,116,532	5,202,740	5,103,766
Longest contig (bp)	10,766,871	201,281	399,111
Longest scaffold (bp)	70,896,740	24,051,356	20,207,557
GC content	42.15%	38.00%	41.68%
No. of genes model	17,568	16,150	16,288
Repetitive regions proportion of genome	8.67%	6.33%	6.90%

#### Repeat sequence and gene annotation

*De novo* methods and homology-based approaches were used to annotate the repeat content of the goose genome. First, we used *ab initio* prediction software, including LTR-finder (LTR-finder, RRID:SCR_005659) [22], RepeatMolder (RepeatMolder, RRID:SCR_015027) [23], and RepeatScout (RepeatScout, RRID:SCR_014653) [24], to perform *de novo* annotation of the genome. For homology-based predictions, we identified repeat regions across species in published RepBase sequences [25] using RepeatMasker (RepeatMasker, RRID:SCR_012954) [26] and RepeatProteinMask (RepeatProteinMask, RRID:SCR_012954) [27] software. Combined with these results, the repeat region of the goose genome was further predicted with RepeatMasker software. From these analyses, we identified 92.11 Mb of repetitive DNA (Supplementary Table S3) accounting for 8.67% of our assembly, which is much higher than has been reported in previous studies [8, 9]. Long interspersed nuclear elements (LINEs) were the most abundant repeat element identified, representing 6.83% of the genome. The proportion of LINE repetitive sequences identified in this study was also higher than has been reported in 2 previous goose genome assemblies (Supplementary Table S3). We performed protein-coding gene (PCG) annotation by combining *ab initio*–based, homology-based, and RNA sequencing (RNA-seq)-based prediction methods. First, GenScan (GenScan, RRID:SCR_012902) [28], Geneid (Geneid, RRID:SCR_002473) [29], and Augustus (Augustus, RRID:SCR_008417) [30] were used for *ab initio*–based predictions. Next, we selected 6 chromosome-level genomes, namely, *Homo sapiens* (GCF_000001405.39), *Mus musculus* (GCF_000001635.26), *Gallus gallus* (GCF_000002315.6), *Anas platyrhynchos* (GCF_003850225.1), *Meleagris gallopavo* (GCF_000146605.3), and *Taeniopygia guttata* (GCF_003957565.1), to use for homology-based annotation of our goose chromosome-level assembly genome using TBLASTN (TBLASTN, RRID:SCR_011822) [31] and GeneWise (GeneWise, RRID:SCR_015054) [32] software. We found 8,255 common orthologous groups across these 7 species (Supplementary Fig. S2). To optimize genome annotation, total RNA was extracted from 11 samples (abdominal fat, brain, duodenum, heart, liver, lung, muscular stomach, ovary, pancreas, pectoral muscle, and spleen) taken from the same individual whose DNA was used for the chromosome-level genome assembly. We pooled equal amounts of the total RNA from each of the 11 tissues and then performed RNA-seq on this pooled sample using the Illumina platform. After filtering, these data were used to annotate protein-coding regions of the genome assembly using Trinity (Trinity, RRID:SCR_013048) [33] and TopHat (TopHat, RRID:SCR_013035) [34]. Finally, the predictions from each method described above were integrated using EVM (EVM, RRID:SCR_014659) [35]; overall, 17,568 PCGs were predicted (Table 3, Supplementary Fig. S3). To identify long noncoding RNAs (lncRNAs), the goose genome reads were aligned by STAR (STAR, RRID:SCR_015899) [36] and subjected to Cufflinks (Cufflinks, RRID:SCR_014597) [37] and TACO [38] for assembly and filtering. CPC2 (CPC2, RRID:SCR_002764) [39] was then applied to perform coding potential analysis, and PfamScan (PfamScan, RRID:SCR_004726) [40] was used to check for domain hits against Pfam31-A [41]. After removal of all likely domains, 3,287 lncRNAs only by *ab initio* assembly method and 542 transcripts of uncertain coding potential (TUCP) were identified; the long reads will be helpful to improve the identification and annotation of the lncRNA and TUCP in goose genome.

**Table 3:** Summary of predicted genes within each goose genome assembly

Property	This study	Lu et al. [8]	Gao et al. [9]
Total PCG length (bp)	326,863,440	439,289,059	500,923,091
No. of PCGs	17,568	16,150	16,288
PCG proportion of genome	29.34%	39.25%	44.31%
No. of total exons	152,392	158,713	167,532
Mean exons per gene	8.67	10.92	10.29
Total exon length (bp)	26,883,354	25,763,242	26,157,477
Exons proportion of genome	2.41%	2.31%	2.31%
Mean exon length (bp)	176.41	162.33	156.13
Mean intron length (bp)	2,224.97	2,867.48	3,139.07

### Data validation and quality control

#### Assessment of genome assembly completeness

**Figure 2:**
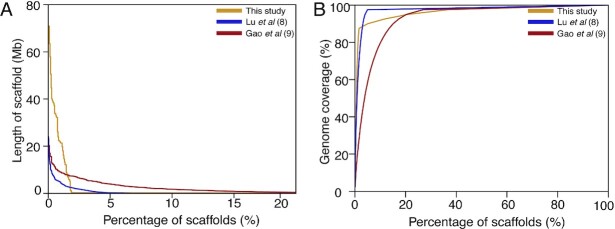
Comparison of the distribution and coverage of the scaffolds for the assembly with previous goose genome assemblies.

Our assembly has more scaffolds and fewer contigs, and significantly improved contig and scaffold N50 values, than the goose genome assemblies presented in 2 previous studies (Fig. 2). Moreover, we have annotated more repeat (Supplementary Table S3) and exon sequence regions (Table 3) than these previous studies (Table 3), which suggests that we have generated an improved genome assembly and annotation. The 39 pseudo-chromosomes described in our study account for 88.36% of the assembled genome and are longer than those previously reported [8, 9], again indicating that our chromosome-level goose genome represents a significant improvement on previous work. The GC content of our genome assembly is 42.15% and the size of the genome is 1.11 Gb (Table 2). This is comparable to the sizes reported for the 2 previously constructed goose genomes [8, 9] and is characteristic of avian genomes [42]. We also mapped short-insert paired-end reads (350 bp) to our chromosome-level goose genome and obtained mapping and coverage rates of 97.25% and 99.71%, respectively. Finally, we downloaded 19 wild goose resequencing [43] datasets from public databases and mapped them to our assembly and to the 2 earlier draft goose genomes. We found that the mapping rate of our chromosome-level goose assembly was higher than that of the previously assembled genomes (Supplementary Table S4), indicating that it is more contiguous. Taken together, these results demonstrate the improvements made by our study in the assembly and annotation of the goose genome, in comparison with previous studies [8, 9].

To evaluate the completeness of our chromosome-level genome assembly, we determined the number of conserved eukaryotic and universal genes present in our assembly by applying the CEGMA software (CEGMA, RRID:SCR_015055) and using a set of BUSCO (BUSCO, RRID:SCR_015008). We found that 211 of the 248 (85.08%) core eukaryotic genes and 2,586 (97%) of the universal single-copy orthologs were assembled in our genome. Compared with previous studies, this suggests that our genome assembly is more complete than previous drafts of the goose genome [8, 9].

To explore the hypothesis that the leptin gene was lost from goose [8], we downloaded leptin sequences from avian and mammal genomes to use as reference sequences in BLASTP (BLASTP, RRID:SCR_001010) searches of our newly assembled goose genome. We found no sequences similar to leptin in our chromosome-level goose assembly. Furthermore, although the human genome region that contains the leptin gene (chromosome 7, 126.0–129.4 Mb) aligned with the goose genome, we did not find a sequence similar to the leptin gene in this region. These results confirm the previous finding that the leptin gene is not present in the goose genome [8].

#### Phylogenetic tree and lineage-specific gene families

Using OrthoMCL (OrthoMCL, RRID:SCR_007839) [44], 16,157 orthologous gene families across 17 species (ostrich, duck, goose, chicken, turkey, saker, red-legged seriema, African crowned crane, pelican, little egret, crested ibis, cormorant, great crested grebe, pigeon, woodpecker, zebra finch, and lizard) were identified. On the basis of 2,389 shared single-copy ortholog gene clusters, we constructed a maximum likelihood phylogenetic tree using the RAxML software (RAxML, RRID:SCR_006086) [45]. This revealed that goose and duck diverged ∼31.60 million years ago, which is comparable to the divergence time of chicken and turkey (32.33 million years ago; Supplementary Fig. S4) and consistent with the previous studies [8, 9]. We also noted that lineage-specific genes in the goose genome were significantly enriched for olfactory receptor activity (GO:0 004984, *P* = 3.85 × 10^−24^), G protein-coupled receptor activity (GO:0 004930, *P* = 6.67 × 10^−13^), and integral component of membrane (GO:00 16021, *P* = 0.01; Supplementary Table S5). As migratory birds, geese are adapted for long-distance migration, which exposes them to a diversity of food as they seek out ideal habitats. We propose that such influences might strengthen the interactions between odorants and the receptors of the olfactory mucosa, and could underlie receptor family evolution in the goose genome.

#### Expansion and contraction of gene families

The expansions and contractions of gene clusters in the goose genome were identified in comparison with 9 other avian genomes using the CAFE program (CAFÉ, RRID:SCR_018924) [46]. We found 839 expanded gene families (Supplementary Table S6) and 2,193 contracted gene families (Supplementary Table S7). Interestingly, the expanded gene families were mainly enriched for olfactory receptor activity (GO:0 004984, *P* = 8.58 × 10^−51^), G protein-coupled receptor activity (GO:0 004930, *P* = 5.81 × 10^−25^), and integral component of membrane (GO:00 16021, *P* = 3.20 × 10^−6^), which is consistent with the results from our analysis of lineage-specific genes (Supplementary Table S5). This further confirms that the migratory adaptations of the goose are reflected by unique characteristics in the goose genome that contrast with those of nonmigratory birds. Other expanded gene families were enriched for ATPase-coupled transmembrane transporter activity (GO:00 42626, *P* = 1.96 × 10^−06^), NAD(P)+-protein-arginine ADP-ribosyl transferase activity (GO:0 003956, *P* = 3.20 × 10^−04^), ATPase activity (GO:00 16887, *P* = 8.28 × 10^−05^), and aspartic-type endopeptidase activity (GO:0 004190, *P* = 9.63 × 10^−06^; Supplementary Table S6), while gene families contracted in the goose were significantly enriched for transmembrane transport (GO:00 55085, *P* = 8.30 × 10^−04^), ion channel activity (GO:0 005216, *P* = 1.87 × 10^−9^), ion transmembrane transport (GO:00 34220, *P* = 5.30 × 10^−6^), and ATPase-coupled intramembrane lipid transporter activity (GO:014 0326, *P* = 8.60 × 10^−10^; Supplementary Table S7). Because these pathways are related to ATP utilization, ATP production, and energy regulation, these data support a previous finding that goose energy metabolism is different from that in other avian species [47]. This feature of the goose is possibility related to its migratory habits and artificial selection—the goose is unique among migratory birds because of its large body size, which requires much energy for long-distance, high-altitude flying [48].

#### Genes under positive selection

We identified 52 positively selected genes (PSGs) in the goose genome based on orthologous genes from the 17 aforementioned species, using a branch-site model and F3 × 4 codon frequencies in Codeml (Codeml, RRID:SCR_004542) (Supplementary Table S8). Some of these PSGs, such as *GCH1* (GTP-cyclohydrolase I), are associated with Parkinsonism, dystonia, and phenylketonuria disease in humans [49, 50]. They also play a role in adaptation to high-altitude environments in humans, where they relate to a lower hemoglobin level, nitric oxide concentration, and oxygen saturation in the blood. Furthermore, previous studies have shown *GCH1* divergence between human populations living at different altitudes [51]. Selection acting on *GCH1* in goose is likely to be related to their adaption to high-altitude or migratory habitats. *SNW1* (SNW1 domain containing 1) is involved in the nuclear factor κB pathway and is associated with oculopharyngeal muscular dystrophy disease [52, 53]. The depletion of this gene in breast cells leads to the induction of apoptosis, while the overexpression of this gene impedes neural crest development [54]. Selection acting on *SNW1* in goose suggests that it may confer protection from diseases and aid adaptation in changeable environments. *POU2F3* (POU domain class 2 transcription factor 3) is pivotal in the discrimination of taste qualities, such as sweet, umami, and bitter characteristics. Deficiency in this gene in mice alters their electrophysiology and behavioral responses to taste characters [55, 56]. Selection acting on *POU2F3* in goose is likely to be related to a requirement for seeking food in variable migratory habitats.

#### Initial characterization of the 3D organization of goose liver tissues

**Figure 3:**
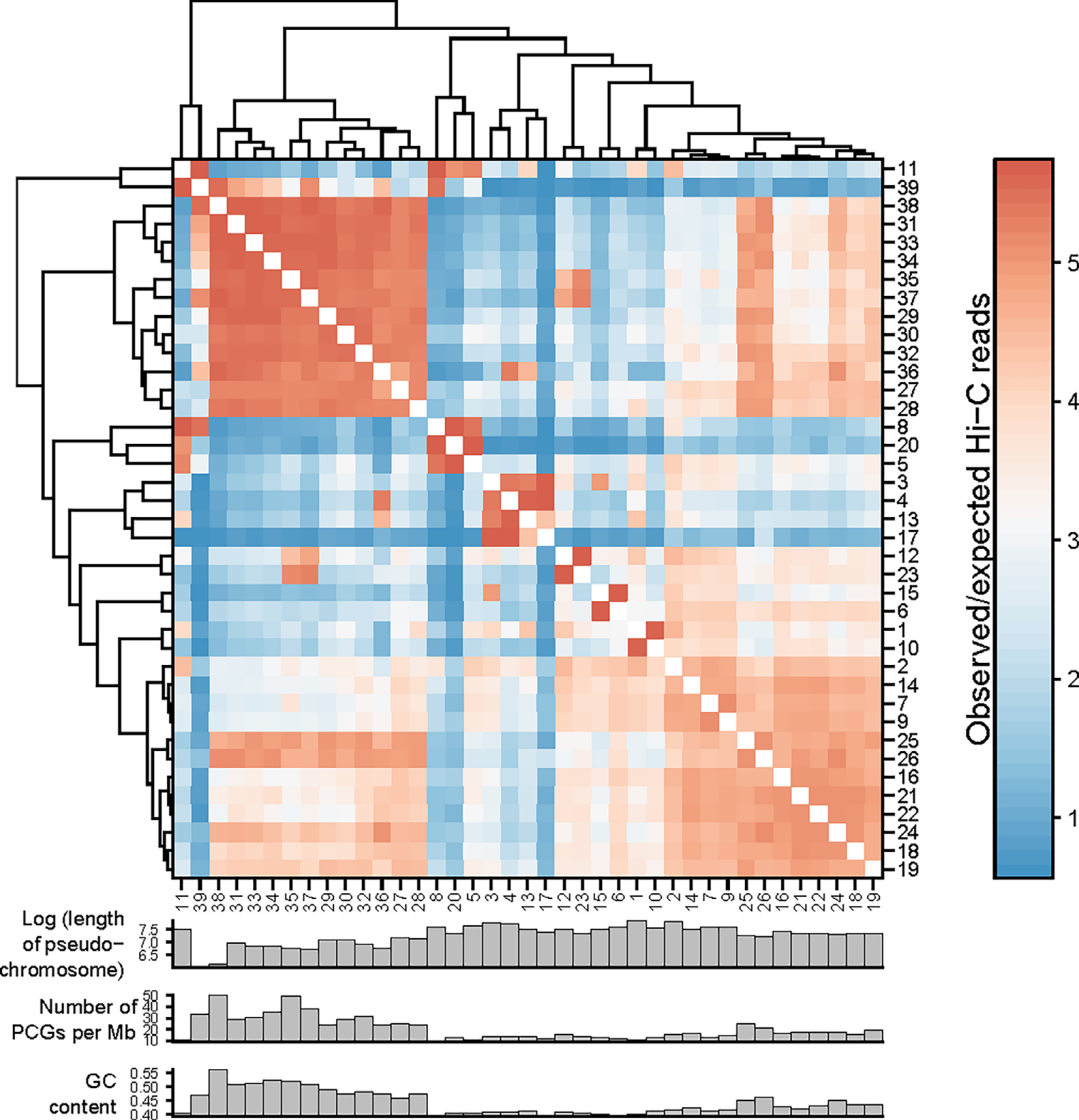
Dendrogram of inter-pseudo-chromosome interaction patterns generated by the average linkage algorithm.

We analyzed the inter-pseudo-chromosomal interaction pattern [57], compartments [58, 59], topologically associating domains (TADs) [60], and promoter-enhancer interactions (PEIs) [61] of the goose liver tissue. The matrix resolution of our Hi-C experiment reached ∼2 kb (defined as the smallest locus size such that 80% of loci have ≥1,000 contacts) (Supplementary Fig. S5), which was adequate for subsequent analyses of the chromatin architecture. Our results showed that the whole inter-pseudo-chromosomal interaction pattern was distinguished by 2 clusters, i.e., short pseudo-chromosomes and longer pseudo-chromosomes, which suggests that goose pseudo-chromosomes tend to interact with one another on the basis of size (Fig. 3). As for the identification of A and B compartments, which represent relatively active and inactive chromatin states, respectively, the number of PCGs in each 100-kb bin with ≥50% percentage overlapped with a gene was counted. The number of PCGs was significantly correlated with PC1 values (*R* = 0.39, *P* = 2.2 × 10^−16^; Supplementary Fig. S6), and the transcripts per kilobase millions (TPMs) of PCGs located in A compartments were consistently higher than PCGs in B compartments in 3 liver tissues (*P* = 2.2 × 10^−16^; Supplementary Fig. S7 and Table S9). We identified 734 TADs across the goose assembly, accounting for 80% of the genome (Supplementary Fig. S8 and Table S10). The mean and median sizes of the TADs were 1.21 and 1.00 Mb, respectively. We also observed that the transcription start sites of PCGs were enriched in TAD-boundary regions (Supplementary Fig. S9). After filtering for interaction distances <20 kb, we identified 13,017 PEIs (Supplementary Table S11) and found that gene expression levels positively correlated with the number of its associated enhancers in all 3 liver tissues (Supplementary Fig. S10). This is suggestive of additive effects of enhancers on target-gene transcription levels.

## Availability of Supporting Data and Materials

The chromosome-level goose genome assembly sequence is available at NCBI GenBank through accession No. WTSS00000000; the high-quality Hi-C data are available through the NCBI SRA database under accession No. SRR10483522. The PacBio long-read sequencing data have been deposited in the NCBI SRA (SRR10483521). The high-quality Illumina short-read sequencing data are available through NCBI SRA accession Nos. SRR10483516, SRR10483517, SRR10483518, and SRR10483520. The transcriptome data are available through NCBI SRR10483519. The chromosome-level goose genome assembly, annotation files, and other supporting data are available via the *GigaScience* GigaDB database [62].

## Additional Files

Supplementary Figure S1. The Hi-C interaction contact heatmap of goose pseudochromosome genome assembly (bin size is 1Mb).

Supplementary Figure S2. The shared homologous gene families across the six species (chicken, goose, human, mouse, pig, zebra finch).

Supplementary Figure S3. The distribution of gene density in the goose genome. Number of PCGs in each 1Mb bins was counted.

Supplementary Figure S4. Divergence of time and the expansion, contraction gene families in the seventeen species.

Supplementary Figure S5. Resolution evaluation showing that the Hi-C data attained 2 Kb.

Supplementary Figure S6. Vioplot of PC1 values in 100 Kb bins with various number of PCGs. PC1 value indicates the chromatin activity.

Supplementary Figure S7. TPMs of PCGs located in A compartment were consistently higher than PCGs in B compartment both at 25 Kb and 100 Kb resolution.

Supplementary Figure S8. TAD distribution across the goose genome assembly.

Supplementary Figure S9. TSSs of PCGs were enriched in TAD boundary regions.

Supplementary Figure S10. Gene expression levels positively correlated with the number of its associated enhancers in all three liver tissues.

Supplementary Table S1. Summary of the PacBio initial assembly and Hi-C read mapping used for goose genome assembly process.

Supplementary Table S2. Summary of the length of pseudo-chromosomes in goose genome.

Supplementary Table S3. A comparative summary of assembled repeat content in this study and previous studies.

Supplementary Table S4. Summary of the map rates of the wild goose resequencing data.

Supplementary Table S5. Gene ontology (GO) enrichment analysis for the lineage-specific gene annotation in goose genome.

Supplementary Table S6. Functional gene categories enriched for the goose genome–specific expansion gene families.

Supplementary Table S7. Functional gene categories enriched for the contraction of gene families in goose genome.

Supplementary Table S8. Positively selected genes (PSGs) identified in the goose genome.

Supplementary Table S9. The PC1 values (100 kb) through principal component analysis (PCA) and A-B index values (25 kb).

Supplementary Table S10. TAD in genome coordinates of our goose genome by using method of Directionality Index values.

Supplementary Table S11. Detailed information on promoter-enhancer interactions (PEIs) identified in goose genome.

## Abbreviations

ATP: adenosine triphosphate; BLAST: Basic Local Alignment Search Tool; bp: base pairs; BUSCO: Benchmarking Universal Single-Copy Orthologs; CHMP1B: charged multivesicular body protein 1B; CEGMA: Core Eukaryotic Genes Mapping Approach; EVM: EVidenceModeler; Gb: gigabase pairs; GC: guanine-cytosine; GCH1: GTP cyclohydrolase 1; Hi-C: chromosome conformation capture; kb: kilobase pairs; LINE: long interspersed nuclear element; lncRNA: long noncoding RNA; Mb: megabase pairs; NCBI: National Center for Biotechnology Information; PacBio: Pacific Biosciences; PCG: protein-coding gene; PEI: promoter-enhancer interaction; POU2F3: POU domain class 2 transcription factor 3; PSG: positively selected gene; RAxML: Randomized Axelerated Maximum Likelihood; RNA-seq: RNA sequencing; SMRT: single-molecule real-time; SRA: Sequence Read Archive; TAD: topological associated domain; TPM: transcripts per kilobase million; TUCP: transcripts of uncertain coding potential.

## Ethics Approval

All animal experiments were approved and reviewed by the Animal Care and Use Committee Institutional of Sichuan Agricultural University (Approval No. DKY-B20121406) and the Ministry of Science and Technology of the People's Republic of China (Approval No. 2006–398).

## Competing Interests

The authors declare that they have no competing interests.

## Authors' Contributions

M.L. and G.G. designed and supervised the project. Y. Li, Y. Lin, Q.T., and S.H. performed bioinformatics analyses. J.W., Y. Li, G.Wang, and Y. Luo contributed to collecting the samples. M.L., Q.W., G.G., Y. Luo, G.Wang, and L.J. were involved in the data analyses and wrote the manuscript.

## Supplementary Material

giaa114_GIGA-D-20-00133_Original_Submission

giaa114_GIGA-D-20-00133_Revision_1

giaa114_GIGA-D-20-00133_Revision_2

giaa114_Response_to_Reviewer_Comments_Original_Submission

giaa114_Response_to_Reviewer_Comments_Revision_1

giaa114_Reviewer_1_Report_Original_Submission David Burt -- 6/2/2020 Reviewed

giaa114_Reviewer_1_Report_Revision_1 David Burt -- 8/19/2020 Reviewed

giaa114_Reviewer_2_Report_Original_Submission Martien Groenen -- 6/10/2020 Reviewed

giaa114_Reviewer_2_Report_Revision_1 Martien Groenen -- 8/18/2020 Reviewed

giaa114_Supplemental_Files
