# Supplementary material for: Pacific Biosciences assembly with Hi-C mapping generates an improved, chromosome-level goose genome
Source: Gigascience. 2020 Oct 24;9(10):giaa114. doi: 10.1093/gigascience/giaa114 (PMC7585555; doi:10.1093/gigascience/giaa114)
Supplement: giaa114_GIGA-D-20-00133_Revision_1 [file giaa114_giga-d-20-00133_revision_1.pdf]

GigaScience

PacBio assembly with Hi-C mapping generates an improved, chromosome-level goose genome

--Manuscript Draft--

**Manuscript Number:**	GIGA-D-20-00133R1
**Full Title:**	PacBio assembly with Hi-C mapping generates an improved, chromosome-level goose genome
**Article Type:**	Data Note
**Funding Information:**	National Key R & D Program of China (2018YFD0500403) — Prof. Mingzhou Li<br>National Natural Science Foundation of China (U19A2036) — Prof. Mingzhou Li<br>National Natural Science Foundation of China (31872335) — Not applicable<br>National Natural Science Foundation of China (31772576) — Prof. Mingzhou Li<br>National Natural Science Foundation of China (31802044) — Dr. Yan Li<br>China Postdoctoral Science Foundation (2018M643514) — Dr. Yan Li
**Abstract:**	Background:<br>The domestic goose is an economically important and scientifically valuable waterfowl; however, a lack of high-quality genomic data has hindered research concerning its genome, genetics, and breeding. As domestic geese breeds derive from both the swan goose (Anser cygnoides) and the graylag goose (Anser anser), we selected a female Tianfu goose for genome sequencing. We generated a chromosome-level goose genome assembly by adopting a hybrid de novo assembly approach that combined PacBio single-molecule real-time sequencing, high-throughput chromatin conformation capture mapping, and Illumina short-read sequencing.<br>Findings:<br>We generated a 1.11 Gb goose genome with contig and scaffold N50 values of 1.85 Mb and 33.12 Mb, respectively. The assembly contains 39 pseudo-chromosomes (2n = 78) accounting for ca. 88.36% of the goose genome. Compared with previous goose assemblies, our assembly has more continuity, completeness, and accuracy; the annotation of core eukaryotic genes and universal single-copy orthologs has also been improved. We have identified 17,568 protein-coding genes (PCGs) and a repeat content of 8.67% (96.57 Mb) in this genome assembly. W e also explored the spatial organization of chromatin and gene expression in the goose liver tissues, in terms of inter-pseudo-chromosomal interaction patterns, compartments, topologically associating domains, and promoter-enhancer interactions.<br>Conclusions:<br>We present the first chromosome-level assembly of the goose genome . Thi s will be a valuable resource for future genetic and genomic studies on geese .
**Corresponding Author:**	Mingzhou Li, Ph.D.<br>Sichuan Agricultural University<br>Chengdu, Sichuan CHINA
**Corresponding Author Secondary Information:**	
**Corresponding Author's Institution:**	Sichuan Agricultural University
**Corresponding Author's Secondary Institution:**	
**First Author:**	Yan Li

Powered by Editorial Manager® and ProduXion Manager® from Aries Systems Corporation

**First Author Secondary Information:**	
**Order of Authors:**	Yan Li
	Guangliang Gao
	Yu Lin
	Silu Hu
	Yi Luo
	Guosong Wang
	Long Jin
	Qigui Wang
	Jiwen Wang
	Qianzi Tang
	Mingzhou Li, Ph.D.
**Order of Authors Secondary Information:**	
**Response to Reviewers:**	Detailed responses to Reviewers<br>Below, all critiques and suggestions provided by the reviewers are cited in gray italics; our responses are in black. In red are descriptions within the responses that indicate changes in the manuscript. Moreover, all revisions in the manuscript are marked in red.<br>____<br>Reviewer 1<br>This paper reports on the assembly and annotation of the Tianfu goose genome, a female hybrid of A. anser x A. cygnoides. This assembly is a significant improvement on earlier genomes, which were based on short read technologies. This assembly is a hybrid of three technologies: short reads, long reads and HiC maps.<br>Comment 1:<br>MAJOR POINTS<br>1. The assignment of 39 chromosomes to Hi-C scaffolds is very tentative and needs to be validated. For larger scaffold you could establish homology e.g. with chicken chromosomes, which have extensive FISH/cytogenetic data at least for the macrochromosomes. The smaller scaffolds in the HiC analysis could be parts of larger chromosomes - the HiC map suggests some mis-joins. Also in other genome projects very GC-rich, repeat-rich chromosomes (such as microchromosomes) are difficult if not impossible to sequence, and are missing from the assembly. So 39 pseudo-chromosomes are found but these do not equate to the 39 physical chromosomes. This affects conclusions on chromosome number, genome completeness, gene density distribution, distribution of TADs, etc. As a reference goose genome these points need to be addressed.<br>Response 1 :<br>Thank you for this valuable suggestion. We recognize the importance of a reference genome that comprises accurate, physical chromosomes for future genetic and genomic studies on geese. In this study, we generated a 1.11 Gb goose genome with contig and scaffold N50 values of 1.85 Mb and 33.12 Mb, respectively (Table 1). Our assembly contains 39 pseudo-chromosomes (2n = 78), which account for 88.36% of the goose genome; it is a draft goose genome assembly rather than a complete assembly of 39 physical chromosomes. As far as we know, this genome is comparable with other chromosome-level avian genome assemblies (Table 1). (The tables and figures were accessible from Response_sup.pdf at: https://fnca1-my.sharepoint.com/:b:/g/personal/guanglianggaocq_lwh_world/EQSVOZIBkJ9HlaxEnZFufdcBf_eOaki7d4jJkNUz9Yrwpw?e=qcLxYV)<br>We regret that our original description implied a complete genome assembly of 39 chromosomes. We have corrected this point by stating throughout the manuscript that our assembly is a chromosome-level goose genome assembly comprising 39 pseudo-chromosomes.<br><br>OTHER POINTS<br>Comment 1-1:<br>1. This is a chromosome-level assembly make this clear in the text.

	Response 1-1:<br>In accordance with Reviewer 1's suggestion, we have clarified that it is a chromosome-level assembly throughout the manuscript.<br><br>Comment 1-2:<br>2. The hybrid approach used here is good but this is a rapidly evolving field, and is already superseded by technology (Pacbio HiFi now so polishing using short reads not needed) and software (e.g. Lachesis no longer supported).<br>Response 1-2:<br>Thank you for this valuable insight. Certainly, de novo whole-genome assembly approaches change over time, and algorithms adapt in line with evolving sequencing technologies. This allows researchers to generate more continuous, complete, and accurate genome assemblies. To facilitate genomic, genetic, and breeding studies on the goose, we report here an improved, chromosome-level goose genome for the scientific community. We expect that our current goose assembly and annotation will be helpful for researchers in different study fields. Furthermore, we remain committed to assembling a complete and accurate goose genome sequence in the future. For this purpose, we plan to adopt the Pacbio HiFi and Pacbio isoseq technologies, and combine them with extensive FISH and cytogenetic experiments, and genetic map data.<br>Regarding the three software packages, LACHESIS, SALSA2, and 3D-DNA, each have advantages and limitations for de novo genome assembly. Namely, (1) while we employed the LACHESIS software to combine shotgun fragments and short jump mate-pair sequences with Hi-C data (to generate chromosome-scale de novo genome assemblies), LACHESIS has limitations when assembling polyploid genomes [1]. (2) The SALSA2 algorithm does not require that the number of chromosomes are set in advance, which improves the accuracy of scaffolds to a certain extent; however, this algorithm can introduce many clustering/sorting/orientation errors, and few parameters can be adjusted during operation [2]. (3) 3D-DNA corrects errors in the input assembly and then iteratively orients and orders contigs into a single mega-scaffold. This mega-scaffold is then broken, and chromosomal ends are identified based on a Hi-C contact map. A drawback is that the error correction function in this software has not been well applied; in the case of simulated data, the assembly error rate of 3D-DNA is 2–4 times that of SALSA2 [2].<br>To choose an appropriate software for our genome assembly, we randomly selected a subset of our Hi-C data and performed de novo genome assembly using SALSA2, 3D-DNA, and LACHESIS. As the quality metrics of the LACHESIS genome assemblies were the best, we performed the subsequent studies in this paper with the LACHESIS software.<br><br>Comment 1-3:<br>3. The phrase "high-quality" is used throughout the text but not defined - so please define. It is more likely that sequence data is generated (provide QC data on quality) and then software is used to filter out poor data, to leave high-quality data for assembly.<br>Response 1-3<br>Thank you for this useful insight. In our manuscript, the phrase "high-quality" refers to data that was filtered from three sequencing platforms and used for the genome assembly. For the short reads, we employed a Perl script written by our lab to filter the data from the Illumina platform. As a result, the Q20 and Q30 values of the whole genome sequencing data from the Illumina platform were greater than 96.44 % and 93.25 %, respectively (Table 2). (accessible from Response_sup.pdf at: https://fnca1-my.sharepoint.com/:b:/g/personal/guanglianggaocq_lwh_world/EQSVOZIBkJ9HlaxEnZFufdcBf_eOaki7d4jJkNUz9Yrwpw?e=qcLxYV). The Q20 and Q30 values of the Hi-C data used in our study were 97.86 % and 91.84 %, respectively (Table 2). These results suggest that the data used in our genome assembly were "high-quality".<br><br>Comment 1-4:<br>4. For all software, please provide versions and source.<br>Response 1-4:<br>Following Reviewer 1's valuable suggestion, we have added the below descriptions to the supplemental materials (see lines 2–63).<br>Goose genome assembly, annotation and the spatial organization of chromatin in liver tissues analysis by the following software

Powered by Editorial Manager® and ProduXion Manager® from Aries Systems Corporation

	Goose genome were de novo assembled by the following software:<br>(1) FALCON: version 3.1, parameters: length_cutoff = 5000 length_cutoff_pr = 4500;<br>(2) pbsmrtpipe: version smrtlink_5.0.1, default parameters;<br>(3) SSPACE-LongRead: version 1-1, default parameters;<br>(4) PBjelly: version PBSuite_15.8.24, parameters: blasr: -minMatch 8 -minPctIdentity 75 -bestn 1 -nproc 13 -noSplitSubreads;<br>(5) pilon: version pilon-1.18, parameters: -Xmx400G --diploid --threads 30;<br>(6) Lachesis: version-201701, parameters: RE_SITE_SEQ = GATC, CLUSTER_N = 39, CLUSTER_MIN_RE_SITES = 600, CLUSTER_MAX_LINK_DENSITY = 3, CLUSTER_NONINFORMATIVE_RATIO = 0.<br>(7) kallisto: version 0.44.0, parameters: -i -o --bias --rf-stranded.<br>Goose genome were annotated followed the software:<br>(1) GCE: version1.0.0, parameters: -H 1;<br>(2) SOAPdenovo: version2, k-mer size of 59;<br>(3) GAPcloser: version1.12, parameters: -l 150 -p 31;<br>(4) SSPACE: version3.0, default parameters;<br>(5) RepeatMasker: Repeat Masker-open-4-0-6, parameters: -a -nolow -no_is -norna -parallel 1;<br>(6) RepeatModeler: RepeatModeler-open-1.0.11, parameters: -database genome -engine ncbi -pa 15;<br>(7) Tandem Repeats Finder: TRF-407b, parameters: 2 7 7 80 10 50 2000 -d -h;<br>(8) TBLASTN: blast-2.2.26, parameters: -e 1e-05 -F T -m 8;<br>(9) GeneWise: version2.4.1, parameters: -tfor/-trev -genesf -gff;<br>(10) Augustus: version3.2.3, param- eters: –uniqueGeneId = true–noInFrameStop = true–gff3 = on–genemodel = complete–strand = both;<br>(11) GlimmerHMM: version3.0.1, parameters: -g -f;<br>(12) SNAP: snap-2013-11-29, default parameters;<br>(13) Trinity: trinityrnaseq-2.1.1, parameters: –seqType fq-CPU 20–max_memory 200G–normalize_reads–full_cleanup– min_glue 2–min_kmer_cov 2–KMER_SIZE 25;<br>(14) PASA: PASA_r20140417, default parameters;<br>(15) InterPro: version29.0, perl-based version4.8, default parameters;<br>(16) tRNAscan-SE: tRNAscan-SE-1.3.1, default param- eters;<br>(17) INFERNAL: version1.1rc4 (June 2013);<br>(18) BLASTp: blast-2.2.26, parameters: -p blastn -e 1e-10 -v 10000 -b 10000;<br>(19) EVM: VidenceModeler-1.1.1, parameters: –segment- Size 200000–overlapSize 20000;<br>(20) Tophat: tophat-2.0.13, parameters: -p 6–max-intron-length 500000 -m 2– library-type fr-unstranded;<br>(21) Cufflinks: cufflinks-2.1.1, parameters: -I 500000 -p 1–library-type fr-unstranded -L CUFF;<br>(22) BUSCO: version3.0.2, OrthoDBv9_vertebrata;<br>(23) BWA: bwa-0.7.8, parameters: mem -k 32 -w 10 -B 3 -O 11 -E 4 -t 10;<br>(24) SAMtools: samtools-0.1.19, parameters: mpileup mpileup -m 2 -u;<br>(25) RAxML: version 8.0.19, default parameters;<br>(26) CAFÉ: Version 1.6, default parameters;<br>(27) BLASTP: Version 2.2.26, default parameters;<br>(28) PAML: Version 14.7, default parameters;<br>LncRNA and TUCP were annotated followed the software:<br>(1) STAR: version 2.6.0c, default parameters;<br>(2) Cufflinks: version 2.2.1, default parameters;<br>(3) TACO: version 0.7.3, parameters: --filter-min-expr 0.1 --isoform-frac 0.1 --path-kmax 20 --max-paths 20 --filter-min-length 250 --gtf-expr-attr FPKM;<br>(4) taco_refcomp: part of TACO in version 0.7.3, parameters: -o -r -t<br>(5) CPC2: version beta of CPC2, default parameters;<br>(6) transeq: parts of EMBOSS in version 6.6.0, parameters: -sequence -outseq -frame 6 -clean;<br>(7) kallisto: version 0.44.0, parameters: -i -o --bias --rf-stranded.<br>Hi-C data analysis by the following software:<br>(1) Juicer: version 1.8.9, parameters: -C 8000000 -s MboI -p goose.chromosome.sizes -z goose.fa -y goose.MboI.fragment.txt -n 10G;<br>(2) Hi-C Domain Caller, pipeline to call domains from Hi-C experiments: http://chromosome.sdsc.edu/mouse/hi-c/download.html;<br>(3) PSYCHIC: parameters, res: 25000, win: 2000000, chrname: chr*, chrsize: chr*.size, output_prefix: goose.chr*.25000, output_dir: output_directory, input_matrix:

Powered by Editorial Manager® and ProduXion Manager® from Aries Systems Corporation

goose.chr*.25000.normalized.matrix, gene_file: goose.gene.psychic.bed, skip_hierarchy: FALSE.

Comment 1-5:
5. LINE 84: k-mer distribution analysis used to estimate genome size - provide reference, software, method - also mention other QC estimates (repeats, polyploidy etc).
Response 1-5:
Thank you for this useful comment. To estimate genome size, repeat regions, heterozygosity, and polyploidy prior to assembling the goose genome, we employed survey software based on K-mer (k = 17) frequency distributions. This predicted the genome size, repeat ratio, and heterozygosity to be 1277.1 Mb, 39.8%, and 0.4%, respectively. In the K-mer analysis, the goose data demonstrated a distribution typical of a diploid genome (Figure 1), (accessible from Response_sup.pdf at: https://fnca1-my.sharepoint.com/:b:/g/personal/guanglianggaocq_lwh_world/EQSVOZIBkJ9HlaxEnZFufdcBf_eOaki7d4jJkNUz9Yrwpw?e=qcLxYV), showing only a single major peak—which could be used to estimate the genome size. In addition, the first minor peak represents the level of heterozygosity and the second minor peak represents the level of repeat sequence [3].

Comment 1-6:
6. LINE 91: Lachesis old software no longer supported - why not used SALSA2 or 3D-DNA?
Response 1-6:
Thank you for raising this important point. Please see Response 1-2 for a discussion of this issue.

Comment 1-7:
7. Figure S1: Hi-C map suggests lots of mis-joins, have you checked and manually corrected?
Response 1-7:
Thank you for this valuable comment. In accordance with this point, we also identified mis-joins in the Hi-C map, which suggests that these regions of the genome might be repetitive, GC-rich, or contain structural variation. As mentioned above, we are committed to assembling a complete and accurate goose genome sequence, and in future work aim to focus on these “mis-joins” using the latest technologies and corresponding assembly algorithms.

Comment 1-8:
8. LINES 109-111, again used the term "high-quality" for a mix of genomes, Human, Mouse, Chicken probably but duck, turkey and zebra finch are draft and not high-quality genomes.
Response 1-8:
Thank you for this insight. We have changed “high-quality" to “chromosome-level” in line 109. As described in Table 1, while the contig N50 values of zebra finch, duck, and turkey are 12.0 Mb, 36.80 Kb, and 27 Kb, respectively, these genomes were assembled into chromosome-level assemblies with the aid of other technologies, such as RH mapping and FISH (Table 1).

Comment 1-9:
9. LINES 114-117, pooled RNAseq used, so how can you quantify gene expression later in paper? Needs deconvolution of pooled samples - was this done? For annotation Pacbio isoseq would be better.
Response 1-9:
RNA-seq prediction is a commonly used method for improving genome annotation, correcting predicted gene structures, detecting new alternative splicing isoforms, and discovering new genes and transcripts. In this updated manuscript, we used data from the pooled RNA-seq analysis (abdominal fat, brain, duodenum, heart, liver, lung, muscular stomach, ovary, pancreas, pectoral muscle, and spleen) only for the annotation of the goose genome and not for gene expression quantification or for the spatial analysis of chromatin organization. Accordingly, we did not perform a deconvolution analysis of the pooled RNA-seq sample.
Nevertheless, we sincerely thank Reviewer 1 for this reminder on the correct usage of pooled RNA-seq data. We have now realized that our pooled RNA-seq data were

inaccurately used to explore the spatial organization of chromatin in goose liver tissue. To address this, we downloaded three RNA-seq datasets from liver tissues (Accession numbers: GSM3374538, GSM3374539, GSM3374540), which had been generated from the same goose strain used in our study [4]. We then quantified gene expression in these three samples and used these results to update all the content related to gene expression in our study, in terms of compartments and promoter-enhancer interactions. We have revised the main text and Figures S7–S11 and Figure 2 accordingly.
Regarding Pacbio, we also thank Reviewer 1 for raising this point. Certainly, the long reads from the Pacbio isoseq platform could better annotate complete transcripts in genomes. We aim to adopt this method, and other newly developed methods, when we update the quality of the goose genome assembly or annotation in future work.

Comment 1-10:
10. Prediction of lncRNAs from assembly of short read RNA-seq is known to be poor, so LINEs 121-124, where 3,287 lncRNAs are predicted needs to be taken with caution.
Response 1-10:
Thank you for this valuable suggestion. We agree that longer reads from PacBio and ONT offer advantages for resolving complex features in transcriptomes when compared to short read RNA-seq. For example, read length is the major advantage of Iso-Seq cDNA transcript sequencing and Oxford Nanopore direct RNA-seq, which can both capture entire transcripts.
Compared with mRNAs, which can be annotated by a combination of ab initio and homologous assembly approaches, lncRNAs are not conserved among species. lncRNAs can thus only be identified by transcript data, without the aid of homology. Long reads can be helpful for the identification and annotation of lncRNAs, and in future work, we will adopt this strategy. In this study, we identified lncRNAs by analyzing the transcript data from short read RNA-seq only, and we have clarified this point in lines 124–127 of the main text.

Comment 1-11:
11. LINE 160, goose and duck diverged 32 Mya, how does this estimate compare with other data sources?
Response 1-11:
Thank you for this important comment. In this study, we first downloaded the reported divergence times between each pair of species (e.g. chicken and turkey) from the TimeTree website (http://www.timetree.org/). These divergence times are estimated on the basis of single-copy gene families via a Bayesian algorithm called mcmctree, within the software "PAML (http://abacus.gene.ucl.ac.uk/software/paml.html)". We also used well-established divergence times to further adjust the estimated divergence times of other species and improve the accuracy of our results. The divergence times predicted in our study were consistent with two previous reports: 20.8 (12.9-32.7) Mya in Lu et al. [5]; and 30.0 (21.4-38.6) Mya in Gao et al. [6].

Comment 1-12:
12. sections (b-d) interesting predictions from phylogenetic analyses, but all speculation, there is no other data provided to back up these predictions.
Response 1-12:
Thank you for raising this point. The purpose of our article was to supply a valuable resource for future genetic and genomic studies on geese. Accordingly, we endeavored to explore the general characteristics of the goose genome by performing common general analyses—such as the expansion and contraction of gene families, and the identification of genes under positive selection. In the future, experiments on gene function will help to resolve the speculations and predictions we have presented here. We have revised the main text in lines 171, 191-192 and 198–211 to address this issue.

Comment 1-13:
13. LINE 192, PAML Codeml analysis is crude, and does not correct for multiple testing, with 17K genes tested there is a high false positive rate, was there any correction for multiple testing, if not please correct.
Response 1-13:
Thank you for this valuable comment. In our work, only single-copy genes (n = 2389) were used for the identification of genes under positive selection. We did not use all 17K genes. After we calculated the p-value for each of the candidate positively

selected genes using PAML, we further adjusted the p-values (e.g. using the Bonferroni method, a method for multiple testing) to reduce false-positive results.

Comment 1-14:
14. LINE 202, the TAD analysis is restricted to liver tissue.
Response 1-14:
Thank you for raising this point. We explored the spatial organization of chromatin and gene expression in goose liver tissue only, with regard to topologically associating domains (TAD). TADs were largely invariable across tissues or species. We have clarified this point in lines 29, 213, 224, and 229.

Comment 1-15:
15. LINES 203-204, macs and mics form sub-domains in the nucleus. Figure 4 needs more explanation, poor figure.
Response 1-15:
We have replotted Figure 4 (named Figure 2 in revised manuscript) and increased the resolution of this figure. We have also added additional explanation and changed the figure legend as follows: "Dendrogram of inter-pseudo-chromosome interaction patterns generated by the average linkage algorithm. Heatmap shows the inter-pseudo-chromosome interaction probability, as generated by calculating the observed/expected contact frequencies for chromosome pair i, j. This is overlaid on a histogram showing pseudo-chromosome length, number of protein-coding genes, and GC percentage".

Comment 1-16:
16. LINE 205, define compartments A and B, how are these defined in Hi-C data?
Response 1-16:
Thank you for this valuable query. We have now added an explanation of the methods used for identifying compartments A and B, as well as the methods for how the spatial organization of chromatin and gene expression were explored in the goose liver tissues (see lines 64–92 in the supplemental materials). These methods relate to inter-pseudo-chromosomal interaction patterns, topologically associating domains, promoter-enhancer interactions, and gene expression quantification.

Comment 1-17:
17. LINE 206, how were TSS (transcription start sites, not defined in the set of abbreviations, please add) defined? I assume based on the pooled short read RNA-seq data. If correct, this is a poor data set, since the assembly of transcripts based on short read data only defines the most 5' RNA sequenced. So misses any internal TSS, does not correct for degraded RNA, etc.
Response 1-17:
Thank you for this comment. We agree that basing the definition of TSS sites on short read data would be inaccurate. We apologize for the ambiguous TSS-related description in our manuscript. We have changed the description in line 221 to 223: "the number of protein-coding genes (PCGs) in each 100-Kb bin with at least 50% percentage overlapped with a gene was counted. The number of PCGs was significantly correlated with PC1 values".

Comment 1-18:
18. LINE 213, gene expression levels based on pooled RNAseq data is a very poor dataset, should deconvolute or at least have a high-quality liver RNA set.
Response 1-18:
As described above (see Response 1-9), to address this issue we downloaded three additional RNA-seq datasets that were restricted to liver tissue (Accession numbers: GSM3374538, GSM3374539, GSM3374540). These datasets derive from the same goose strain as used in our study, and on the basis of a new analysis of these data we have updated all the sections of our manuscript related to gene expression. Specifically, we have changed the following description in lines 223–225: "the transcripts per kilobase millions (TPMs) of PCGs located in A compartments were significantly higher than those in B compartments", to: "the transcripts per kilobase millions (TPMs) of PCGs located in A compartments were consistently higher than PCGs in B compartments in three liver tissues". We have also changed lines 229–230 from: "found that gene expression levels positively correlated with the number of PEIs", to: "found that gene expression levels positively correlated with the number of

associated enhancers in all three liver tissues".

Reviewer 2
The manuscript describes a highly contiguous genome assembly of the goose genome and provides a significant improvement of the assembly of this bird. The results are described very clearly, and the data has been made publicly available. The analyses done are rather straightforward, and much more could have done with the interesting data generated in this study, which to me seems a missed opportunity.
The authors decide to sequence an F1 animal that is a cross between A. anser and A. cygnoides. I wonder why the authors did not use Illumina sequencing to sequence the genome of the two parents. This would have allowed the generation of two haplotype specific assemblies and the Comparison between the genomes of these two different sub-species. Also, no indication is given for the number of variants see in this bird, which would also have provided a good indication of the sequence divergence between these two sub-species. Finally, the realignment of the short-read Illumina sequences, provides a way to estimate the number of sequence errors still present in the final assembly (seen as homozygous SNPs and indels).
Response 2：
We apologize, it is apparent that our description of the Tianfu goose used for genome assembly in this study was not clear. Domesticated geese derive from the swan goose (Anser cygnoides) and the graylag goose (Anser anser). The Tianfu goose is a recognized breed that has originated from crosses between the domestic Landes goose (A. cygnoides) and the Sichuan white goose (A. anser), rather than the F1 animal crossed between A. anser and A. cygnoides. The Tianfu goose is a developed breed with many outstanding characteristics, such as excellent egg-laying performance, a fast growth rate, and strong adaptability. These characteristics are why we selected the Tianfu goose for this study.
Until now, a high-quality reference goose genome has not been available. To provide a valuable resource for future genetic and genomic studies on geese, and facilitate related research fields, our manuscript presents the first chromosome-level assembly of the goose genome. With reference to human and mouse research, in future studies we also aim to perform haplotype-resolved genome assemblies of F1 geese and parent animals, and compare differences between breeds.
Regarding the estimation of sequence errors, after we obtained our final goose assembly, we realigned the short read Illumina sequences with BWA software, and called SNPs and InDels using GATK software. As can be seen in Table 3, the proportions of homologous SNPs and InDels identified (which often reflect assembly errors) were extremely low, which indicates that our final assembly is of “high-quality”.

Table 3: Homologous SNPs and InDels in the goose genome.
CategoryNumberProportion (%)
Homologous SNPs23,3240.0021
Homologous InDels8,7260.00078

OTHER POINTS
Comment 2-1:
Figure 1 and figure 2 are not very informative and I suggest moving these to the supplementary information
Response 2-1:
We agree with this suggestion from Reviewer 2. We have removed Figure 1 and Figure 4 to the supplementary figures, and have reordered the sequence of the corresponding supplementary figures.

Comment 2-2:
Line 89-90: The authors refer to table S1 in relation to the correction of sequencing errors. However, this table does not provide any information about sequencing errors.
Response 2-2:
We apologize for this inaccurate description. We have revised the main text to address this error, see lines 87–88.

Comment 2-3:
Line 90-91: The authors refer to table S2 and Fig S1. However, table S2 shows a summary of the pseudo chromosomes, not of the Hi-C scaffolds. Furthermore, in table S1 the authors show that there are 2123 Hi-C scaffolds. Please elaborate and clarify.

Response 2-3:
We regret the error in this description. Indeed, we state the length of the pseudo-chromosomes in the goose genome in Table S2, and present the Hi-C interaction contact heatmap of the pseudo-chromosomes in Figure S3. There are 2123 scaffolds in our goose genome assembly. This includes 68 scaffolds of 200bp to 2000bp, 2016 scaffolds of 2000bp to 350000bp, and 39 pseudo-chromosomes that are greater than 1Mb.

Comment 2-4:
Line 119-121: Again, the reference to the table/figure does not seem to match very well with the information in the text. I also suggest to add the number of PCG's to table 3. Also, does figure 2 only show the TSS for PCG or does it also include those for the lncRNAs.
Response 2-4:
Thank you for this valuable suggestion. Accordingly, we have added the number of PCGs to Table 3. In Figure 2, we show only the TSSs for PCGs. We have redrawn Figure 2.

Comment 2-5:
Line 128: I am confused by the comment that the current assembly has more scaffolds. Given that the assembly is improved with higher N50 values for the contigs and scaffolds, I would assume that the number would be smaller.
Response 2-5:
Thank you for raising this point. To display the quality of the genome assemblies, we analyzed the distribution of their scaffold lengths. In our goose genome, with the exception of the 39 pseudo-chromosomes, lengths of scaffolds are distributed from 2kb to 350kb (Table 4). This indicates that our assembly contains 39 pseudo-chromosomes (longer than 1Mb) and 2016 scaffolds (of lengths ranging from 2kb to 350kb). To supply more information for researchers, we did not filter the 2kb–350kb scaffolds from our genome assembly data. As a result, we have reported more scaffolds in this study than were reported in two previous studies. However, as the 39 pseudo-chromosomes we assembled account for 88.36% of the genome (Table 4, Figure 2), (The tables and figures were accessible from Response_sup.pdf at: https://fnca1-my.sharepoint.com/:b:/g/personal/guanglianggaocq_lwh_world/EQSVOZIBkJ9HlaxEnZFufdcBf_eOaki7d4jJkNUz9Yrwpw?e=qcLxYV), this suggests that our genome assembly is an improvement on previous goose assemblies.

Comment 2-6:
Line 129-131: This statement is not supported by table 3. In fact, the other studies seem to have annotated more gene sequences than the current assembly.
Response 2-6:
Thank you for this useful comment. In this study, we annotated more repeat regions (8.67%) (Table S3) and exon sequence regions (26,883,354bp, 2.41%) (Table 3) than in previous studies (Table 3). This suggests that we have generated an improved genome assembly and annotation. We have revised lines 132–133 of the manuscript to address this point.

Comment 2-7:
Line 195-196: "… indicating that disease resistance may help …..". I don't think this statement is supported by the results and tends to be mere story telling.
Response 2-7:
Thank you for identifying this issue. In lines 198–211, we have revised the original text as follows: “Some of these PSGs, such as GCH1 (GTP-cyclohydrolase I), are associated with parkinsonism, dystonia, and phenylketonuria disease in humans [7, 8]. They also play a role in adaptation to high-altitude environments in humans, where they relate to a lower hemoglobin level, nitric oxide concentration, and oxygen saturation in the blood. Furthermore, previous studies have shown GCH1 divergence between human populations living at different altitudes [9]. Selection acting on GCH1 in goose is likely to be related to their adaption to high-altitude or migratory habitats. SNW1 (SNW1 Domain Containing 1) is involved in the Nuclear Factor Kappa B pathway and is associated with oculopharyngeal muscular dystrophy disease [10, 11]. The depletion of this gene in breast cells leads to the induction of apoptosis, while the overexpression of this gene impedes neural crest development [12]. Selection acting on SNW1 in goose suggests that it may confer protection from diseases and aid

	adaptation in changeable environments. POU2F3 is pivotal in the discrimination of taste qualities, such as sweet, umami and bitter characteristics. Deficiency in this gene in mice alters their electrophysiology and behavioral responses to taste characters [13, 14]. Selection acting on POU2F3 in goose is likely to be related to a requirement for seeking food in variable migratory habitats."<br><br>References<br>1.Burton JN, Adey A, Patwardhan RP, Qiu R, Kitzman JO, Shendure J. Chromosome-scale scaffolding of de novo genome assemblies based on chromatin interactions. Nat Biotechnol. 2013; 31(12): 1119-1125.<br>2.Ghurye J, Rhie A, Walenz BP, et al. Integrating Hi-C links with assembly graphs for chromosome-scale assembly. PLoS Comput Biol. 2019; 15(8): e1007273.<br>3.Liu B, Shi Y, Yuan J, et al. Estimation of genomic characteristics by analyzing k-mer frequency in de novo genome projects. 2013.<br>4.Wang G, Jin L, Li Y, et al. Transcriptomic analysis between Normal and high-intake feeding geese provides insight into adipose deposition and susceptibility to fatty liver in migratory birds. BMC Genomics. 2019; 20(1): 372.<br>5.Lu L, Chen Y, Wang Z, et al. The goose genome sequence leads to insights into the evolution of waterfowl and susceptibility to fatty liver. Genome Biol. 2015; 16(1): 89.<br>6.Gao G, Zhao X, Li Q, et al. Genome and metagenome analyses reveal adaptive evolution of the host and interaction with the gut microbiota in the goose. Sci Rep. 2016; 6: 32961.<br>7.Yoshino H, Nishioka K, Li Y, et al. GCH1 mutations in dopa-responsive dystonia and Parkinson's disease. J Neurol. 2018; 265(8): 1860-1870.<br>8.Gu Y, Lu K, Yang G, et al. Mutation spectrum of six genes in Chinese phenylketonuria patients obtained through next-generation sequencing. PLoS One. 2014; 9(4): e94100.<br>9.Guo YB, He YX, Cui CY, et al. GCH1 plays a role in the high-altitude adaptation of Tibetans. Zool Res. 2017; 38(3): 155-162.<br>10.Verma S, De Jesus P, Chanda SK, Verma IM. SNW1, a Novel Transcriptional Regulator of the NF-κB Pathway. Mol Cell Biol. 2019; 39(3): e00415-18.<br>11.Tolde O, Folk P. Stress-induced expression of p53 target genes is insensitive to SNW1/SKIP downregulation. Cell Mol Biol Lett. 2011; 16(3): 373-384.<br>12.Wu MY, Ramel MC, Howell M, Hill CS. SNW1 is a critical regulator of spatial BMP activity, neural plate border formation, and neural crest specification in vertebrate embryos. PLoS Biol. 2011; 9(2): e1000593.<br>13.Huang YH, Klingbeil O, He XY, et al. POU2F3 is a master regulator of a tuft cell-like variant of small cell lung cancer. Genes Dev. 2018; 32(13-14): 915-928.<br>14.Matsumoto I, Ohmoto M, Narukawa M, Yoshihara Y, Abe K. Skn-1a (Pou2f3) specifies taste receptor cell lineage. Nat Neurosci. 2011; 14(6): 685-687.
**Additional Information:**	
**Question**	**Response**
Are you submitting this manuscript to a special series or article collection?	No
**Experimental design and statistics**<br><br>Full details of the experimental design and statistical methods used should be given in the Methods section, as detailed in our Minimum Standards Reporting Checklist. Information essential to interpreting the data presented should be made available in the figure legends.	Yes

Have you included all the information requested in your manuscript?	
**Resources**<br><br>A description of all resources used, including antibodies, cell lines, animals and software tools, with enough information to allow them to be uniquely identified, should be included in the Methods section. Authors are strongly encouraged to cite Research Resource Identifiers (RRIDs) for antibodies, model organisms and tools, where possible.<br><br>Have you included the information requested as detailed in our Minimum Standards Reporting Checklist?	Yes
**Availability of data and materials**<br><br>All datasets and code on which the conclusions of the paper rely must be either included in your submission or deposited in publicly available repositories (where available and ethically appropriate), referencing such data using a unique identifier in the references and in the “Availability of Data and Materials” section of your manuscript.<br><br>Have you have met the above requirement as detailed in our Minimum Standards Reporting Checklist?	Yes

Manuscript

Click here to access/download;Manuscript;GigaScience_Manuscript.docx

PacBio assembly with Hi-C mapping generates an improved, chromosome-level goose genome

Yan Li[1, †], Guangliang Gao[1, 2, †, *], Yu Lin[1, †], Silu Hu[1], Yi Luo[1], Guosong Wang[1,3], Long Jin[1], Qigui Wang[2], Jiwen Wang[1], Qianzi Tang[1], Mingzhou Li[1, *]

[1]Institute of Animal Genetics and Breeding, College of Animal Science and Technology, Sichuan Agricultural University, Chengdu 611130, China;

[2]Institute of Poultry Science, Chongqing Academy of Animal Sciences, Chongqing 402460, China;

[3]Department of Animal Science, Texas A&M University, College Station 77843, United States of America

[†] These authors contributed equally to this paper.

[*] Corresponding author(s): Guangliang Gao: guanglianggaocq@hotmail.com; Mingzhou Li: mingzhou.li@sicau.edu.cn.

Abstract

Background:

The domestic goose is an economically important and scientifically valuable waterfowl; however, a lack of high-quality genomic data has hindered research concerning its genome, genetics, and breeding. As domestic geese breeds derive from both the swan goose (*Anser cygnoides*) and the graylag goose (*Anser anser*), we selected a female Tianfu goose for genome sequencing. We generated a chromosome-level goose genome assembly by adopting a hybrid *de novo* assembly approach that combined PacBio single-molecule real-time sequencing, high-throughput chromatin conformation capture mapping, and Illumina short-read sequencing.

Findings:

We generated a 1.11 Gb goose genome with contig and scaffold N50 values of 1.85 Mb and 33.12

Mb, respectively. The assembly contains 39 pseudo-chromosomes (2n = 78) accounting for ca. 88.36% of the goose genome. Compared with previous goose assemblies, our assembly has more continuity, completeness, and accuracy; the annotation of core eukaryotic genes and universal single-copy orthologs has also been improved. We have identified 17,568 protein-coding genes (PCGs) and a repeat content of 8.67% (96.57 Mb) in this genome assembly. We also explored the spatial organization of chromatin and gene expression in the goose liver tissues, in terms of inter-pseudo-chromosomal interaction patterns, compartments, topologically associating domains, and promoter-enhancer interactions.

Conclusions:

We present the first chromosome-level assembly of the goose genome. This will be a valuable resource for future genetic and genomic studies on geese.

Key Words: goose genome, chromosome-length assembly, hybrid *de novo* assembly approaches, annotation, Pacbio, Hi-C

Data description

Context

The goose is a member of the family Anatidae and is an economically important waterfowl with distinctive characters. Domesticated geese derive from the swan goose (*Anser cygnoides*) and the graylag goose (*Anser anser*)[1], and approximately 6,000 years of artificial selection have led to significant alterations in their body size, reproductive performance, egg production, feather color, and other features[2]. Currently, more than 181 domesticated breeds are reared globally to supply meat, eggs, and valuable byproducts (feathers, fatty liver) for human consumption[2,3,4]. The domestic goose is also well suited to sustainable production practices because fiber can form part of its diet, which then lessens competition for human food[5]. Its excellent disease resistance and behavioral patterns also allow for large-scale

farming and easy management[6]. Interestingly, despite the liver weight of goose increasing 5–10 times after two to three weeks of overfeeding, the amount of fat in hepatic cells (and other biomedical parameters) returns to normal levels when overfeeding ceases. This suggests that the goose liver could provide a novel animal model for the study of human non-alcoholic fatty liver disease[6].

The goose was one of the earliest animals to be domesticated[2,7], and wide-ranging genomic and breeding research has been conducted to study its domestication process and the unique morphological and physiological features of these animals. For example, recently published goose genome sequences have been assembled into scaffolds using short reads from the Illumina platform[8,9]; however, the genetic basis of the fatty liver of goose and their selective breeding remains largely unknown. To address such issues, a high-quality genome sequence is required. Currently, there are many advantages to using hybrid *de novo* assembly approaches to improve the quality of genome assemblies. This is because short, accurate reads from the Illumina platform can be combined with the longer, less accurate reads generated by the single-molecule real-time (SMRT) sequencing platform[10]. With Hi-C, linking information can then be ordered and oriented into scaffolds, after which assembly errors can be identified and corrected[11]. This approach has been applied to improve the genome assemblies of many species, including humans[12], goats[13], rockfish[14], *Aedes aegypti*[11], and barley[15].

Here, we have generated a chromosome-level goose assembly with chromosome-length scaffolds by adopting a hybrid *de novo* assembly approach using a combination of short reads from the Illumina platform, long reads from the PacBio platform, and Hi-C-based chromatin interaction maps. Our chromosome-level goose genome comprises longer scaffolds than currently available goose genome assemblies, and these scaffolds are of a higher-quality and are more continuous and accurate. Our new genome assembly thus provides a valuable resource for exploring the molecular basis of the

morphological and physiological features of the goose, and will facilitate further genomic, genetic, and breeding studies of this domesticated waterfowl.

Methods

a) Sample collection and sequencing

We extracted genomic DNA from the liver tissue of a healthy adult female (136 days old) from the Tianfu goose maternal line, which was provided by the Experimental Farm of Waterfowl Breeding of Sichuan Agricultural University (Chengdu, Sichuan, China; **Figure S1**). We then carried out single-molecule real-time DNA sequencing of ca. 20-kb inserts using the PacBio Sequel platform. This yielded approximately 84.31 Gb of high-quality sequencing data that were used to initially assemble the genome (**Table 1**). Next, 149.70 Gb of high-quality sequencing data were generated from a 350-bp insert size Hi-C library, as previously reported[13]. Finally, 350-bp paired-end libraries constructed from the same genomic DNA were sequenced on the Illumina HiSeq platform, producing a further 181.52 Gb of sequence data. In total, we obtained approximately 415.53 Gb sequencing data (ca. 324.63× coverage) for our chromosome-level goose genome assembly (**Table 1**).

b) *De novo* assembly of the goose genome

The size of the goose genome was estimated by k-mer distribution analysis to be 1.28 Gb. To assemble the genome, we first performed an initial assembly with the PacBio long-reads alone, using Falcon[16] software. We used the pbsmrtpipe pipeline of the smrtlink software to assembly the genome sequence, which resulted in a draft assembly with a contig N50 of 1.72 Mb (**Table S1**). Next, we used the single-molecule sequence reads to scaffold these contigs and fill gaps, using SSPACE-Long[17] and PBJelly[18], respectively. Pilon[19] software was then used to map the short reads to the assembly (**Table S1**). Finally, 39 pseudo-chromosomes were assembled with the Hi-C reads were aligned using Lachesis[20]

software (**Table S2, Figure S2**); this is consistent with the number of goose chromosomes (2n = 78) reported in previous studies[21]. With these methods, we generated a chromosome-level goose assembly with a contig N50 of 1.85 Mb and scaffold N50 of 33.12 Mb (**Table 2**). The average GC content is 42.15% and the total genome size is 1.11 Gb, which is consistent with previous studies[8,9] and suggests that our goose assembly is reliable.

c) Repeat sequence and gene annotation

De novo methods and homology-based approaches were used to annotate the repeat content of the goose genome. First, we used *ab initio*-prediction software, including LTR-finder[22], RepeatMolder[23], and RepeatScout[24], to perform *de novo* annotation of the genome. For homology-based predictions, we identified repeat regions across species in published RepBase sequences[25] using RepeatMasker[26] and RepeatProteinMask[27] software. Combined with these results, the repeat region of the goose genome was further predicted with RepeatMasker software. From these analyses, we identified 92.11 Mb of repetitive DNA (**Table S3**) accounting for 8.67% of our assembly, which is much higher than has been reported in previous studies[8,9]. Long interspersed nuclear elements (LINEs) were the most abundant repeat element identified, representing 6.83% of the genome. The proportion of LINE repetitive sequences identified in this study was also higher than has been reported in two previous goose genome assemblies (**Table S3**). We performed PCGs annotation by combining *ab initio*-based, homology-based, and RNA-sequencing-based prediction methods. First, GenScan[28], Geneid[29], and Augustus[30] were used for *ab initio*-based predictions. Next, we selected six chromosome-level genomes, namely *Homo sapiens* (GCF_000001405.39), *Mus musculus* (GCF_000001635.26), *Gallus gallus* (GCF_000002315.6), *Anas platyrhynchos* (GCF_003850225.1), *Meleagris gallopavo* (GCF_000146605.3), and *Taeniopygia guttata* (GCF_003957565.1), to use for homology-based annotation of our goose chromosome-level assembly

genome using TBLASTN[31] and GeneWise[32] software. We found 8,255 common orthologous groups across these seven species (**Figure S3**). To optimize genome annotation, total RNA was extracted from 11 samples (abdominal fat, brain, duodenum, heart, liver, lung, muscular stomach, ovary, pancreas, pectoral muscle, and spleen) taken from the same individual whose DNA was used for the chromosome-level genome assembly. We pooled equal amounts of the total RNA from each of the 11 tissues and then performed RNA-seq on this pooled sample using the Illumina platform. After filtering, these data were used to annotate protein-coding regions of the genome assembly using Trinity[33] and TopHat[34]. Finally, the predictions from each method described above were integrated using EVM[35]; overall, 17,568 PCGs were predicted (**Table 3, Figure S4**). To identify long noncoding RNAs (lncRNAs), the goose genome reads were aligned by STAR[36] and subjected to Cufflinks[37] and TACO[38] for assembly and filtering. CPC2[39] was then applied to perform coding potential analysis, and PfamScan[40] was used to check for domain hits against Pfam31-A[41]. After removing all likely domains, 3,287 lncRNAs only by ab initio assembly method and 542 transcripts of uncertain coding potential (TUCP) were identified, the long reads will be helpful to improve the identification and annotation of the lncRNA and TUCP in goose genome.

Data validation and quality control

a) Assessment of genome assembly completeness

Our assembly has more scaffolds and fewer contigs, and significantly improved contig and scaffold N50 values, than the goose genome assemblies presented in two previous studies (**Figure 1**). Moreover, we have annotated more repeat (Table S3) and exons sequence regions (Table 3) than these previous studies (**Table 3**), which suggests that we have generated an improved genome assembly and annotation. The 39 pseudo-chromosomes described in our study account for 88.36% of the assembled genome and

are longer than those previously reported[8,9], again indicating that our chromosome-level goose genome represents a significant improvement on previous work. The GC content of our genome assembly is 42% and the size of the genome is 1.11 Gb (**Table 2**). This is comparable to the sizes reported for the two previously constructed goose genomes[8,9] and is characteristic of avian genomes[42]. We also mapped short-insert paired-end reads (350 bp) to our chromosome-level goose genome and obtained mapping and coverage rates of 97.25% and 99.71%, respectively. Finally, we downloaded 19 wild goose resequencing[43] datasets from public databases and mapped them to our assembly, and to the two earlier draft goose genomes. We found that the mapping rate of our chromosome-level goose assembly was higher than that of the previously assembled genomes (**Table S4**), indicating that it is more contiguous. Taken together, these results demonstrate the improvements made by our study in the assembly and annotation of the goose genome, in comparison to previous studies[8,9].

To evaluate the completeness of our chromosome-level genome assembly, we determined the number of conserved eukaryotic and universal genes present in our assembly by applying the core eukaryotic genes mapping approach software (CEGMA) and using a set of benchmarking universal single-copy orthologs (BUSCO). We found that 211 of the 248 (85.08%) core eukaryotic genes and 2,586 (97%) of the universal single-copy orthologs were assembled in our genome. Compared with previous studies, this suggests that our genome assembly is more complete than previous drafts of the goose genome[8,9].

To explore the hypothesis that the leptin gene was lost from goose[8], we downloaded leptin sequences from avian and mammal genomes to use as reference sequences in BLASTP searches of our newly assembled goose genome. We found no sequences similar to leptin in our chromosome-level goose assembly. Furthermore, although the human genome region that contains the leptin gene (chromosome

7, 126.0 to 129.4 Mb) aligned with the goose genome, we did not find a sequence similar to the leptin gene in this region. These results confirm the previous finding that the leptin gene is not present in the goose genome[8].

b) Phylogenetic tree and lineage-specific gene families

Using OrthoMCL[44], 16,157 orthologous gene families across 17 species (ostrich, duck, goose, chicken, turkey, saker, red-legged seriema, African crowned crane, pelican, little egret, crested ibis, cormorant, great crested grebe, pigeon, woodpecker, zebra finch, and lizard) were identified. Based on 2,389 shared single-copy ortholog gene clusters, we constructed a maximum likelihood phylogenetic tree using the RAxML software[45]. This revealed that goose and duck diverged about 31.60 million years ago (Mya), which is comparable to the divergence time of chicken and turkey (32.33 Mya; **Figure S5**) and consistent with the previous studies [8, 9]. We also noted that lineage-specific genes in the goose genome were significantly enriched for olfactory receptor activity (GO:0004984, p = 3.85×10^{-24}), G protein-coupled receptor activity (GO:0004930, p = 6.67×10^{-13}), and integral component of membrane (GO:0016021, $p = 0.01$; **Table S5**). As a migratory bird, the goose is adapted for long-distance migration, which exposes them to a diversity of food as they seek out ideal habitats. We propose that such influences might strengthen the interactions between odorants and the receptors of the olfactory mucosa, and could underlie receptor family evolution in the goose genome.

c) Expansion and contraction of gene families

The expansions and contractions of gene clusters in the goose genome were identified in comparison to nine other avian genomes using the CAFE program[46]. We found 839 expanded gene families (**Table S6**) and 2,193 contracted gene families (**Table S7**). Interestingly, the expanded gene families were mainly enriched for olfactory receptor activity (GO:0004984, p = 8.58×10^{-51}), G protein-coupled receptor

activity (GO:0004930, p = 5.81×10^{-25}), and integral component of membrane (GO:0016021, p = 3.20×10^{-6}), which is consistent with the results from our analysis of lineage-specific genes (**Table S5**). This further confirms that the migratory adaptations of the goose are reflected by unique characteristics in the goose genome that contrast with those of nonmigratory birds. Other expanded gene families were enriched for ATPase-coupled transmembrane transporter activity (GO:0042626, p = 1.96×10^{-06}), NAD(P)+-protein-arginine ADP-ribosyl transferase activity (GO:0003956, p = 3.20×10^{-04}), ATPase activity (GO:0016887, p = 8.28×10^{-05}), and aspartic-type endopeptidase activity (GO:0004190, p = 9.63×10^{-06}; **Table S6**), while gene families contracted in the goose were significantly enriched for transmembrane transport (GO:0055085, p = 8.30×10^{-04}), ion channel activity (GO:0005216, p = 1.87×10^{-9}), ion transmembrane transport (GO:0034220, p = 5.30×10^{-6}), and ATPase-coupled intramembrane lipid transporter activity (GO:0140326, p = 8.60×10^{-10}; **Table S7**). As these pathways are related to ATP utilization, ATP production, and energy regulation, these data support a previous finding that goose energy metabolism is different from that in other avian species[47]. This feature of the goose is possibility related to its migratory habits and artificial selection—the goose is unique among migratory birds because of its large body size, which requires much energy for long-distance, high altitude flying[48].

d) Genes under positive selection

We identified 52 positively selected genes (PSGs) in the goose genome based on orthologous genes from the 17 species above, using a branch-site model and F3x4 codon frequencies in Codeml (**Table S8**). Some of these PSGs, such as *GCH1* (GTP-cyclohydrolase I), are associated with parkinsonism, dystonia, and phenylketonuria disease in humans[49, 50]. They also play a role in adaptation to high-altitude environments in humans, where they relate to a lower hemoglobin level, nitric oxide concentration, and

oxygen saturation in the blood. Furthermore, previous studies have shown *GCH1* divergence between human populations living at different altitudes[51]. Selection acting on *GCH1* in goose is likely to be related to their adaption to high-altitude or migratory habitats. *SNW1* (SNW1 Domain Containing 1) is involved in the Nuclear Factor Kappa B pathway and is associated with oculopharyngeal muscular dystrophy disease[52, 53]. The depletion of this gene in breast cells leads to the induction of apoptosis, while the overexpression of this gene impedes neural crest development[54]. Selection acting on *SNW1* in goose suggests that it may confer protection from diseases and aid adaptation in changeable environments. *POU2F3* is pivotal in the discrimination of taste qualities, such as sweet, umami and bitter characteristics. Deficiency in this gene in mice alters their electrophysiology and behavioral responses to taste characters[55,56]. Selection acting on *POU2F3* in goose is likely to be related to a requirement for seeking food in variable migratory habitats.

e) Initial characterization of the three-dimensional organization of goose liver tissues

We analyzed the inter-pseudo-chromosomal interaction pattern[57], compartments[58, 59], topologically associating domains (TADs)[60], and promoter-enhancer interactions (PEI)[61] of the goose liver tissue. The matrix resolution of our Hi-C experiment reached ~2 Kb (defined as the smallest locus size such that 80% of loci have at least 1,000 contacts) (Figure S6), which was adequate for subsequent analyses of the chromatin architecture. Our results showed that the whole inter-pseudo-chromosomal interaction pattern was distinguished by two clusters, that is, short pseudo-chromosomes and longer pseudo-chromosomes, which suggests that goose pseudo-chromosomes tend to interact with one another on the basis of size (**Figure 2**). As for the identification of A and B compartments, which represent relatively active and inactive chromatin states, respectively, the number of protein-coding genes (PCGs) in each 100 Kb bin with at least 50 % percentage overlapped with a gene was counted. The number of PCGs was

significantly correlated with PC1 values (R = 0.39, *p* = 2.2×10^{-16}; **Figure S7**), and the transcripts per kilobase millions (TPMs) of PCGs located in A compartments were consistently higher than PCGs in B compartments in three liver tissues (*p* = 2.2×10^{-16}; **Figure S8, Table S9**). We identified 734 TADs across the goose assembly, accounting for 80% of the genome (**Figure S9, Table S10**). The mean and median sizes of the TADs were 1.21 Mb and 1.00 Mb, respectively. We also observed that the TSSs of PCGs were enriched in TAD-boundary regions (**Figure S10**). After filtering for interaction distances lower than 20 Kb, we identified 13,017 PEIs (**Table S11**) and found that gene expression levels positively correlated with the number of its associated enhancers in all three liver tissues (**Figure S11**). This is suggestive of additive effects of enhancers on target-gene transcription levels.

Availability of supporting data

The chromosome-level goose genome assembly sequence is available at National Center for Biotechnology Information (NCBI) GenBank through the accession number WTSS00000000; The high-quality Hi-C data are available through the NCBI Sequence Read Archive (SRA) database under accession number SRR10483522. The PacBio long-read sequencing data have been deposited in the NCBI SRA (SRR10483521). The high-quality Illumina short-read sequencing data are available through NCBI SRA accession number: SRR10483516, SRR10483517, SRR10483518 and SRR10483520. The transcriptome data are available through the NCBI SRR10483519.

List of abbreviations

(1) Anser anser：A. anser;

(2) Anser cygnoides：A. cygnoides;

(3) BUSCO: Benchmarking Universal Single-Copy Orthologs;

(4) CHMP1B: charged multivesicular body protein 1B;

(5) CEGMA: Core Eukaryotic Genes Mapping Approach software;

(6) TUCP: transcripts of uncertain coding potential;

(7) GCH1: GTP cyclohydrolase 1;

(8) Hi-C, Chromosome conformation capture;

(9) IVNS1ABP: influenza virus NS1A binding protein;

(10) LINEs: Long interspersed nuclear elements;

(11) LncRNAs: long noncoding RNAs;

(12) OGFOD2: 2-oxoglutarate and iron dependent oxygenase domain containing 2

(13) MDH257: malate dehydrogenase 2

(14) PCGs: protein coding genes

(15) PEI: promoter-enhancer interactions;

(16) PSGs: positively selected genes;

(17) SMRT: single-molecule real-time;

(18) TADs: topological associated domains;

(19) TPMs: transcripts per kilobase millions.

Ethics approval

All animal experiments were approved and reviewed by Animal Care and Use Committee Institutional of Sichuan Agricultural University (Approval No. DKY-B20121406) and the Ministry of Science and Technology of the People's Republic of China (Approval No. 2006–398).

Competing interests

The authors declare no competing interest.

Acknowledgments

This work was supported by grants from the National Key R & D Program of China (2018YFD0500403), the National Natural Science Foundation of China (U19A2036, 31872335, 31772576 and 31802044) and the China Postdoctoral Science Foundation (2018M643514).

Author contributions

Mingzhou Li, Guangliang Gao designed and supervised the project. Yan Li, Yu Lin, Qianzi Tang, Silu Hu performed bioinformatics analyses. Jiwen Wang, Yan Li and Yi Luo contributed to collect the samples. Mingzhou Li, Qigui Wang, Guangliang Gao, Yi Luo and Long Jin were involved in the data analyses and wrote the manuscript.

References

1. Shi XW, Wang JW, Zeng FT, et al. Mitochondrial DNA cleavage patterns distinguish independent origin of Chinese domestic geese and western domestic geese. Biochem Genet. 2006; 44(5-6) : 237-245.
2. Kozák J. Variations of geese under domestication. World's Poult Sci J. 2019; 75(2): 247-260.
3. Goluch-Koniuszy Z, Haraf G. Geese for slaughter and wild geese as a source of selected mineral elements in a diet. J Elementol. 2018; 23: 1343-1360.
4. Janan J, Tóth P, Hutas I, et al. Effects of dietary micronutrient supplementation on the reproductive traits of laying geese. Acta Fytotech Zootech. 2015; 18(1) : 6-9.
5. Zhang Y, Sha Z, Guan F, et al. Impacts of geese on weed communities in corn production systems and associated economic benefits. Biol Control. 2016. 99: 47-52.
6. Wang G, Jin L, Li Y, et al. Transcriptomic analysis between Normal and high-intake feeding geese provides insight into adipose deposition and susceptibility to fatty liver in migratory birds. BMC genomics. 2019; 20(1): 372.
7. Honka J, Heino M, Kvist L, et al. Over a thousand years of evolutionary history of domestic geese from Russian archaeological sites, analysed using ancient DNA. Genes. 2018; 9(7): 367.

8. Lu L, Chen, Y, Wang Z, et al. The goose genome sequence leads to insights into the evolution of waterfowl and susceptibility to fatty liver. Genome Biol. 2015; 16(1): 89.

9. Gao G, Zhao X, Li Q, et al. Genome and metagenome analyses reveal adaptive evolution of the host and interaction with the gut microbiota in the goose. Sci Rep. 2016; 6: 32961.

10. Schadt E, Turner S, Kasarskis A. A window into third-generation sequencing. Hum Mol Genet. 2010; 19(R2): R227-R240.

11. Dudchenko O, Batra SS, Omer AD, et al. *De novo* assembly of the *Aedes aegypti* genome using Hi-C yields chromosome-length scaffolds. Science. 2017; 356(6333): 92-95.

12. Pendleton M, Sebra R, Pang AWC, et al. Assembly and diploid architecture of an individual human genome via single-molecule technologies. Nat Methods. 2015; 12(8): 780–786.

13. Bickhart DM, Rosen BD, Koren S, et al. Single-molecule sequencing and chromatin conformation capture enable *de novo* reference assembly of the domestic goat genome. Nat Genet. 2017; 49(4): 643.

14. Liu Q, Wang X, Xiao Y, et al. Sequencing of the black rockfish chromosomal genome provides insight into sperm storage in the female ovary. DNA Research, 2019. 26(6):453–464,

15. Mascher M, Gundlach H, Himmelbach A, et al. A chromosome conformation capture ordered sequence of the barley genome. Nature. 2017; 544(7651): 427

16. Chin CS, Peluso P, Sedlazeck FJ et al. Phased diploid genome assembly with single molecule real-time sequencing. Nat Methods. 2016;13:1050.

17. Boetzer M, Pirovano W. SSPACE-LongRead: scaffolding bacterial draft genomes using long read sequence information. BMC Bioinf. 2014; 15(1): 211.

18. English AC, Richards S, Han Y, et al. Mind the gap: upgrading genomes with Pacific Biosciences

RS long-read sequencing technology. PLoS One. 2012; 7(11): e47768.

19. Walker BJ, Abeel T, Shea T, et al. Pilon: an integrated tool for comprehensive microbial variant detection and genome assembly improvement. Plos One. 2014; 9(11): e112963.

20. Burton JN, Adey A, Patwardhan RP, et al. Chromosome-scale scaffolding of *de novo* genome assemblies based on chromatin interactions. Nat Biotechnol. 2013; 31(12): 1119–1125.

21. Jun X, Tianxing L, Qing C, et al. Karyotypes of Zhedong White Goose and Siji Goose. China Poultry. 2007; 21(9): 27-29.

22. Benson G. Tandem repeats finder: a program to analyze DNA sequences. Nucleic Acids Res. 1999; 27(2): 573–580.

23. RepeatMolder software. http://www.repeatmasker.org/RepeatModeler/.

24. Price AL , Jones NC, Pevzner PA. De novo identification of repeat families in large genomes. Bioinformatics2005;21(suppl 1):i351–8.

25. Price AL, Jones NC, Pevzner PA. De novo identification of repeat families in large genomes. Bioinformatics. 2005;21:i351–8. Bao W, Kojima KK, Kohany O. Repbase Update, a database of repetitive elements in eukaryotic genomes. Mobile DNA. 2015; 6(1):11.

26. Maja TG, Nansheng C. Using RepeatMasker to identify repetitive elements in genomic sequences. Curr Protoc Bioinf. 2009; 25(1): 4.10.11–14.10.14.

27. Allred DB, Cheng A, Sarikaya M, et al. . Three-dimensional architecture of inorganic nanoarrays electrodeposited through a surface-layer protein mask. Nano Lett. 2008;8(5):1434–8.

28. Burge C, Karlin S. Prediction of complete gene structures in human genomic DNA. J Mol Biol. 1997; 268(1): 78–94.

29. Blanco E, Parra G, Guigó R. Using geneid to identify genes. Curr Protoc Bioinf. 2007; 18(1): 4.3.1-

4.3.28.

30. Stanke M, Steinkamp R, Waack S. AUGUSTUS: a web server for gene finding in eukaryotes. Nucleic Acids Res. 2004; 32(suppl_2): W309–W312.

31. Gertz EM, Yu YK. Agarwala, R., Schäffer, A. A. & Altschul, S. F. Composition-based statistics and translated nucleotide searches: improving the TBLASTN module of BLAST. BMC Biol. 2006; 4(1): 41.

32. Birney E, Clamp M, Durbin R. Gene Wise and Genomewise. Genome Res. 2004; 14(5): 988–995.

33. Grabherr MG, Haas BJ, Yassour M, et al. Full-length transcriptome assembly from RNA-Seq data without a reference genome. Nat Biotechnol. 2011; 29(7): 644–652.

34. Trapnell C, Pachter L, Salzberg SL. TopHat: discovering splice junctions with RNA-Seq. Bioinformatics. 2009; 25(9): 1105–1111.

35. Haas BJ, Salzberg SL, Zhu W, et al. Automated eukaryotic gene structure annotation using EVidenceModeler and the Program to Assemble Spliced Alignments. Genome Biol. 2008; 9(1): R7.

36. Dobin A, Davis CA, Schlesinger F, et al. STAR: ultrafast universal RNA-seq aligner. Bioinformatics. 2013; 29(1): 15–21.

37. Trapnell C, Williams BA, Pertea G, et al. Transcript assembly and quantification by RNA-Seq reveals unannotated transcripts and isoform switching during cell differentiation. Nat Biotechnol. 2010; 28(5): 511–515.

38. Niknafs YS, Pandian B, Iyer HK, et al. TACO produces robust multisample transcriptome assemblies from RNA-seq. Nat Methods. 2017; 14(1): 68.

39. Kang YJ, Yang DC, Kong L, et al. CPC2: a fast and accurate coding potential calculator based on sequence intrinsic features. Nucleic Acids Res. 2017; 45(W1): W12-W16.

40. Finn RD, Bateman A, Clements J, et al. Pfam: the protein families database. Nucleic Acids Res. 2014; 42(D1): D222–D230.

41. Bateman A, Coin L, Durbin R, et al. The Pfam protein families database. Nucleic Acids Res. 2004; 32 (suppl_1): D138–D141.

42. Zhang G, Li C, Li Q, et al. Comparative genomics reveals insights into avian genome evolution and adaptation. Science. 2014; 346(6215): 1311-1320.

43. Ottenburghs J, Megens HJ, Kraus RH, et al. A history of hybrids? Genomic patterns of introgression in the True Geese. BMC Evol Biol. 2017; 17(1): 201.

44. Fischer S, Brunk BP, Chen F, et al. Using OrthoMCL to assign proteins to OrthoMCL- DB groups or to cluster proteomes into new ortholog groups. Curr Protoc Bioinf. 2011; 35(1): 6-12.

45. Stamatakis A. RAxML version 8: a tool for phylogenetic analysis and post-analysis of large phylogenies. Bioinformatics. 2014; 30(9): 1312–1313.

46. Bie T, Cristianini N, Demuth J. CAFE: a computational tool for the study of gene family evolution. Bioinformatics. 2006; 22(10): 1269-1271.

47. Józefiak DA, Rutkowski A, Martin SA. Carbohydrate fermentation in the avian ceca: a review. Anim Feed Sci Technol. 2004; 113(1-4): 1-15.

48. Watanabe, YY. Flight mode affects allometry of migration range in birds. Ecol Lett. 2016; 19(8): 907-914.

49. Yoshino H, Nishioka K, Li Y, et al. GCH1 mutations in dopa-responsive dystonia and Parkinson's disease[J]. J Neuro, 2018, 265(8): 1860-1870.

50. Gu Y, Lu K, Yang G, et al. Mutation spectrum of six genes in Chinese phenylketonuria patients obtained through next-generation sequencing[J]. PLoS One, 2014, 9(4): e94100.

51. He Y B, Duojizhuoma C Y, Cai-juan D B, et al. GCH1 plays a role in the high-altitude adaptation of Tibetans. Zool Res. 2017; 38(3): 155–162.

52. Verma S, De Jesus P, Chanda S K, et al. SNW1, a novel transcriptional regulator of the NF-κB pathway. Mol Cell Biol. 2019; 39(3): e00415-18.

53. Tolde O, Folk P. Stress-induced expression of p53 target genes is insensitive to SNW1/SKIP downregulation[J]. Cell Mol Biol Lett, 2011, 16(3): 373-384.

54. Wu M Y, Ramel M C, Howell M, et al. SNW1 is a critical regulator of spatial BMP activity, neural plate border formation, and neural crest specification in vertebrate embryos[J]. PLoS Biol, 2011, 9(2): e1000593.

55. Huang Y H, Klingbeil O, He X Y, et al. POU2F3 is a master regulator of a tuft cell-like variant of small cell lung cancer. Gene Dev. 2018; 32(13-14): 915-928.

56. Matsumoto I, Ohmoto M, Narukawa M, et al. Skn-1a (Pou2f3) specifies taste receptor cell lineage[J]. Nat Neurosci, 2011, 14(6): 685.

57. Battulin N, Fishman VS, Mazur AM, et al. Comparison of the three-dimensional organization of sperm and fibroblast genomes using the Hi-C approach. Genome Biol. 2016; 17(1): 6.

58. Lieberman-Aiden E, van Berkum NL, Williams L, et al. Comprehensive mapping of long-range interactions reveals folding principles of the human genome. Science. 2009; 326(5950): 289-293.

59. Rowley MJ, Nichols MH, Lyu X, Ando-Kuri M, et al. Evolutionarily Conserved Principles Predict 3D Chromatin Organization. Mol Cell. 2017; 67(5): 837-852.

60. Dixon JR, Selvaraj S, Yue F, et al. Topological domains in mammalian genomes identified by analysis of chromatin interactions. Nature. 2012; 485(7389): 376-380.

61. Ron G, Globerson Y, Moran D and Kaplan T. Promoter-enhancer interactions identified from Hi-C

data using probabilistic models and hierarchical topological domains. Nat Commun. 2017; 8(1): 2237.

Click here to access/download;Table;Table 1.docx

Table1 Summary of sequencing data for goose genome assembly.

Pair-end libraries	Insert size (bp)	Total data (Gb)	Read length (bp)	Sequence coverage (×)
**Illumina reads**	350	181.52	150	141.81
**Pacbio reads**	20,000	84.31		65.86
**Hi-C**	350	149.70	150	116.95
**Total**		415.53		324.63

Click here to access/download;Table;Table 2.docx

Table2 Comparison of quality metrics of this study and the previous goose genome assemblies.

**Genomic features**	**This study**	**Lu *et al.*[a]**	**Gao *et al.*[b]**
Estimation of genome size (bp)	1,277,099,016	1,208,661,181	1,198,802,839
Total length of assembled contigs (bp)	1,113,842,245	1,086,838,604	1,100,859,441
Total size of assembled scaffolds (bp)	1,113,913,845	1,122,178,121	1,130,663,797
Number of contigs (>2kb)	2,771	60,979	53,336
Number of scaffolds （>2kb）	2,055	1,050	1,837
Contigs N50 (bp)	1,849,874	27,602	35,032
Scaffolds N50 (bp)	33,116,532	5,202,740	5,103,766
Longest contig (bp)	10,766,871	201,281	399,111
Longest scaffold (bp)	70,896,740	24,051,356	20,207,557
GC content (%)	42.15	38.00	41.68
Number of gene model	17,568	16,150	16,288
Repetitive regions percentage of genome (%)	8.67	6.33	6.90

[a] From the ref. 8. [b] From the ref. 9.

Click here to access/download;Table;Table 3.docx

Table 3 A comparative summary of predicted genes within each goose genome assembly.

Property	This study	Lu *et al.*[a]	Gao *et al.*[b]
Total PCG length (bp)	326,863,440	439,289,059	500,923,091
PCG number	17,568	16,150	16,288
PCG percentage of genome (%)	29.34	39.25	44.31
Total exons number	152,392	158,713	167,532
Average exons per gene	8.67	10.92	10.29
Total exons length (bp)	26,883,354	25,763,242	26,157,477
Exons percentage of genome (%)	2.41	2.31	2.31
Average exons length (bp)	176.41	162.33	156.13
Average introns length (bp)	2224.97	2867.48	3139.07

[a] From the ref. 8. [b] From the ref. 9.

Figure 1 Comparison of the distribution and coverage of the scaffolds for the assembly with previous goose genome assemblies.

Click here to access/download;Figure;Figure 1u.tif

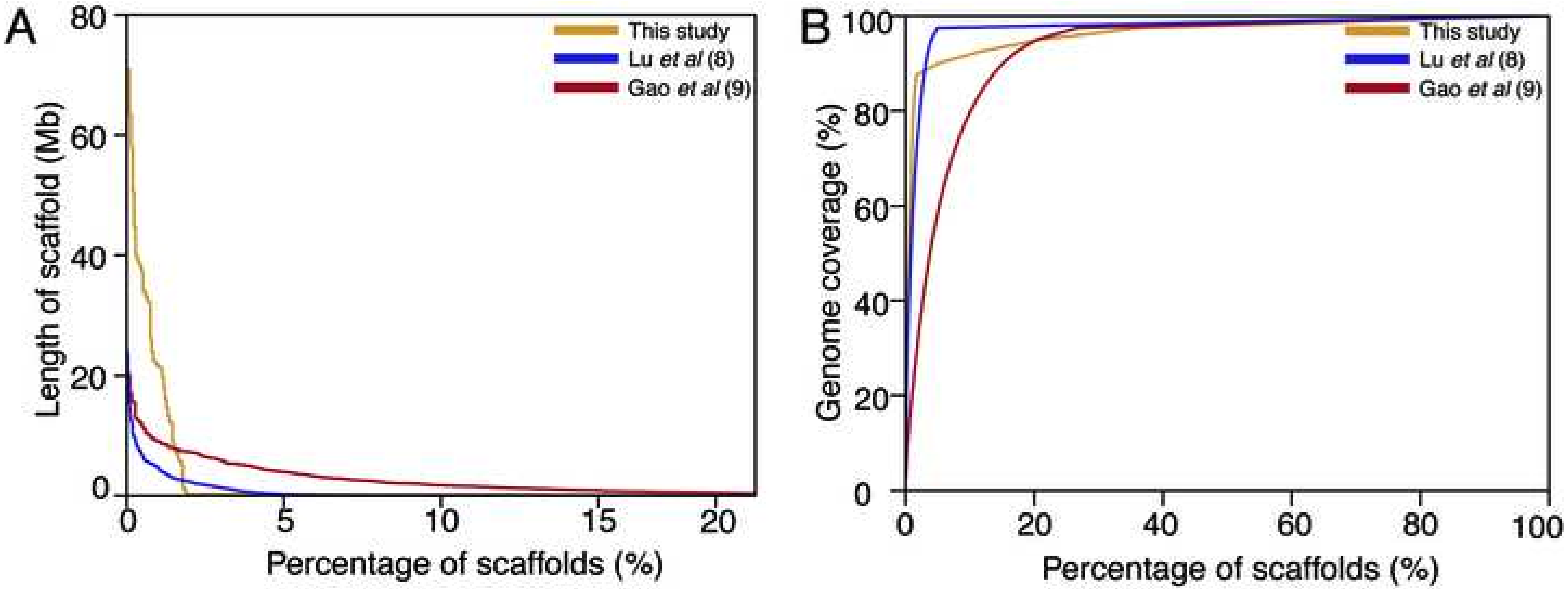

Figure 2 Dendrogram of inter-pseudo-chromosome interaction patterns generated by the average linkage algorithm.

Click here to access/download;Figure;Figure 2u.tiff

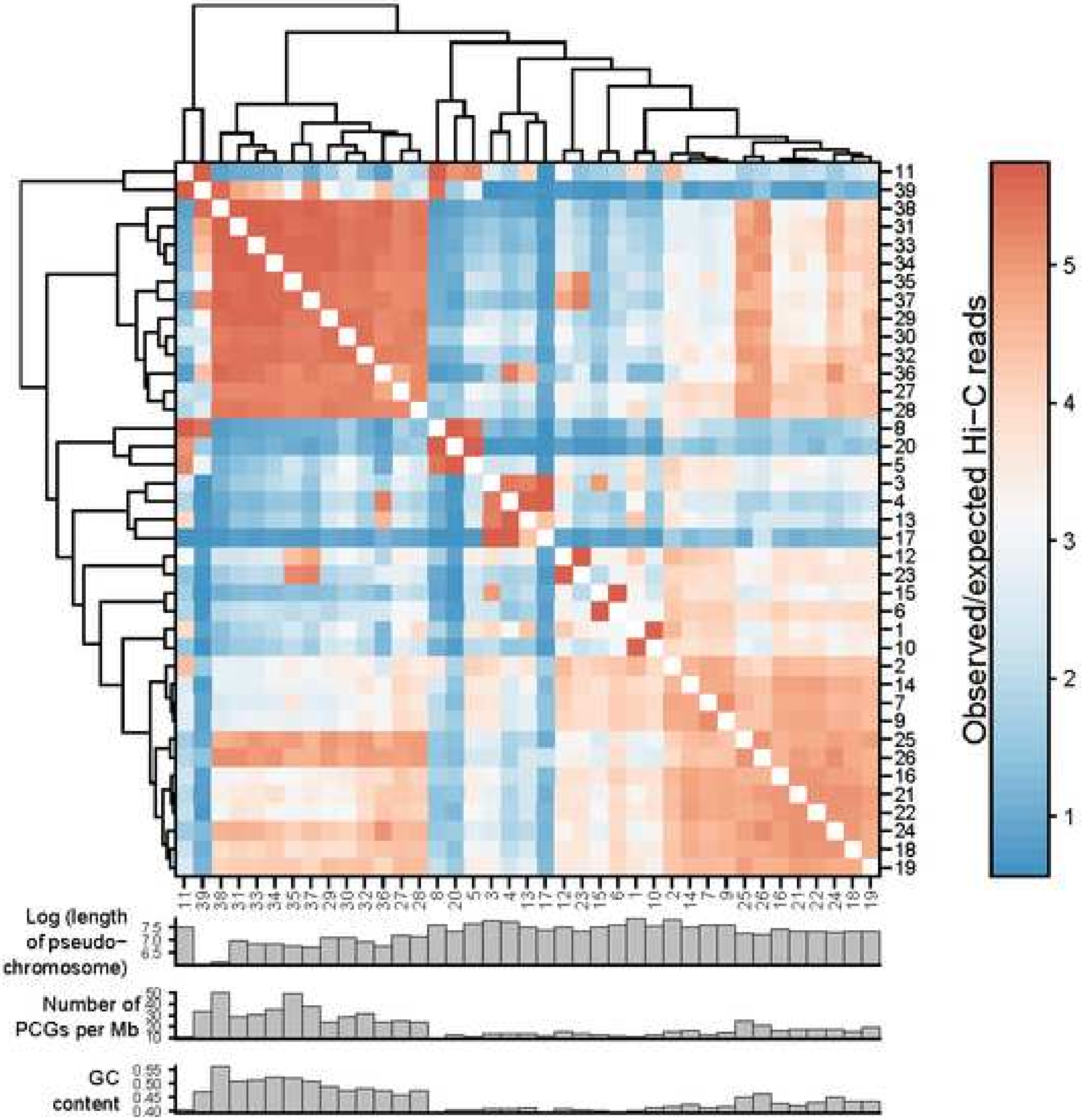

Click here to access/download
Supplementary Material
Supplymental_materials.docx.docx

Table S1 Summary of the Pacbio initial assembly and Hi-C reads mapping used for goose genome assembly process.

Click here to access/download
Supplementary Material
Table S1.xls

Table S2 Summary of the length of pseudo-chromosomes in goose genome.

Click here to access/download
Supplementary Material
Table S2.xls

Table S3 A comparative summary of assembled repeat content between this study and previous studies.

Click here to access/download
Supplementary Material
Table S3.xls

Table S4 Comparison of the mapping rates of the wild goose resequencing data between our goose genome and two previous

Click here to access/download
Supplementary Material
Table S4.xls

Table S5 Gene ontology (GO) enrichment analysis for the lineage-specific genes annotated in goose genome.

Click here to access/download
Supplementary Material
Table S5.xls

Table S6 Functional gene categories enriched for the goose genome-specific expansion gene families.

Click here to access/download
Supplementary Material
Table S6.xls

Table S7 Functional gene categories enriched for the contraction of gene families in goose genome.

Click here to access/download
Supplementary Material
Table S7.xls

Table S8 Positively selected genes (PSGs) identified in the goose genome.

Click here to access/download
Supplementary Material
Table S8.xlsx

Table S9 The PC1 values (100 Kb) through Principal Component Analysis (PCA) and A-B index values (25 Kb).

Click here to access/download
Supplementary Material
Table S9.xlsx

Table S10 TAD in genome coordinates of our goose genome by using method of DI values.

Click here to access/download
Supplementary Material
Table S10.xlsx

Table S11 Detailed information of promoter-enhancer interactions (PEIs) identified in goose genome.

Click here to access/download
Supplementary Material
Table S11.xlsx

Figure S1 A picture of a female adult goose used for genome sequencing.

Click here to access/download
Supplementary Material
Figure s1u.tif

Figure S2 The Hi-C interaction contact heatmap of goose pseudochromosome genome assembly (bin size is 1Mb).

Click here to access/download
Supplementary Material
Figure S2u.jpg

Figure S3 The shared homologous gene families across the six species (Chicken, Goose, Human, Mouse, Pig, Zebra finch).

Click here to access/download
Supplementary Material
Figure S3u.tiff

Figure S4 The distribution of gene density in the goose genome.
Number of PCGs in each 1Mb bins was counted.

Click here to access/download
Supplementary Material
Figure s4u.tif

Figure S5 Divergence of time and the expansion, contraction gene families in the seventeen species (Ostrich, Duck, Goose, Chicken,

Click here to access/download
Supplementary Material
Figure S5.jpg

Figure S6 Resolution evaluation showing that the Hi-C data attained 2 Kb.

Click here to access/download
Supplementary Material
Figure S6.tif

Figure S7 Vioplot of PC1 values in 100 Kb bins with various number of PCGs. PC1 value indicates the chromatin activity.

Click here to access/download
Supplementary Material
Figure S7u.tif

Figure S8 TPMs of PCGs located in A compartments were consistently higher than PCGs in B compartments both at 25 Kb

Click here to access/download
Supplementary Material
Figure S8u.tif

Figure S9 TAD distribution across the goose genome assembly.

Click here to access/download
Supplementary Material
Figure S9.tif

Figure S10 TSSs of PCGs were enriched in TAD boundary regions.

Click here to access/download
Supplementary Material
Figure S10u.tif

Figure S11 Gene expression levels positively correlated with the number of its associated enhancers in all three liver tissues,

Click here to access/download
Supplementary Material
Figure S11u.tif

GigaScience

maryann@gigasciencejournal.com

Dear Mary Ann Tuli

Please find enclosed our revised manuscript, "PacBio assembly with Hi-C mapping generates an improved, chromosome-level goose genome (GIGA-D-20-00133)", which we would like to resubmit to ***GigaScience*.** We sincerely appreciate the very thoughtful and constructive comments from the two anonymous referees. We have adhered to the referees' comments and believe that we have adequately addressed all their questions and concerns. We have made all the changes in the revised version of the manuscript, and our point-by-point responses to the reviewer's comments are given below. We trust that the revised manuscript now meets the standards required for publication in ***GigaScience***.

We look forward to hearing a positive response from you.

Best regards

Mingzhou Li

Ph.D/Professor/

Address: Institute of Animal Genetics and Breeding, College of Animal Science and Technology, Sichuan Agricultural University, Chengdu 611130, China.

E-mail: mingzhou.li@sicau.edu.cn

Detailed responses to Reviewers

Below, all critiques and suggestions provided by the reviewers are cited in gray italics; our responses are in black. In red are descriptions within the responses that indicate changes in the manuscript. Moreover, all revisions in the manuscript are marked in red.

Reviewer 1

This paper reports on the assembly and annotation of the Tianfu goose genome, a female hybrid of A. anser x A. cygnoides. This assembly is a significant improvement on earlier genomes, which were based on short read technologies. This assembly is a hybrid of three technologies: short reads, long reads and HiC maps.

Comment 1:

MAJOR POINTS

1. The assignment of 39 chromosomes to Hi-C scaffolds is very tentative and needs to be validated. For larger scaffold you could establish homology e.g. with chicken chromosomes, which have extensive FISH/cytogenetic data at least for the macrochromosomes. The smaller scaffolds in the HiC analysis could be parts of larger chromosomes - the HiC map suggests some mis-joins. Also in other genome projects very GC-rich, repeat-rich chromosomes (such as microchromosomes) are difficult if not impossible to sequence, and are missing from the assembly. So 39 pseudo-chromosomes are found but these do not equate to the 39 physical chromosomes. This affects conclusions on chromosome number, genome completeness, gene density distribution, distribution of TADs, etc. As a reference goose genome these points need to be addressed.

Response 1：

Thank you for this valuable suggestion. We recognize the importance of a reference genome that comprises accurate, physical chromosomes for future genetic and genomic studies on geese. In this study, we generated a 1.11 Gb goose genome with contig and scaffold N50 values of 1.85 Mb and 33.12 Mb, respectively (Table 1). Our assembly contains 39 pseudo-chromosomes (2n = 78), which account for 88.36% of the goose genome; it is a draft goose genome assembly rather than a complete assembly of 39 physical chromosomes. As far as we know, this genome is comparable with other chromosome-level avian genome assemblies (Table 1).

We regret that our original description implied a complete genome assembly of 39 chromosomes. We have corrected this point by stating throughout the manuscript that our assembly is a chromosome-level goose genome assembly comprising 39 pseudo-chromosomes.

Table 1: Comparison of quality metrics of goose, chicken, duck, turkey, and zebra finch genome assemblies.

	Goose (This study)	Chicken GCF_000002315.6	Duck GCF_003850225.1	Turkey GCF_000146605.3	zebra finch GCF_003957565.1
Assembly level	pseudo-chromosome	chromosome	chromosome	chromosome	chromosome
Total sequence length (bp)	1,113,842,245	1,065,365,434	1,126,176,092	1,115,474,681	1,058,012,133
Number of scaffolds	2,123	525	2,150	187,695	135
Scaffold N50 (bp)	33,116,532	20,785,086	76,129,154	3,898,092	70,430,603
Number of contigs	2,771	1,403	73,853	250,220	444
Contig N50 (bp)	1,849,874	17,655,422	36,805	27,076	11,998,827

OTHER POINTS

Comment 1-1:

1. This is a chromosome-level assembly make this clear in the text.

Response 1-1:

In accordance with Reviewer 1's suggestion, we have clarified that it is a chromosome-level assembly throughout the manuscript.

Comment 1-2:

2. The hybrid approach used here is good but this is a rapidly evolving field, and is already superseded by technology (Pacbio HiFi now so polishing using short reads not needed) and software (e.g. Lachesis no longer supported).

Response 1-2:

Thank you for this valuable insight. Certainly, *de novo* whole-genome assembly approaches change over time, and algorithms adapt in line with evolving sequencing technologies. This allows researchers to generate more continuous, complete, and accurate genome assemblies. To facilitate genomic, genetic, and breeding studies on the goose, we report here an improved, chromosome-level goose genome for the scientific community. We expect that our current goose assembly and annotation will be helpful for researchers in different study fields. Furthermore, we remain committed to assembling a complete and accurate goose genome sequence in the future. For this

purpose, we plan to adopt the Pacbio HiFi and Pacbio isoseq technologies, and combine them with extensive FISH and cytogenetic experiments, and genetic map data.

Regarding the three software packages, LACHESIS, SALSA2, and 3D-DNA, each have advantages and limitations for *de novo* genome assembly. Namely, (1) while we employed the LACHESIS software to combine shotgun fragments and short jump mate-pair sequences with Hi-C data (to generate chromosome-scale *de novo* genome assemblies), LACHESIS has limitations when assembling polyploid genomes [1]. (2) The SALSA2 algorithm does not require that the number of chromosomes are set in advance, which improves the accuracy of scaffolds to a certain extent; however, this algorithm can introduce many clustering/sorting/orientation errors, and few parameters can be adjusted during operation [2]. (3) 3D-DNA corrects errors in the input assembly and then iteratively orients and orders contigs into a single mega-scaffold. This mega-scaffold is then broken, and chromosomal ends are identified based on a Hi-C contact map. A drawback is that the error correction function in this software has not been well applied; in the case of simulated data, the assembly error rate of 3D-DNA is 2–4 times that of SALSA2 [2].

To choose an appropriate software for our genome assembly, we randomly selected a subset of our Hi-C data and performed *de novo* genome assembly using SALSA2, 3D-DNA, and LACHESIS. As the quality metrics of the LACHESIS genome assemblies were the best, we performed the subsequent studies in this paper with the LACHESIS software.

Comment 1-3:

3. The phrase "high-quality" is used throughout the text but not defined - so please define. It is more likely that sequence data is generated (provide QC data on quality) and then software is used to filter out poor data, to leave high-quality data for assembly.

Response 1-3

Thank you for this useful insight. In our manuscript, the phrase "high-quality" refers to data that was filtered from three sequencing platforms and used for the genome assembly. For the short reads, we employed a Perl script written by our lab to filter the data from the Illumina platform. As a result, the Q20 and Q30 values of the whole genome sequencing data from the Illumina platform were greater than 96.44 % and

93.25 %, respectively (Table 2). The Q20 and Q30 values of the Hi-C data used in our study were 97.86 % and 91.84 %, respectively (Table 2). These results suggest that the data used in our genome assembly were "high-quality".

Table 2: Summary of quality control data.

Type of library	SRA number	Raw Base (bp)	Effective Rate (%)	Clean base (bp)	Error rate (%)	Q20 (%)	Q30 (%)	GC Content (%)
WGS	SRR10483520	45084213600	99.90	45036954900	0.03	96.56	93.48	44.01
WGS	SRR10483518	44824006500	99.90	44777152500	0.03	96.44	93.25	43.96
WGS	SRR10483517	45752767800	99.89	45702384600	0.03	96.59	93.56	44.05
WGS	SRR10483516	46056473400	99.89	46005792900	0.03	96.72	93.78	44.08
Hi-C	SRR10483522	152176227800	98.12	149315314800	0.04	97.86	91.84	45.12

Comment 1-4:

4. For all software, please provide versions and source.

Response 1-4:

Following Reviewer 1's valuable suggestion, we have added the below descriptions to the supplemental materials (see lines 2–63).

Goose genome assembly, annotation and the spatial organization of chromatin in liver tissues analysis by the following software

Goose genome were de novo assembled by the following software:

(1) FALCON: version 3.1, parameters: length_cutoff = 5000 length_cutoff_pr = 4500;

(2) pbsmrtpipe: version smrtlink_5.0.1, default parameters;

(3) SSPACE-LongRead: version 1-1, default parameters;

(4) PBjelly: version PBSuite_15.8.24, parameters: blasr: -minMatch 8 -minPctIdentity 75 -bestn 1 -nproc 13 -noSplitSubreads;

(5) pilon: version pilon-1.18, parameters: -Xmx400G --diploid --threads 30;

(6) Lachesis: version-201701, parameters: RE_SITE_SEQ = GATC, CLUSTER_N = 39, CLUSTER_MIN_RE_SITES = 600, CLUSTER_MAX_LINK_DENSITY = 3, CLUSTER_NONINFORMATIVE_RATIO = 0.

(7) kallisto: version 0.44.0, parameters: -i -o --bias --rf-stranded.

Goose genome were annotated followed the software:

(1) GCE: version1.0.0, parameters: -H 1;

(2) SOAPdenovo: version2, k-mer size of 59;

(3) GAPcloser: version1.12, parameters: -l 150 -p 31;

(4) SSPACE: version3.0, default parameters;

(5) RepeatMasker: Repeat Masker-open-4-0-6, parameters: -a -nolow -no_is -norna -parallel 1;

(6) RepeatModeler: RepeatModeler-open-1.0.11, parameters: -database genome -engine ncbi -pa 15;

(7) Tandem Repeats Finder: TRF-407b, parameters: 2 7 7 80 10 50 2000 -d -h;

(8) TBLASTN: blast-2.2.26, parameters: -e 1e-05 -F T -m 8;

(9) GeneWise: version2.4.1, parameters: -tfor/-trev -genesf -gff;

(10) Augustus: version3.2.3, param- eters: –uniqueGeneId = true–noInFrameStop = true–gff3 = on–genemodel = complete–strand = both;

(11) GlimmerHMM: version3.0.1, parameters: -g -f;

(12) SNAP: snap-2013-11-29, default parameters;

(13) Trinity: trinityrnaseq-2.1.1, parameters: –seqType fq-CPU 20–max_memory 200G–normalize_reads–full_cleanup– min_glue 2–min_kmer_cov 2–KMER_SIZE 25;

(14) PASA: PASA_r20140417, default parameters;

(15) InterPro: version29.0, perl-based version4.8, default parameters;

(16) tRNAscan-SE: tRNAscan-SE-1.3.1, default param- eters;

(17) INFERNAL: version1.1rc4 (June 2013);

(18) BLASTp: blast-2.2.26, parameters: -p blastn -e 1e-10 -v 10000 -b 10000;

(19) EVM: VidenceModeler-1.1.1, parameters: –segment- Size 200000–overlapSize 20000;

(20) Tophat: tophat-2.0.13, parameters: -p 6–max-intron-length 500000 -m 2– library-type fr-unstranded;

(21) Cufflinks: cufflinks-2.1.1, parameters: -I 500000 -p 1–library-type fr-unstranded -L CUFF;

(22) BUSCO: version3.0.2, OrthoDBv9_vertebrata;

(23) BWA: bwa-0.7.8, parameters: mem -k 32 -w 10 -B 3 -O 11 -E 4 -t 10;

(24) SAMtools: samtools-0.1.19, parameters: mpileup mpileup -m 2 -u;

(25) RAxML: version 8.0.19, default parameters;

(26) CAFÉ: Version 1.6, default parameters;

(27) BLASTP: Version 2.2.26, default parameters;

(28) PAML: Version 14.7, default parameters;

LncRNA and TUCP were annotated followed the software:

(1) STAR: version 2.6.0c, default parameters;

(2) Cufflinks: version 2.2.1, default parameters;

(3) TACO: version 0.7.3, parameters: --filter-min-expr 0.1 --isoform-frac 0.1 --path-kmax 20 --max-paths 20 --filter-min-length 250 --gtf-expr-attr FPKM;

(4) taco_refcomp: part of TACO in version 0.7.3, parameters: -o -r -t

(5) CPC2: version beta of CPC2, default parameters;

(6) transeq: parts of EMBOSS in version 6.6.0, parameters: -sequence -outseq -frame 6 -clean;

(7) kallisto: version 0.44.0, parameters: -i -o --bias --rf-stranded.

Hi-C data analysis by the following software:

(1) Juicer: version 1.8.9, parameters: -C 8000000 -s MboI -p goose.chromosome.sizes -z goose.fa -y goose.MboI.fragment.txt -n 10G;

(2) Hi-C Domain Caller, pipeline to call domains from Hi-C experiments: http://chromosome.sdsc.edu/mouse/hi-c/download.html;

(3) PSYCHIC: parameters, res: 25000, win: 2000000, chrname: chr*, chrsize: chr*.size, output_prefix: goose.chr*.25000, output_dir: output_directory, input_matrix: goose.chr*.25000.normalized.matrix, gene_file: goose.gene.psychic.bed, skip_hierarchy: FALSE.

Comment 1-5:

5. LINE 84: k-mer distribution analysis used to estimate genome size - provide reference, software, method - also mention other QC estimates (repeats, polyploidy etc).

Response 1-5:

Thank you for this useful comment. To estimate genome size, repeat regions, heterozygosity, and polyploidy prior to assembling the goose genome, we employed survey software based on K-mer (k = 17) frequency distributions. This predicted the genome size, repeat ratio, and heterozygosity to be 1277.1 Mb, 39.8%, and 0.4%,

respectively. In the K-mer analysis, the goose data demonstrated a distribution typical of a diploid genome (Figure 1), showing only a single major peak—which could be used to estimate the genome size. In addition, the first minor peak represents the level of heterozygosity and the second minor peak represents the level of repeat sequence [3].

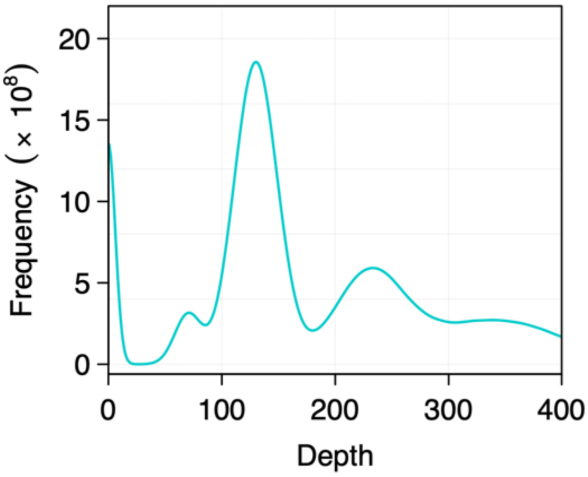

Figure 1. Frequency distribution of K-mer (17 bp).

Comment 1-6:

6. *LINE 91: Lachesis old software no longer supported - why not used SALSA2 or 3D-DNA?*

Response 1-6:

Thank you for raising this important point. Please see Response 1-2 for a discussion of this issue.

Comment 1-7:

7. Figure S1: Hi-C map suggests lots of mis-joins, have you checked and manually corrected?

Response 1-7:

Thank you for this valuable comment. In accordance with this point, we also identified mis-joins in the Hi-C map, which suggests that these regions of the genome might be repetitive, GC-rich, or contain structural variation. As mentioned above, we are committed to assembling a complete and accurate goose genome sequence, and in

future work aim to focus on these “mis-joins” using the latest technologies and corresponding assembly algorithms.

Comment 1-8:

8. LINES 109-111, again used the term "high-quality" for a mix of genomes, Human, Mouse, Chicken probably but duck, turkey and zebra finch are draft and not high-quality genomes.

Response 1-8:

Thank you for this insight. We have changed “high-quality" to “chromosome-level” in line 109. As described in Table 1, while the contig N50 values of zebra finch, duck, and turkey are 12.0 Mb, 36.80 Kb, and 27 Kb, respectively, these genomes were assembled into chromosome-level assemblies with the aid of other technologies, such as RH mapping and FISH (Table 1).

Comment 1-9:

9. LINES 114-117, pooled RNAseq used, so how can you quantify gene expression later in paper? Needs deconvolution of pooled samples - was this done? For annotation Pacbio isoseq would be better.

Response 1-9:

RNA-seq prediction is a commonly used method for improving genome annotation, correcting predicted gene structures, detecting new alternative splicing isoforms, and discovering new genes and transcripts. In this updated manuscript, we used data from the pooled RNA-seq analysis (abdominal fat, brain, duodenum, heart, liver, lung, muscular stomach, ovary, pancreas, pectoral muscle, and spleen) only for the annotation of the goose genome and not for gene expression quantification or for the spatial analysis of chromatin organization. Accordingly, we did not perform a deconvolution analysis of the pooled RNA-seq sample.

Nevertheless, we sincerely thank Reviewer 1 for this reminder on the correct usage of pooled RNA-seq data. We have now realized that our pooled RNA-seq data were inaccurately used to explore the spatial organization of chromatin in goose liver tissue.

To address this, we downloaded three RNA-seq datasets from liver tissues (Accession numbers: GSM3374538, GSM3374539, GSM3374540), which had been generated from the same goose strain used in our study [4]. We then quantified gene expression in these three samples and used these results to update all the content related to gene expression in our study, in terms of compartments and promoter-enhancer interactions. We have revised the main text and Figures S7–S11 and Figure 2 accordingly.

Regarding Pacbio, we also thank Reviewer 1 for raising this point. Certainly, the long reads from the Pacbio isoseq platform could better annotate complete transcripts in genomes. We aim to adopt this method, and other newly developed methods, when we update the quality of the goose genome assembly or annotation in future work.

Comment 1-10:

10. Prediction of lncRNAs from assembly of short read RNA-seq is known to be poor, so LINEs 121-124, where 3,287 lncRNAs are predicted needs to be taken with caution.

Response 1-10:

Thank you for this valuable suggestion. We agree that longer reads from PacBio and ONT offer advantages for resolving complex features in transcriptomes when compared to short read RNA-seq. For example, read length is the major advantage of Iso-Seq cDNA transcript sequencing and Oxford Nanopore direct RNA-seq, which can both capture entire transcripts.

Compared with mRNAs, which can be annotated by a combination of *ab initio* and homologous assembly approaches, lncRNAs are not conserved among species. lncRNAs can thus only be identified by transcript data, without the aid of homology. Long reads can be helpful for the identification and annotation of lncRNAs, and in future work, we will adopt this strategy. In this study, we identified lncRNAs by analyzing the transcript data from *short read RNA-seq* only, and we have clarified this point in lines 124–127 of the main text.

Comment 1-11:

11. LINE 160, goose and duck diverged 32 Mya, how does this estimate compare with other data sources?

Response 1-11:

Thank you for this important comment. In this study, we first downloaded the reported divergence times between each pair of species (e.g. chicken and turkey) from the TimeTree website (http://www.timetree.org/). These divergence times are estimated on the basis of single-copy gene families via a Bayesian algorithm called mcmctree, within the software "PAML (http://abacus.gene.ucl.ac.uk/software/paml.html)". We also used well-established divergence times to further adjust the estimated divergence times of other species and improve the accuracy of our results. The divergence times predicted in our study were consistent with two previous reports: 20.8 (12.9-32.7) Mya in Lu et al. [5]; and 30.0 (21.4-38.6) Mya in Gao et al. [6].

Comment 1-12:

12. sections (b-d) interesting predictions from phylogenetic analyses, but all speculation, there is no other data provided to back up these predictions.

Response 1-12:

Thank you for raising this point. The purpose of our article was to supply a valuable resource for future genetic and genomic studies on geese. Accordingly, we endeavored to explore the general characteristics of the goose genome by performing common general analyses—such as the expansion and contraction of gene families, and the identification of genes under positive selection. In the future, experiments on gene function will help to resolve the speculations and predictions we have presented here. We have revised the main text in lines 171, 191-192 and 198–211 to address this issue.

Comment 1-13:

13. LINE 192, PAML Codeml analysis is crude, and does not correct for multiple testing, with 17K genes tested there is a high false positive rate, was there any correction for multiple testing, if not please correct.

Response 1-13:

Thank you for this valuable comment. In our work, only single-copy genes (n = 2389) were used for the identification of genes under positive selection. We did not use all 17K genes. After we calculated the p-value for each of the candidate positively selected genes using PAML, we further adjusted the p-values (e.g. using the Bonferroni method, a method for multiple testing) to reduce false-positive results.

Comment 1-14:

14. LINE 202, the TAD analysis is restricted to liver tissue.

Response 1-14:

Thank you for raising this point. We explored the spatial organization of chromatin and gene expression in goose liver tissue only, with regard to topologically associating domains (TAD). TADs were largely invariable across tissues or species. We have clarified this point in lines 29, 213, 224, and 229.

Comment 1-15:

15. LINES 203-204, macs and mics form sub-domains in the nucleus. Figure 4 needs more explanation, poor figure.

Response 1-15:

We have replotted Figure 4 (named Figure 2 in revised manuscript) and increased the resolution of this figure. We have also added additional explanation and changed the figure legend as follows: "Dendrogram of inter-pseudo-chromosome interaction patterns generated by the average linkage algorithm. Heatmap shows the inter-pseudo-chromosome interaction probability, as generated by calculating the observed/expected contact frequencies for chromosome pair i, j. This is overlaid on a histogram showing pseudo-chromosome length, number of protein-coding genes, and GC percentage".

Comment 1-16:

16. LINE 205, define compartments A and B, how are these defined in Hi-C data?

Response 1-16:

Thank you for this valuable query. We have now added an explanation of the methods used for identifying compartments A and B, as well as the methods for how

the spatial organization of chromatin and gene expression were explored in the goose liver tissues (see lines 64–92 in the supplemental materials). These methods relate to inter-pseudo-chromosomal interaction patterns, topologically associating domains, promoter-enhancer interactions, and gene expression quantification.

Comment 1-17:

17. LINE 206, how were TSS (transcription start sites, not defined in the set of abbreviations, please add) defined? I assume based on the pooled short read RNA-seq data. If correct, this is a poor data set, since the assembly of transcripts based on short read data only defines the most 5′ RNA sequenced. So misses any internal TSS, does not correct for degraded RNA, etc.

Response 1-17:

Thank you for this comment. We agree that basing the definition of TSS sites on short read data would be inaccurate. We apologize for the ambiguous TSS-related description in our manuscript. We have changed the description in line 221 to 223: "the number of protein-coding genes (PCGs) in each 100-Kb bin with at least 50% percentage overlapped with a gene was counted. The number of PCGs was significantly correlated with PC1 values".

Comment 1-18:

18. LINE 213, gene expression levels based on pooled RNAseq data is a very poor dataset, should deconvolute or at least have a high-quality liver RNA set.

Response 1-18:

As described above (see Response 1-9), to address this issue we downloaded three additional RNA-seq datasets that were restricted to liver tissue (Accession numbers: GSM3374538, GSM3374539, GSM3374540). These datasets derive from the same goose strain as used in our study, and on the basis of a new analysis of these data we have updated all the sections of our manuscript related to gene expression. Specifically, we have changed the following description in lines 223–225: "the transcripts per kilobase millions (TPMs) of PCGs located in A compartments were significantly higher than those in B compartments", to: "the transcripts per kilobase millions (TPMs) of

PCGs located in A compartments were consistently higher than PCGs in B compartments in three liver tissues". We have also changed lines 229–230 from: "found that gene expression levels positively correlated with the number of PEIs", to: "found that gene expression levels positively correlated with the number of associated enhancers in all three liver tissues".

Reviewer 2

The manuscript describes a highly contiguous genome assembly of the goose genome and provides a significant improvement of the assembly of this bird. The results are described very clearly, and the data has been made publicly available. The analyses done are rather straightforward, and much more could have done with the interesting data generated in this study, which to me seems a missed opportunity.

The authors decide to sequence an F1 animal that is a cross between A. anser and A. cygnoides. I wonder why the authors did not use Illumina sequencing to sequence the genome of the two parents. This would have allowed the generation of two haplotype specific assemblies and the Comparison between the genomes of these two different sub-species. Also, no indication is given for the number of variants see in this bird, which would also have provided a good indication of the sequence divergence between these two sub-species. Finally, the realignment of the short-read Illumina sequences, provides a way to estimate the number of sequence errors still present in the final assembly (seen as homozygous SNPs and indels).

Response 2：

We apologize, it is apparent that our description of the Tianfu goose used for genome assembly in this study was not clear. Domesticated geese derive from the swan goose (*Anser cygnoides*) and the graylag goose (*Anser anser*). The Tianfu goose is a recognized breed that has originated from crosses between the domestic Landes goose (*A. cygnoides*) and the Sichuan white goose (*A. anser*), rather than the F1 animal crossed between *A. anser* and *A. cygnoides*. The Tianfu goose is a developed breed with many outstanding characteristics, such as excellent egg-laying performance, a fast growth rate, and strong adaptability. These characteristics are why we selected the Tianfu goose for this study.

Until now, a high-quality reference goose genome has not been available. To provide a valuable resource for future genetic and genomic studies on geese, and facilitate related research fields, our manuscript presents the first chromosome-level assembly of the goose genome. With reference to human and mouse research, in future studies we also aim to perform haplotype-resolved genome assemblies of F1 geese and parent animals, and compare differences between breeds.

Regarding the estimation of sequence errors, after we obtained our final goose assembly, we realigned the short read Illumina sequences with BWA software, and called SNPs and InDels using GATK software. As can be seen in Table 3, the proportions of homologous SNPs and InDels identified (which often reflect assembly errors) were extremely low, which indicates that our final assembly is of "high-quality".

Table 3: Homologous SNPs and InDels in the goose genome.

Category	Number	Proportion (%)
Homologous SNPs	23,324	0.0021
Homologous InDels	8,726	0.00078

OTHER POINTS

Comment 2-1:

Figure 1 and figure 2 are not very informative and I suggest moving these to the supplementary information

Response 2-1:

We agree with this suggestion from Reviewer 2. We have removed Figure 1 and Figure 4 to the supplementary figures, and have reordered the sequence of the corresponding supplementary figures.

Comment 2-2:

Line 89-90: The authors refer to table S1 in relation to the correction of sequencing errors. However, this table does not provide any information about sequencing errors.

Response 2-2:

We apologize for this inaccurate description. We have revised the main text to address this error, see lines 87–88.

Comment 2-3:

Line 90-91: The authors refer to table S2 and Fig S1. However, table S2 shows a summary of the pseudo chromosomes, not of the Hi-C scaffolds. Furthermore, in table S1 the authors show that there are 2123 Hi-C scaffolds. Please elaborate and clarify.

Response 2-3:

We regret the error in this description. Indeed, we state the length of the pseudo-chromosomes in the goose genome in Table S2, and present the Hi-C interaction contact heatmap of the pseudo-chromosomes in Figure S3. There are 2123 scaffolds in our goose genome assembly. This includes 68 scaffolds of 200bp to 2000bp, 2016 scaffolds of 2000bp to 350000bp, and 39 pseudo-chromosomes that are greater than 1Mb.

Comment 2-4:

Line 119-121: Again, the reference to the table/figure does not seem to match very well with the information in the text. I also suggest to add the number of PCG's to table 3. Also, does figure 2 only show the TSS for PCG or does it also include those for the lncRNAs.

Response 2-4:

Thank you for this valuable suggestion. Accordingly, we have added the number of PCGs to Table 3. In Figure 2, we show only the TSSs for PCGs. We have redrawn Figure 2.

Comment 2-5:

Line 128: I am confused by the comment that the current assembly has more scaffolds. Given that the assembly is improved with higher N50 values for the contigs and scaffolds, I would assume that the number would be smaller.

Response 2-5:

Thank you for raising this point. To display the quality of the genome assemblies, we analyzed the distribution of their scaffold lengths. In our goose genome, with the exception of the 39 pseudo-chromosomes, lengths of scaffolds are distributed from 2kb

to 350kb (Table 4). This indicates that our assembly contains 39 pseudo-chromosomes (longer than 1Mb) and 2016 scaffolds (of lengths ranging from 2kb to 350kb). To supply more information for researchers, we did not filter the 2kb–350kb scaffolds from our genome assembly data. As a result, we have reported more scaffolds in this study than were reported in two previous studies. However, as the 39 pseudo-chromosomes we assembled account for 88.36% of the genome (Table 4, Figure 2), this suggests that our genome assembly is an improvement on previous goose assemblies.

Table 4: Comparison of the distribution of scaffold lengths in three goose genomes.

	This study			Lu *et al*[a]			Gao *et al*[b]		
Scaffold length	Number	Percentage (%)	Coverage (%)	Number	Percentage (%)	Coverage (%)	Number	Percentage (%)	Coverage (%)
2Kb-50Kb	1192	58.00	2.09	537	51.14	0.52	1174	63.91	1.32
50Kb-100Kb	349	16.98	2.24	54	5.14	0.34	133	7.24	0.85
100Kb-150Kb	200	9.73	2.21	25	2.38	0.28	59	s3.21	0.64
150Kb-2000Kb	134	6.52	2.09	30	2.86	0.47	24	1.31	0.39
200Kb-250Kb	96	4.67	1.90	26	2.48	0.52	20	1.09	0.41
250Kb-300Kb	40	1.95	0.96	15	1.43	0.36	26	1.42	0.64
300Kb-350Kb	5	0.24	0.14	16	1.52	0.46	15	0.82	0.43
350Kb-400Kb	0	0	0	12	1.14	0.40	15	0.82	0.49
400Kb-450Kb	0	0	0	11	1.05	0.42	16	0.87	0.61
450Kb-500Kb	0	0	0	8	0.76	0.34	8	0.44	0.34
500Kb-550Kb	0	0	0	7	0.67	0.33	10	0.54	0.47
550Kb-600Kb	0	0	0	10	0.95	0.51	14	0.76	0.73
600Kb-650Kb	0	0	0	6	0.57	0.34	17	0.93	0.95
650Kb-700Kb	0	0	0	9	0.86	0.55	5	0.27	0.31
700Kb-750Kb	0	0	0	4	0.38	0.26	13	0.71	0.85
750Kb-800Kb	0	0	0	7	0.67	0.49	8	0.44	0.56
800Kb-850Kb	0	0	0	5	0.48	0.37	4	0.22	0.29
850Kb-900Kb	0	0	0	1	0.10	0.08	6	0.33	0.47
900Kb-950Kb	0	0	0	4	0.38	0.33	7	0.38	0.58
950Kb-1000Kb	0	0	0	4	0.38	0.34	7	0.38	0.61
> 1000Kb	39	1.90	88.36	259	24.67	92.43	256	13.94	89.56

[a] From the ref. 5. [b] From the ref. 6.

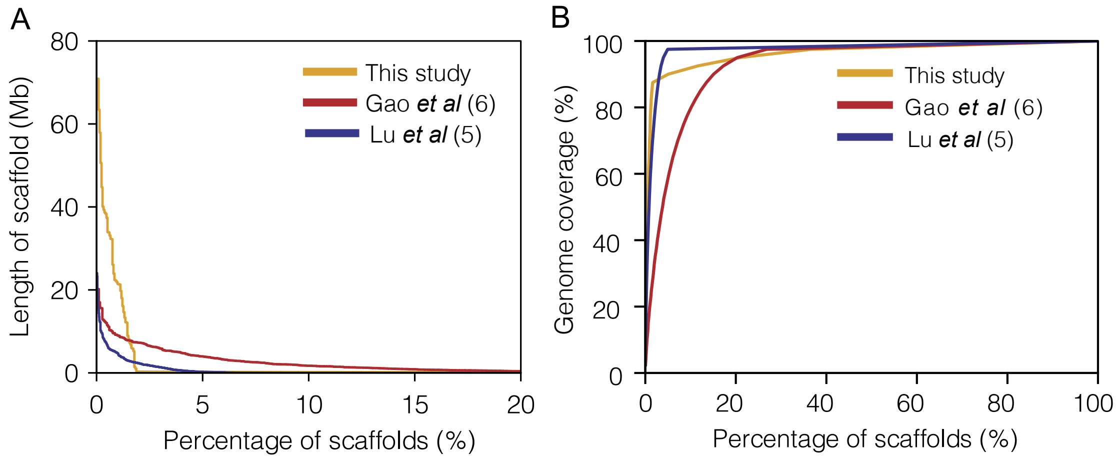

Figure 2. Comparison of the distribution and coverage of scaffolds with previous goose genome assemblies.

Comment 2-6:

Line 129-131: This statement is not supported by table 3. In fact, the other studies seem to have annotated more gene sequences than the current assembly.

Response 2-6:

Thank you for this useful comment. In this study, we annotated more repeat regions (8.67%) (Table S3) and exon sequence regions (26,883,354bp, 2.41%) (Table 3) than in previous studies (Table 3). This suggests that we have generated an improved genome assembly and annotation. We have revised lines 132–133 of the manuscript to address this point.

Comment 2-7:

Line 195-196: "... indicating that disease resistance may help .....". I don't think this statement is supported by the results and tends to be mere story telling.

Response 2-7:

Thank you for identifying this issue. In lines 198–211, we have revised the original text as follows: "Some of these PSGs, such as *GCH1* (GTP-cyclohydrolase I), are associated with parkinsonism, dystonia, and phenylketonuria disease in humans [7, 8]. They also play a role in adaptation to high-altitude environments in humans, where they relate to a lower hemoglobin level, nitric oxide concentration, and oxygen saturation in the blood. Furthermore, previous studies have shown *GCH1* divergence between

human populations living at different altitudes [9]. Selection acting on *GCH1* in goose is likely to be related to their adaption to high-altitude or migratory habitats. *SNW1* (SNW1 Domain Containing 1) is involved in the Nuclear Factor Kappa B pathway and is associated with oculopharyngeal muscular dystrophy disease [10, 11]. The depletion of this gene in breast cells leads to the induction of apoptosis, while the overexpression of this gene impedes neural crest development [12]. Selection acting on *SNW1* in goose suggests that it may confer protection from diseases and aid adaptation in changeable environments. *POU2F3* is pivotal in the discrimination of taste qualities, such as sweet, umami and bitter characteristics. Deficiency in this gene in mice alters their electrophysiology and behavioral responses to taste characters [13, 14]. Selection acting on *POU2F3* in goose is likely to be related to a requirement for seeking food in variable migratory habitats."

References

1. Burton JN, Adey A, Patwardhan RP, Qiu R, Kitzman JO, Shendure J. Chromosome-scale scaffolding of *de novo* genome assemblies based on chromatin interactions. Nat Biotechnol. 2013; 31(12): 1119-1125.
2. Ghurye J, Rhie A, Walenz BP, et al. Integrating Hi-C links with assembly graphs for chromosome-scale assembly. PLoS Comput Biol. 2019; 15(8): e1007273.
3. Liu B, Shi Y, Yuan J, et al. Estimation of genomic characteristics by analyzing k-mer frequency in de novo genome projects. 2013.
4. Wang G, Jin L, Li Y, et al. Transcriptomic analysis between Normal and high-intake feeding geese provides insight into adipose deposition and susceptibility to fatty liver in migratory birds. BMC Genomics. 2019; 20(1): 372.
5. Lu L, Chen Y, Wang Z, et al. The goose genome sequence leads to insights into the evolution of waterfowl and susceptibility to fatty liver. Genome Biol. 2015; 16(1): 89.

6. Gao G, Zhao X, Li Q, et al. Genome and metagenome analyses reveal adaptive evolution of the host and interaction with the gut microbiota in the goose. Sci Rep. 2016; 6: 32961.

7. Yoshino H, Nishioka K, Li Y, et al. GCH1 mutations in dopa-responsive dystonia and Parkinson's disease. J Neurol. 2018; 265(8): 1860-1870.

8. Gu Y, Lu K, Yang G, et al. Mutation spectrum of six genes in Chinese phenylketonuria patients obtained through next-generation sequencing. PLoS One. 2014; 9(4): e94100.

9. Guo YB, He YX, Cui CY, et al. GCH1 plays a role in the high-altitude adaptation of Tibetans. Zool Res. 2017; 38(3): 155-162.

10. Verma S, De Jesus P, Chanda SK, Verma IM. SNW1, a Novel Transcriptional Regulator of the NF-κB Pathway. Mol Cell Biol. 2019; 39(3): e00415-18.

11. Tolde O, Folk P. Stress-induced expression of p53 target genes is insensitive to SNW1/SKIP downregulation. Cell Mol Biol Lett. 2011; 16(3): 373-384.

12. Wu MY, Ramel MC, Howell M, Hill CS. SNW1 is a critical regulator of spatial BMP activity, neural plate border formation, and neural crest specification in vertebrate embryos. PLoS Biol. 2011; 9(2): e1000593.

13. Huang YH, Klingbeil O, He XY, et al. POU2F3 is a master regulator of a tuft cell-like variant of small cell lung cancer. Genes Dev. 2018; 32(13-14): 915-928.

14. Matsumoto I, Ohmoto M, Narukawa M, Yoshihara Y, Abe K. Skn-1a (Pou2f3) specifies taste receptor cell lineage. Nat Neurosci. 2011; 14(6): 685-687.
