# Supplementary material for: Pacific Biosciences assembly with Hi-C mapping generates an improved, chromosome-level goose genome
Source: Gigascience. 2020 Oct 24;9(10):giaa114. doi: 10.1093/gigascience/giaa114 (PMC7585555; doi:10.1093/gigascience/giaa114)
Supplement: giaa114_GIGA-D-20-00133_Revision_2 [file giaa114_giga-d-20-00133_revision_2.pdf]

GigaScience

PacBio assembly with Hi-C mapping generates an improved, chromosome-level goose genome

--Manuscript Draft--

**Manuscript Number:**	GIGA-D-20-00133R2
**Full Title:**	PacBio assembly with Hi-C mapping generates an improved, chromosome-level goose genome
**Article Type:**	Data Note
**Funding Information:**	National Key R & D Program of China (2018YFD0500403) — Prof. Mingzhou Li<br>National Natural Science Foundation of China (U19A2036) — Prof. Mingzhou Li<br>National Natural Science Foundation of China (31772576) — Prof. Mingzhou Li<br>National Natural Science Foundation of China (31802044) — Dr. Yan Li<br>China Postdoctoral Science Foundation (2018M643514) — Dr. Yan Li
**Abstract:**	Background:<br>The domestic goose is an economically important and scientifically valuable waterfowl; however, a lack of high-quality genomic data has hindered research concerning its genome, genetics, and breeding. As domestic geese breeds derive from both the swan goose (Anser cygnoides) and the graylag goose (Anser anser), we selected a female Tianfu goose for genome sequencing. We generated a chromosome-level goose genome assembly by adopting a hybrid de novo assembly approach that combined PacBio single-molecule real-time sequencing, high-throughput chromatin conformation capture mapping, and Illumina short-read sequencing.<br>Findings:<br>We generated a 1.11 Gb goose genome with contig and scaffold N50 values of 1.85 Mb and 33.12 Mb, respectively. The assembly contains 39 pseudo-chromosomes (2n = 78) accounting for ca. 88.36% of the goose genome. Compared with previous goose assemblies, our assembly has more continuity, completeness, and accuracy; the annotation of core eukaryotic genes and universal single-copy orthologs has also been improved. We have identified 17,568 protein-coding genes (PCGs) and a repeat content of 8.67% (96.57 Mb) in this genome assembly. W e also explored the spatial organization of chromatin and gene expression in the goose liver tissues, in terms of inter-pseudo-chromosomal interaction patterns, compartments, topologically associating domains, and promoter-enhancer interactions.<br>Conclusions:<br>We present the first chromosome-level assembly of the goose genome . Thi s will be a valuable resource for future genetic and genomic studies on geese .
**Corresponding Author:**	Mingzhou Li, Ph.D.<br>Sichuan Agricultural University<br>Chengdu, Sichuan CHINA
**Corresponding Author Secondary Information:**	
**Corresponding Author's Institution:**	Sichuan Agricultural University
**Corresponding Author's Secondary Institution:**	
**First Author:**	Yan Li
**First Author Secondary Information:**	
**Order of Authors:**	Yan Li

Powered by Editorial Manager® and ProduXion Manager® from Aries Systems Corporation

	Guangliang Gao
	Yu Lin
	Silu Hu
	Yi Luo
	Guosong Wang
	Long Jin
	Qigui Wang
	Jiwen Wang
	Qianzi Tang
	Mingzhou Li, Ph.D.
**Order of Authors Secondary Information:**	
**Response to Reviewers:**	GigaScience<br>em@editorialmanager.com<br><br>Dear Hans Zauner<br>Please find enclosed our revised manuscript, "PacBio assembly with Hi-C mapping generates an improved, chromosome-level goose genome (GIGA-D-20-00133)", which we would like to resubmit to GigaScience.<br>We sincerely appreciate the very thoughtful and constructive comments from the editor(s). We have gone in detail through all the comments and believe that we have adequately addressed all their questions and concerns. We have made all the changes in the revised version of the manuscript, and our point-by-point responses to the editor's comments are given below. We trust that the revised manuscript now meets the standards required for publication in GigaScience.<br>We look forward to hearing a positive response from you.<br>Best regards<br>Mingzhou Li<br>Ph.D/Professor/<br>Address: Institute of Animal Genetics and Breeding, College of Animal Science and Technology, Sichuan Agricultural University, Chengdu 611130, China.<br>E-mail: mingzhou.li@sicau.edu.cn<br><br>Detailed responses to Editor(s)<br>Below, our responses are in black, all revisions in the manuscript are marked in red using the word's track change.<br>____<br>Comment 1: Please include a citation to your new GigaDB dataset (including the DOI link) to your reference list, and cite this in the data availability section and elsewhere in the manuscript, where appropriate. The citation is: [xx] Li Y, Gao G, Lin Y, Hu S, Luo Y, Wang G et al. Supporting data for "PacBio assembly with Hi-C mapping generates an improved, chromosome-level goose genome" GigaScience Database. 2020. http://dx.doi.org/10.5524/100789. In the data availability section, please write something along the lines, "Supporting data, including [data type 1], [data type 2] [etc] is available via the GigaScience repository, GigaDB [xx]".<br>Response 1: In the data availability section (line 248 to 250), we added “The chromosome-level goose genome assembly, annotation files, and other supporting data are available via the GigaScience GigaDB database”, and we cited the new GigaDB dataset in line 418 to 420.<br><br>Comment 2: Do you have a picture of a representative of the goose hybrid used in your study (that can be published under a CC-BY open licence)? If you have a picture, please include this as Fig. 1, usually our "genome data note " authors show a representative of the organism/breed for illustration.

	Response 2: As the suggestion from editor (s), we supplied a new picture to represent the Tianfu goose as the Figure1, and reordered the sequence of the corresponding supplementary figures.<br><br>Comment 3: Please also add the NCBI taxon ID for the species in the methods section. https://www.ncbi.nlm.nih.gov/taxonomy (If this particular hybrid does not have its own ID, please mention the NCBI taxon IDs of A. anser and A. cygnoides).<br>Response 3: Tianfu goose is a Chinese local breed with many outstanding characteristics, such as excellent egg-laying performance, a fast growth rate, and strong adaptability. The goose belonging to Anser cygnoides domesticus (NCBI: txid381198). In line 74, we added the NCBI taxon ID (NCBI: txid381198).<br><br>Comment 4: For bioinformatics software tools you used, please add RRIDs (Reserach Resource Identifiers) in the methods section for unique identification. You can find the RRIDs here: https://scicrunch.org/resources. For example, when you first mention BUSCO in the methods section, add the following RRID in this format: (BUSCO, RRID:SCR_015008).<br>Response 4: In the manuscript and supplemental material files, we supplied RRIDs for most of software and marked the changes in red. However, we did not found the RRIDs for the TACO software (line 130 of the text) and the PSYCHIC software (line 86 of the supplemental material) using the website (https://www.ncbi.nlm.nih.gov/taxonomy) or google search engine.<br><br>Moreover, we also made changes elsewhere in the text.<br>1.In the List of abbreviations section, we revised the “Anser anser: A. anser” to “A. anser: Anser anser” in line 252. In line 253, we revised “Anser cygnoides: A. cygnoides” to “A. cygnoides: Anser cygnoides”.<br>2.In line 285, we deleted the funding National Natural Science Foundation of China (31872335).
**Additional Information:**	
**Question**	**Response**
Are you submitting this manuscript to a special series or article collection?	No
**Experimental design and statistics**<br><br>Full details of the experimental design and statistical methods used should be given in the Methods section, as detailed in our Minimum Standards Reporting Checklist. Information essential to interpreting the data presented should be made available in the figure legends.<br><br>Have you included all the information requested in your manuscript?	Yes
**Resources**<br><br>A description of all resources used, including antibodies, cell lines, animals and software tools, with enough information to allow them to be uniquely	Yes

identified, should be included in the Methods section. Authors are strongly encouraged to cite Research Resource Identifiers (RRIDs) for antibodies, model organisms and tools, where possible.<br><br>Have you included the information requested as detailed in our Minimum Standards Reporting Checklist?	
**Availability of data and materials**<br><br>All datasets and code on which the conclusions of the paper rely must be either included in your submission or deposited in publicly available repositories (where available and ethically appropriate), referencing such data using a unique identifier in the references and in the “Availability of Data and Materials” section of your manuscript.<br><br>Have you have met the above requirement as detailed in our Minimum Standards Reporting Checklist?	Yes

Manuscript

Click here to access/download;Manuscript;GigaScience_Manuscript.docx

PacBio assembly with Hi-C mapping generates an improved, chromosome-level goose genome

Yan Li[1,†], Guangliang Gao[1,2,†,*], Yu Lin[1,†], Silu Hu[1], Yi Luo[1], Guosong Wang[1,3], Long Jin[1], Qigui Wang[2], Jiwen Wang[1], Qianzi Tang[1], Mingzhou Li[1,*]

[1]Institute of Animal Genetics and Breeding, College of Animal Science and Technology, Sichuan Agricultural University, Chengdu 611130, China;

[2]Institute of Poultry Science, Chongqing Academy of Animal Sciences, Chongqing 402460, China;

[3]Department of Animal Science, Texas A&M University, College Station 77843, United States of America

[†] These authors contributed equally to this paper.

[*] Corresponding author(s): Guangliang Gao: guanglianggaocq@hotmail.com; Mingzhou Li: mingzhou.li@sicau.edu.cn.

Abstract

Background:

The domestic goose is an economically important and scientifically valuable waterfowl; however, a lack of high-quality genomic data has hindered research concerning its genome, genetics, and breeding. As domestic geese breeds derive from both the swan goose (*Anser cygnoides*) and the graylag goose (*Anser anser*), we selected a female Tianfu goose for genome sequencing. We generated a chromosome-level goose genome assembly by adopting a hybrid *de novo* assembly approach that combined PacBio single-molecule real-time sequencing, high-throughput chromatin conformation capture mapping, and Illumina short-read sequencing.

Findings:

We generated a 1.11 Gb goose genome with contig and scaffold N50 values of 1.85 Mb and 33.12

Mb, respectively. The assembly contains 39 pseudo-chromosomes (2n = 78) accounting for ca. 88.36% of the goose genome. Compared with previous goose assemblies, our assembly has more continuity, completeness, and accuracy; the annotation of core eukaryotic genes and universal single-copy orthologs has also been improved. We have identified 17,568 protein-coding genes (PCGs) and a repeat content of 8.67% (96.57 Mb) in this genome assembly. We also explored the spatial organization of chromatin and gene expression in the goose liver tissues, in terms of inter-pseudo-chromosomal interaction patterns, compartments, topologically associating domains, and promoter-enhancer interactions.

Conclusions:

We present the first chromosome-level assembly of the goose genome. This will be a valuable resource for future genetic and genomic studies on geese.

Key Words: goose genome, chromosome-length assembly, hybrid *de novo* assembly approaches, annotation, Pacbio, Hi-C

Data description

Context

The goose is a member of the family Anatidae and is an economically important waterfowl with distinctive characters. Domesticated geese derive from the swan goose (*Anser cygnoides*) and the graylag goose (*Anser anser*)[1], and approximately 6,000 years of artificial selection have led to significant alterations in their body size, reproductive performance, egg production, feather color, and other features[2]. Currently, more than 181 domesticated breeds are reared globally to supply meat, eggs, and valuable byproducts (feathers, fatty liver) for human consumption[2,3,4]. The domestic goose is also well suited to sustainable production practices because fiber can form part of its diet, which then lessens competition for human food[5]. Its excellent disease resistance and behavioral patterns also allow for large-scale

farming and easy management[6]. Interestingly, despite the liver weight of goose increasing 5–10 times after two to three weeks of overfeeding, the amount of fat in hepatic cells (and other biomedical parameters) returns to normal levels when overfeeding ceases. This suggests that the goose liver could provide a novel animal model for the study of human non-alcoholic fatty liver disease[6].

The goose was one of the earliest animals to be domesticated[2,7], and wide-ranging genomic and breeding research has been conducted to study its domestication process and the unique morphological and physiological features of these animals. For example, recently published goose genome sequences have been assembled into scaffolds using short reads from the Illumina platform[8,9]; however, the genetic basis of the fatty liver of goose and their selective breeding remains largely unknown. To address such issues, a high-quality genome sequence is required. Currently, there are many advantages to using hybrid *de novo* assembly approaches to improve the quality of genome assemblies. This is because short, accurate reads from the Illumina platform can be combined with the longer, less accurate reads generated by the single-molecule real-time (SMRT) sequencing platform[10]. With Hi-C, linking information can then be ordered and oriented into scaffolds, after which assembly errors can be identified and corrected[11]. This approach has been applied to improve the genome assemblies of many species, including humans[12], goats[13], rockfish[14], *Aedes aegypti*[11], and barley[15].

Here, we have generated a chromosome-level goose assembly with chromosome-length scaffolds by adopting a hybrid *de novo* assembly approach using a combination of short reads from the Illumina platform, long reads from the PacBio platform, and Hi-C-based chromatin interaction maps. Our chromosome-level goose genome comprises longer scaffolds than currently available goose genome assemblies, and these scaffolds are of a higher-quality and are more continuous and accurate. Our new genome assembly thus provides a valuable resource for exploring the molecular basis of the

morphological and physiological features of the goose, and will facilitate further genomic, genetic, and breeding studies of this domesticated waterfowl.

Methods

a) Sample collection and sequencing

We extracted genomic DNA from the liver tissue of a healthy adult female (136 days old) from the Tianfu goose maternal line (NCBI: txid381198), which was provided by the Experimental Farm of Waterfowl Breeding of Sichuan Agricultural University (Chengdu, Sichuan, China; **Figure 1**). We then carried out single-molecule real-time DNA sequencing of ca. 20-kb inserts using the PacBio Sequel platform. This yielded approximately 84.31 Gb of high-quality sequencing data that were used to initially assemble the genome (**Table 1**). Next, 149.70 Gb of high-quality sequencing data were generated from a 350-bp insert size Hi-C library, as previously reported[13]. Finally, 350-bp paired-end libraries constructed from the same genomic DNA were sequenced on the Illumina HiSeq platform, producing a further 181.52 Gb of sequence data. In total, we obtained approximately 415.53 Gb sequencing data (ca. 324.63× coverage) for our chromosome-level goose genome assembly (**Table 1**).

b) *De novo* assembly of the goose genome

The size of the goose genome was estimated by k-mer distribution analysis to be 1.28 Gb. To assemble the genome, we first performed an initial assembly with the PacBio long-reads alone, using Falcon (Falcon, RRID:SCR_016089)[16] software. We used the pbsmrtpipe pipeline of the smrtlink (smrtlink, RRID:SCR_002942) software to assembly the genome sequence, which resulted in a draft assembly with a contig N50 of 1.72 Mb (**Table S1**). Next, we used the single-molecule sequence reads to scaffold these contigs and fill gaps, using SSPACE-Long (SSPACE-Long, RRID:SCR_005056)[17] and PBJelly (PBJelly, RRID:SCR_012091)[18], respectively. Pilon (Pilon, RRID:SCR_014731)[19] software was

then used to map the short reads to the assembly (**Table S1**). Finally, 39 pseudo-chromosomes were assembled with the Hi-C reads were aligned using Lachesis (Lachesis, RRID:SCR_017644)[20] software (**Table S2, Figure S1**); this is consistent with the number of goose chromosomes (2n = 78) reported in previous studies[21]. With these methods, we generated a chromosome-level goose assembly with a contig N50 of 1.85 Mb and scaffold N50 of 33.12 Mb (**Table 2**). The average GC content is 42.15% and the total genome size is 1.11 Gb, which is consistent with previous studies[8,9] and suggests that our goose assembly is reliable.

c) Repeat sequence and gene annotation

De novo methods and homology-based approaches were used to annotate the repeat content of the goose genome. First, we used *ab initio*-prediction software, including LTR-finder (LTR-finder, RRID:SCR_005659)[22], RepeatMolder [2](RepeatMolder, RRID:SCR_015027)[3], and RepeatScout (RepeatScout, RRID:SCR_014653)[24], to perform *de novo* annotation of the genome. For homology-based predictions, we identified repeat regions across species in published RepBase sequences[25] using RepeatMasker (RepeatMasker, RRID:SCR_012954)[26] and RepeatProteinMask (RepeatProteinMask, RRID:SCR_012954)[27] software. Combined with these results, the repeat region of the goose genome was further predicted with RepeatMasker software. From these analyses, we identified 92.11 Mb of repetitive DNA (**Table S3**) accounting for 8.67% of our assembly, which is much higher than has been reported in previous studies[8,9]. Long interspersed nuclear elements (LINEs) were the most abundant repeat element identified, representing 6.83% of the genome. The proportion of LINE repetitive sequences identified in this study was also higher than has been reported in two previous goose genome assemblies (**Table S3**). We performed PCGs annotation by combining *ab initio*-based, homology-based, and RNA-sequencing-based prediction methods. First, GenScan (GenScan, RRID:SCR_012902)[28],

Geneid (Geneid, RRID:SCR_002473)[29], and Augustus (Augustus, RRID:SCR_008417)[30] were used for *ab initio*-based predictions. Next, we selected six chromosome-level genomes, namely *Homo sapiens* (GCF_000001405.39), *Mus musculus* (GCF_000001635.26), *Gallus gallus* (GCF_000002315.6), *Anas platyrhynchos* (GCF_003850225.1), *Meleagris gallopavo* (GCF_000146605.3), and *Taeniopygia guttata* (GCF_003957565.1), to use for homology-based annotation of our goose chromosome-level assembly genome using TBLASTN (TBLASTN, RRID:SCR_011822)[31] and GeneWise (GeneWise, RRID:SCR_015054)[32] software. We found 8,255 common orthologous groups across these seven species (**Figure S2**). To optimize genome annotation, total RNA was extracted from 11 samples (abdominal fat, brain, duodenum, heart, liver, lung, muscular stomach, ovary, pancreas, pectoral muscle, and spleen) taken from the same individual whose DNA was used for the chromosome-level genome assembly. We pooled equal amounts of the total RNA from each of the 11 tissues and then performed RNA-seq on this pooled sample using the Illumina platform. After filtering, these data were used to annotate protein-coding regions of the genome assembly using Trinity (Trinity, RRID:SCR_013048)[33] and TopHat (TopHat, RRID:SCR_013035)[34]. Finally, the predictions from each method described above were integrated using EVM (EVM, RRID:SCR_014659)[35]; overall, 17,568 PCGs were predicted (**Table 3, Figure S3**). To identify long noncoding RNAs (lncRNAs), the goose genome reads were aligned by STAR (STAR, RRID:SCR_015899)[36] and subjected to Cufflinks (Cufflinks, RRID:SCR_014597)[37] and TACO[38] for assembly and filtering. CPC2(CPC2, RRID:SCR_002764)[39] was then applied to perform coding potential analysis, and PfamScan (PfamScan, RRID:SCR_004726)[40] was used to check for domain hits against Pfam31-A[41]. After removing all likely domains, 3,287 lncRNAs only by ab initio assembly method and 542 transcripts of uncertain coding potential (TUCP) were identified, the long reads will be helpful to improve the identification and annotation of the lncRNA and TUCP in goose

genome.

Data validation and quality control

a) Assessment of genome assembly completeness

Our assembly has more scaffolds and fewer contigs, and significantly improved contig and scaffold N50 values, than the goose genome assemblies presented in two previous studies (**Figure 2**). Moreover, we have annotated more repeat (Table S3) and exons sequence regions (Table 3) than these previous studies (**Table 3**), which suggests that we have generated an improved genome assembly and annotation. The 39 pseudo-chromosomes described in our study account for 88.36% of the assembled genome and are longer than those previously reported[8,9], again indicating that our chromosome-level goose genome represents a significant improvement on previous work. The GC content of our genome assembly is 42% and the size of the genome is 1.11 Gb (**Table 2**). This is comparable to the sizes reported for the two previously constructed goose genomes[8,9] and is characteristic of avian genomes[42]. We also mapped short-insert paired-end reads (350 bp) to our chromosome-level goose genome and obtained mapping and coverage rates of 97.25% and 99.71%, respectively. Finally, we downloaded 19 wild goose resequencing[43] datasets from public databases and mapped them to our assembly, and to the two earlier draft goose genomes. We found that the mapping rate of our chromosome-level goose assembly was higher than that of the previously assembled genomes (**Table S4**), indicating that it is more contiguous. Taken together, these results demonstrate the improvements made by our study in the assembly and annotation of the goose genome, in comparison to previous studies[8,9].

To evaluate the completeness of our chromosome-level genome assembly, we determined the number of conserved eukaryotic and universal genes present in our assembly by applying the core eukaryotic genes mapping approach software (CEGMA, RRID:SCR_015055) and using a set of

benchmarking universal single-copy orthologs (BUSCO, RRID:SCR_015008). We found that 211 of the 248 (85.08%) core eukaryotic genes and 2,586 (97%) of the universal single-copy orthologs were assembled in our genome. Compared with previous studies, this suggests that our genome assembly is more complete than previous drafts of the goose genome[8,9].

To explore the hypothesis that the leptin gene was lost from goose[8], we downloaded leptin sequences from avian and mammal genomes to use as reference sequences in BLASTP (BLASTP, RRID:SCR_001010) searches of our newly assembled goose genome. We found no sequences similar to leptin in our chromosome-level goose assembly. Furthermore, although the human genome region that contains the leptin gene (chromosome 7, 126.0 to 129.4 Mb) aligned with the goose genome, we did not find a sequence similar to the leptin gene in this region. These results confirm the previous finding that the leptin gene is not present in the goose genome[8].

b) Phylogenetic tree and lineage-specific gene families

Using OrthoMCL (OrthoMCL, RRID:SCR_007839)[44], 16,157 orthologous gene families across 17 species (ostrich, duck, goose, chicken, turkey, saker, red-legged seriema, African crowned crane, pelican, little egret, crested ibis, cormorant, great crested grebe, pigeon, woodpecker, zebra finch, and lizard) were identified. Based on 2,389 shared single-copy ortholog gene clusters, we constructed a maximum likelihood phylogenetic tree using the RAxML software (RAxML, RRID:SCR_006086)[45]. This revealed that goose and duck diverged about 31.60 million years ago (Mya), which is comparable to the divergence time of chicken and turkey (32.33 Mya; **Figure S4**) and consistent with the previous studies [8, 9]. We also noted that lineage-specific genes in the goose genome were significantly enriched for olfactory receptor activity (GO:0004984, p = 3.85×10^{-24}), G protein-coupled receptor activity (GO:0004930, $p = 6.67\times10^{-13}$), and integral component of membrane (GO:0016021, $p = 0.01$; **Table S5**).

As a migratory bird, the goose is adapted for long-distance migration, which exposes them to a diversity of food as they seek out ideal habitats. We propose that such influences might strengthen the interactions between odorants and the receptors of the olfactory mucosa, and could underlie receptor family evolution in the goose genome.

c) Expansion and contraction of gene families

The expansions and contractions of gene clusters in the goose genome were identified in comparison to nine other avian genomes using the CAFE program (CAFÉ, RRID:SCR_018924)[46]. We found 839 expanded gene families (**Table S6**) and 2,193 contracted gene families (**Table S7**). Interestingly, the expanded gene families were mainly enriched for olfactory receptor activity (GO:0004984, $p = 8.58\times10^{-51}$), G protein-coupled receptor activity (GO:0004930, $p = 5.81\times10^{-25}$), and integral component of membrane (GO:0016021, $p = 3.20\times10^{-6}$), which is consistent with the results from our analysis of lineage-specific genes (**Table S5**). This further confirms that the migratory adaptations of the goose are reflected by unique characteristics in the goose genome that contrast with those of nonmigratory birds. Other expanded gene families were enriched for ATPase-coupled transmembrane transporter activity (GO:0042626, $p = 1.96\times10^{-06}$), NAD(P)+-protein-arginine ADP-ribosyl transferase activity (GO:0003956, $p = 3.20\times10^{-04}$), ATPase activity (GO:0016887, $p = 8.28\times10^{-05}$), and aspartic-type endopeptidase activity (GO:0004190, $p = 9.63\times10^{-06}$; **Table S6**), while gene families contracted in the goose were significantly enriched for transmembrane transport (GO:0055085, $p = 8.30\times10^{-04}$), ion channel activity (GO:0005216, $p = 1.87\times10^{-9}$), ion transmembrane transport (GO:0034220, $p = 5.30\times10^{-6}$), and ATPase-coupled intramembrane lipid transporter activity (GO:0140326, $p = 8.60\times10^{-10}$; **Table S7**). As these pathways are related to ATP utilization, ATP production, and energy regulation, these data support a previous finding that goose energy metabolism is different from that in other avian

species[47]. This feature of the goose is possibility related to its migratory habits and artificial selection—the goose is unique among migratory birds because of its large body size, which requires much energy for long-distance, high altitude flying[48].

d) Genes under positive selection

We identified 52 positively selected genes (PSGs) in the goose genome based on orthologous genes from the 17 species above, using a branch-site model and F3x4 codon frequencies in Codeml (Codeml, RRID:SCR_004542) (**Table S8**). Some of these PSGs, such as *GCH1* (GTP-cyclohydrolase I), are associated with parkinsonism, dystonia, and phenylketonuria disease in humans[49, 50]. They also play a role in adaptation to high-altitude environments in humans, where they relate to a lower hemoglobin level, nitric oxide concentration, and oxygen saturation in the blood. Furthermore, previous studies have shown *GCH1* divergence between human populations living at different altitudes[51]. Selection acting on *GCH1* in goose is likely to be related to their adaption to high-altitude or migratory habitats. *SNW1* (SNW1 Domain Containing 1) is involved in the Nuclear Factor Kappa B pathway and is associated with oculopharyngeal muscular dystrophy disease[52, 53]. The depletion of this gene in breast cells leads to the induction of apoptosis, while the overexpression of this gene impedes neural crest development[54]. Selection acting on *SNW1* in goose suggests that it may confer protection from diseases and aid adaptation in changeable environments. *POU2F3* is pivotal in the discrimination of taste qualities, such as sweet, umami and bitter characteristics. Deficiency in this gene in mice alters their electrophysiology and behavioral responses to taste characters[55,56]. Selection acting on *POU2F3* in goose is likely to be related to a requirement for seeking food in variable migratory habitats.

e) Initial characterization of the three-dimensional organization of goose liver tissues

We analyzed the inter-pseudo-chromosomal interaction pattern[57], compartments[58, 59], topologically

associating domains (TADs)[60], and promoter-enhancer interactions (PEI)[61] of the goose liver tissue. The matrix resolution of our Hi-C experiment reached ~2 Kb (defined as the smallest locus size such that 80% of loci have at least 1,000 contacts) (**Figure S5**), which was adequate for subsequent analyses of the chromatin architecture. Our results showed that the whole inter-pseudo-chromosomal interaction pattern was distinguished by two clusters, that is, short pseudo-chromosomes and longer pseudo-chromosomes, which suggests that goose pseudo-chromosomes tend to interact with one another on the basis of size (**Figure 3**). As for the identification of A and B compartments, which represent relatively active and inactive chromatin states, respectively, the number of protein-coding genes (PCGs) in each 100 Kb bin with at least 50 % percentage overlapped with a gene was counted. The number of PCGs was significantly correlated with PC1 values (R = 0.39, $p = 2.2\times10^{-16}$; **Figure S6**), and the transcripts per kilobase millions (TPMs) of PCGs located in A compartments were consistently higher than PCGs in B compartments in three liver tissues ($p = 2.2\times10^{-16}$; **Figure S7, Table S9**). We identified 734 TADs across the goose assembly, accounting for 80% of the genome (**Figure S8, Table S10**). The mean and median sizes of the TADs were 1.21 Mb and 1.00 Mb, respectively. We also observed that the TSSs of PCGs were enriched in TAD-boundary regions (**Figure S9**). After filtering for interaction distances lower than 20 Kb, we identified 13,017 PEIs (**Table S11**) and found that gene expression levels positively correlated with the number of its associated enhancers in all three liver tissues (**Figure S10**). This is suggestive of additive effects of enhancers on target-gene transcription levels.

Availability of supporting data

The chromosome-level goose genome assembly sequence is available at National Center for Biotechnology Information (NCBI) GenBank through the accession number WTSS00000000; The high-quality Hi-C data are available through the NCBI Sequence Read Archive (SRA) database under

accession number SRR10483522. The PacBio long-read sequencing data have been deposited in the NCBI SRA (SRR10483521). The high-quality Illumina short-read sequencing data are available through NCBI SRA accession number: SRR10483516, SRR10483517, SRR10483518 and SRR10483520. The transcriptome data are available through the NCBI SRR10483519. The chromosome-level goose genome assembly, annotation files, and other supporting data are available via the *GigaScience* GigaDB database [62].

List of abbreviations

(1) A. anser: Anser anser;

(2) A. cygnoides: Anser cygnoides;

(3) BUSCO: Benchmarking Universal Single-Copy Orthologs;

(4) CHMP1B: charged multivesicular body protein 1B;

(5) CEGMA: Core Eukaryotic Genes Mapping Approach software;

(6) TUCP: transcripts of uncertain coding potential;

(7) GCH1: GTP cyclohydrolase 1;

(8) Hi-C, Chromosome conformation capture;

(9) IVNS1ABP: influenza virus NS1A binding protein;

(10) LINEs: Long interspersed nuclear elements;

(11) LncRNAs: long noncoding RNAs;

(12) OGFOD2: 2-oxoglutarate and iron dependent oxygenase domain containing 2

(13) MDH257: malate dehydrogenase 2

(14) PCGs: protein coding genes

(15) PEI: promoter-enhancer interactions;

(16) PSGs: positively selected genes;

(17) SMRT: single-molecule real-time;

(18) TADs: topological associated domains;

(19) TPMs: transcripts per kilobase millions.

Ethics approval

All animal experiments were approved and reviewed by Animal Care and Use Committee Institutional of Sichuan Agricultural University (Approval No. DKY-B20121406) and the Ministry of Science and Technology of the People's Republic of China (Approval No. 2006–398).

Competing interests

The authors declare no competing interest.

Acknowledgments

This work was supported by grants from the National Key R & D Program of China (2018YFD0500403), the National Natural Science Foundation of China (U19A2036, ~~31872335,~~ 31772576 and 31802044) and the China Postdoctoral Science Foundation (2018M643514).

Author contributions

Mingzhou Li, Guangliang Gao designed and supervised the project. Yan Li, Yu Lin, Qianzi Tang, Silu Hu performed bioinformatics analyses. Jiwen Wang, Yan Li and Yi Luo contributed to collect the samples. Mingzhou Li, Qigui Wang, Guangliang Gao, Yi Luo and Long Jin were involved in the data analyses and wrote the manuscript.

References

1. Shi XW, Wang JW, Zeng FT, et al. Mitochondrial DNA cleavage patterns distinguish independent origin of Chinese domestic geese and western domestic geese. Biochem Genet. 2006; 44(5-6) : 237-245.
2. Kozák J. Variations of geese under domestication. World's Poult Sci J. 2019; 75(2): 247-260.

3. Goluch-Koniuszy Z, Haraf G. Geese for slaughter and wild geese as a source of selected mineral elements in a diet. J Elementol. 2018; 23: 1343-1360.

4. Janan J, Tóth P, Hutas I, et al. Effects of dietary micronutrient supplementation on the reproductive traits of laying geese. Acta Fytotech Zootech. 2015; 18(1) : 6-9.

5. Zhang Y, Sha Z, Guan F, et al. Impacts of geese on weed communities in corn production systems and associated economic benefits. Biol Control. 2016. 99: 47-52.

6. Wang G, Jin L, Li Y, et al. Transcriptomic analysis between Normal and high-intake feeding geese provides insight into adipose deposition and susceptibility to fatty liver in migratory birds. BMC genomics. 2019; 20(1): 372.

7. Honka J, Heino M, Kvist L, et al. Over a thousand years of evolutionary history of domestic geese from Russian archaeological sites, analysed using ancient DNA. Genes. 2018; 9(7): 367.

8. Lu L, Chen, Y, Wang Z, et al. The goose genome sequence leads to insights into the evolution of waterfowl and susceptibility to fatty liver. Genome Biol. 2015; 16(1): 89.

9. Gao G, Zhao X, Li Q, et al. Genome and metagenome analyses reveal adaptive evolution of the host and interaction with the gut microbiota in the goose. Sci Rep. 2016; 6: 32961.

10. Schadt E, Turner S, Kasarskis A. A window into third-generation sequencing. Hum Mol Genet. 2010; 19(R2): R227-R240.

11. Dudchenko O, Batra SS, Omer AD, et al. *De novo* assembly of the *Aedes aegypti* genome using Hi-C yields chromosome-length scaffolds. Science. 2017; 356(6333): 92-95.

12. Pendleton M, Sebra R, Pang AWC, et al. Assembly and diploid architecture of an individual human genome via single-molecule technologies. Nat Methods. 2015; 12(8): 780–786.

13. Bickhart DM, Rosen BD, Koren S, et al. Single-molecule sequencing and chromatin conformation

capture enable *de novo* reference assembly of the domestic goat genome. Nat Genet. 2017; 49(4): 643.

14. Liu Q, Wang X, Xiao Y, et al. Sequencing of the black rockfish chromosomal genome provides insight into sperm storage in the female ovary. DNA Research, 2019. 26(6):453–464,
15. Mascher M, Gundlach H, Himmelbach A, et al. A chromosome conformation capture ordered sequence of the barley genome. Nature. 2017; 544(7651): 427
16. Chin CS, Peluso P, Sedlazeck FJ et al. Phased diploid genome assembly with single molecule real-time sequencing. Nat Methods. 2016;13:1050.
17. Boetzer M, Pirovano W. SSPACE-LongRead: scaffolding bacterial draft genomes using long read sequence information. BMC Bioinf. 2014; 15(1): 211.
18. English AC, Richards S, Han Y, et al. Mind the gap: upgrading genomes with Pacific Biosciences RS long-read sequencing technology. PLoS One. 2012; 7(11): e47768.
19. Walker BJ, Abeel T, Shea T, et al. Pilon: an integrated tool for comprehensive microbial variant detection and genome assembly improvement. Plos One. 2014; 9(11): e112963.
20. Burton JN, Adey A, Patwardhan RP, et al. Chromosome-scale scaffolding of *de novo* genome assemblies based on chromatin interactions. Nat Biotechnol. 2013; 31(12): 1119–1125.
21. Jun X, Tianxing L, Qing C, et al. Karyotypes of Zhedong White Goose and Siji Goose. China Poultry. 2007; 21(9): 27-29.
22. Benson G. Tandem repeats finder: a program to analyze DNA sequences. Nucleic Acids Res. 1999; 27(2): 573–580.
23. RepeatMolder software. http://www.repeatmasker.org/RepeatModeler/.
24. Price AL , Jones NC, Pevzner PA. De novo identification of repeat families in large genomes.

Bioinformatics2005;21(suppl 1):i351–8.

25. Price AL, Jones NC, Pevzner PA. De novo identification of repeat families in large genomes. Bioinformatics. 2005;21:i351–8. Bao W, Kojima KK, Kohany O. Repbase Update, a database of repetitive elements in eukaryotic genomes. Mobile DNA. 2015; 6(1):11.

26. Maja TG, Nansheng C. Using RepeatMasker to identify repetitive elements in genomic sequences. Curr Protoc Bioinf. 2009; 25(1): 4.10.11–14.10.14.

27. Allred DB, Cheng A, Sarikaya M, et al. . Three-dimensional architecture of inorganic nanoarrays electrodeposited through a surface-layer protein mask. Nano Lett. 2008;8(5):1434–8.

28. Burge C, Karlin S. Prediction of complete gene structures in human genomic DNA. J Mol Biol. 1997; 268(1): 78–94.

29. Blanco E, Parra G, Guigó R. Using geneid to identify genes. Curr Protoc Bioinf. 2007; 18(1): 4.3.1-4.3.28.

30. Stanke M, Steinkamp R, Waack S. AUGUSTUS: a web server for gene finding in eukaryotes. Nucleic Acids Res. 2004; 32(suppl_2): W309–W312.

31. Gertz EM, Yu YK. Agarwala, R., Schäffer, A. A. & Altschul, S. F. Composition-based statistics and translated nucleotide searches: improving the TBLASTN module of BLAST. BMC Biol. 2006; 4(1): 41.

32. Birney E, Clamp M, Durbin R. Gene Wise and Genomewise. Genome Res. 2004; 14(5): 988–995.

33. Grabherr MG, Haas BJ, Yassour M, et al. Full-length transcriptome assembly from RNA-Seq data without a reference genome. Nat Biotechnol. 2011; 29(7): 644–652.

34. Trapnell C, Pachter L, Salzberg SL. TopHat: discovering splice junctions with RNA-Seq. Bioinformatics. 2009; 25(9): 1105–1111.

35. Haas BJ, Salzberg SL, Zhu W, et al. Automated eukaryotic gene structure annotation using EVidenceModeler and the Program to Assemble Spliced Alignments. Genome Biol. 2008; 9(1): R7.
36. Dobin A, Davis CA, Schlesinger F, et al. STAR: ultrafast universal RNA-seq aligner. Bioinformatics. 2013; 29(1): 15–21.
37. Trapnell C, Williams BA, Pertea G, et al. Transcript assembly and quantification by RNA-Seq reveals unannotated transcripts and isoform switching during cell differentiation. Nat Biotechnol. 2010; 28(5): 511–515.
38. Niknafs YS, Pandian B, Iyer HK, et al. TACO produces robust multisample transcriptome assemblies from RNA-seq. Nat Methods. 2017; 14(1): 68.
39. Kang YJ, Yang DC, Kong L, et al. CPC2: a fast and accurate coding potential calculator based on sequence intrinsic features. Nucleic Acids Res. 2017; 45(W1): W12-W16.
40. Finn RD, Bateman A, Clements J, et al. Pfam: the protein families database. Nucleic Acids Res. 2014; 42(D1): D222–D230.
41. Bateman A, Coin L, Durbin R, et al. The Pfam protein families database. Nucleic Acids Res. 2004; 32 (suppl_1): D138–D141.
42. Zhang G, Li C, Li Q, et al. Comparative genomics reveals insights into avian genome evolution and adaptation. Science. 2014; 346(6215): 1311-1320.
43. Ottenburghs J, Megens HJ, Kraus RH, et al. A history of hybrids? Genomic patterns of introgression in the True Geese. BMC Evol Biol. 2017; 17(1): 201.
44. Fischer S, Brunk BP, Chen F, et al. Using OrthoMCL to assign proteins to OrthoMCL- DB groups or to cluster proteomes into new ortholog groups. Curr Protoc Bioinf. 2011; 35(1): 6-12.
45. Stamatakis A. RAxML version 8: a tool for phylogenetic analysis and post-analysis of large

phylogenies. Bioinformatics. 2014; 30(9): 1312–1313.

46. Bie T, Cristianini N, Demuth J. CAFE: a computational tool for the study of gene family evolution. Bioinformatics. 2006; 22(10): 1269-1271.

47. Józefiak DA, Rutkowski A, Martin SA. Carbohydrate fermentation in the avian ceca: a review. Anim Feed Sci Technol. 2004; 113(1-4): 1-15.

48. Watanabe, YY. Flight mode affects allometry of migration range in birds. Ecol Lett. 2016; 19(8): 907-914.

49. Yoshino H, Nishioka K, Li Y, et al. GCH1 mutations in dopa-responsive dystonia and Parkinson's disease[J]. J Neuro, 2018, 265(8): 1860-1870.

50. Gu Y, Lu K, Yang G, et al. Mutation spectrum of six genes in Chinese phenylketonuria patients obtained through next-generation sequencing[J]. PLoS One, 2014, 9(4): e94100.

51. He Y B, Duojizhuoma C Y, Cai-juan D B, et al. GCH1 plays a role in the high-altitude adaptation of Tibetans. Zool Res. 2017; 38(3): 155–162.

52. Verma S, De Jesus P, Chanda S K, et al. SNW1, a novel transcriptional regulator of the NF-κB pathway. Mol Cell Biol. 2019; 39(3): e00415-18.

53. Tolde O, Folk P. Stress-induced expression of p53 target genes is insensitive to SNW1/SKIP downregulation[J]. Cell Mol Biol Lett, 2011, 16(3): 373-384.

54. Wu M Y, Ramel M C, Howell M, et al. SNW1 is a critical regulator of spatial BMP activity, neural plate border formation, and neural crest specification in vertebrate embryos[J]. PLoS Biol, 2011, 9(2): e1000593.

55. Huang Y H, Klingbeil O, He X Y, et al. POU2F3 is a master regulator of a tuft cell-like variant of small cell lung cancer. Gene Dev. 2018; 32(13-14): 915-928.

56. Matsumoto I, Ohmoto M, Narukawa M, et al. Skn-1a (Pou2f3) specifies taste receptor cell lineage[J]. Nat Neurosci, 2011, 14(6): 685.

57. Battulin N, Fishman VS, Mazur AM, et al. Comparison of the three-dimensional organization of sperm and fibroblast genomes using the Hi-C approach. Genome Biol. 2016; 17(1): 6.

58. Lieberman-Aiden E, van Berkum NL, Williams L, et al. Comprehensive mapping of long-range interactions reveals folding principles of the human genome. Science. 2009; 326(5950): 289-293.

59. Rowley MJ, Nichols MH, Lyu X, Ando-Kuri M, et al. Evolutionarily Conserved Principles Predict 3D Chromatin Organization. Mol Cell. 2017; 67(5): 837-852.

60. Dixon JR, Selvaraj S, Yue F, et al. Topological domains in mammalian genomes identified by analysis of chromatin interactions. Nature. 2012; 485(7389): 376-380.

61. Ron G, Globerson Y, Moran D and Kaplan T. Promoter-enhancer interactions identified from Hi-C data using probabilistic models and hierarchical topological domains. Nat Commun. 2017; 8(1): 2237.

62. Li Y, Gao G, Lin Y, Hu S, Luo Y, Wang G et al. Supporting data for "PacBio assembly with Hi-C mapping generates an improved, chromosome-level goose genome" GigaScience Database. 2020. http://dx.doi.org/10.5524/100789

Click here to access/download;Table;Table 1.docx

Table1 Summary of sequencing data for goose genome assembly.

Pair-end libraries	Insert size (bp)	Total data (Gb)	Read length (bp)	Sequence coverage (×)
**Illumina reads**	350	181.52	150	141.81
**Pacbio reads**	20,000	84.31		65.86
**Hi-C**	350	149.70	150	116.95
**Total**		415.53		324.63

Table2 Comparison of quality metrics of this study and the previous goose genome assemblies. Click here to access/download;Table;Table 2.docx

Table2 Comparison of quality metrics of this study and the previous goose genome assemblies.

Genomic features	This study	Lu *et al.*[a]	Gao *et al.*[b]
Estimation of genome size (bp)	1,277,099,016	1,208,661,181	1,198,802,839
Total length of assembled contigs (bp)	1,113,842,245	1,086,838,604	1,100,859,441
Total size of assembled scaffolds (bp)	1,113,913,845	1,122,178,121	1,130,663,797
Number of contigs (>2kb)	2,771	60,979	53,336
Number of scaffolds （>2kb）	2,055	1,050	1,837
Contigs N50 (bp)	1,849,874	27,602	35,032
Scaffolds N50 (bp)	33,116,532	5,202,740	5,103,766
Longest contig (bp)	10,766,871	201,281	399,111
Longest scaffold (bp)	70,896,740	24,051,356	20,207,557
GC content (%)	42.15	38.00	41.68
Number of gene model	17,568	16,150	16,288
Repetitive regions percentage of genome (%)	8.67	6.33	6.90

[a] From the ref. 8. [b] From the ref. 9.

Table 3 A comparative summary of predicted genes within each goose genome assembly.
Click here to access/download;Table;Table 3.docx

Table 3 A comparative summary of predicted genes within each goose genome assembly.

Property	This study	Lu *et al.*[a]	Gao *et al.*[b]
Total PCG length (bp)	326,863,440	439,289,059	500,923,091
PCG number	17,568	16,150	16,288
PCG percentage of genome (%)	29.34	39.25	44.31
Total exons number	152,392	158,713	167,532
Average exons per gene	8.67	10.92	10.29
Total exons length (bp)	26,883,354	25,763,242	26,157,477
Exons percentage of genome (%)	2.41	2.31	2.31
Average exons length (bp)	176.41	162.33	156.13
Average introns length (bp)	2224.97	2867.48	3139.07

[a] From the ref. 8. [b] From the ref. 9.

Figure 1 A picture of a female adult goose used for genome sequencing.

Click here to access/download;Figure;Figure1_update_1.tiff

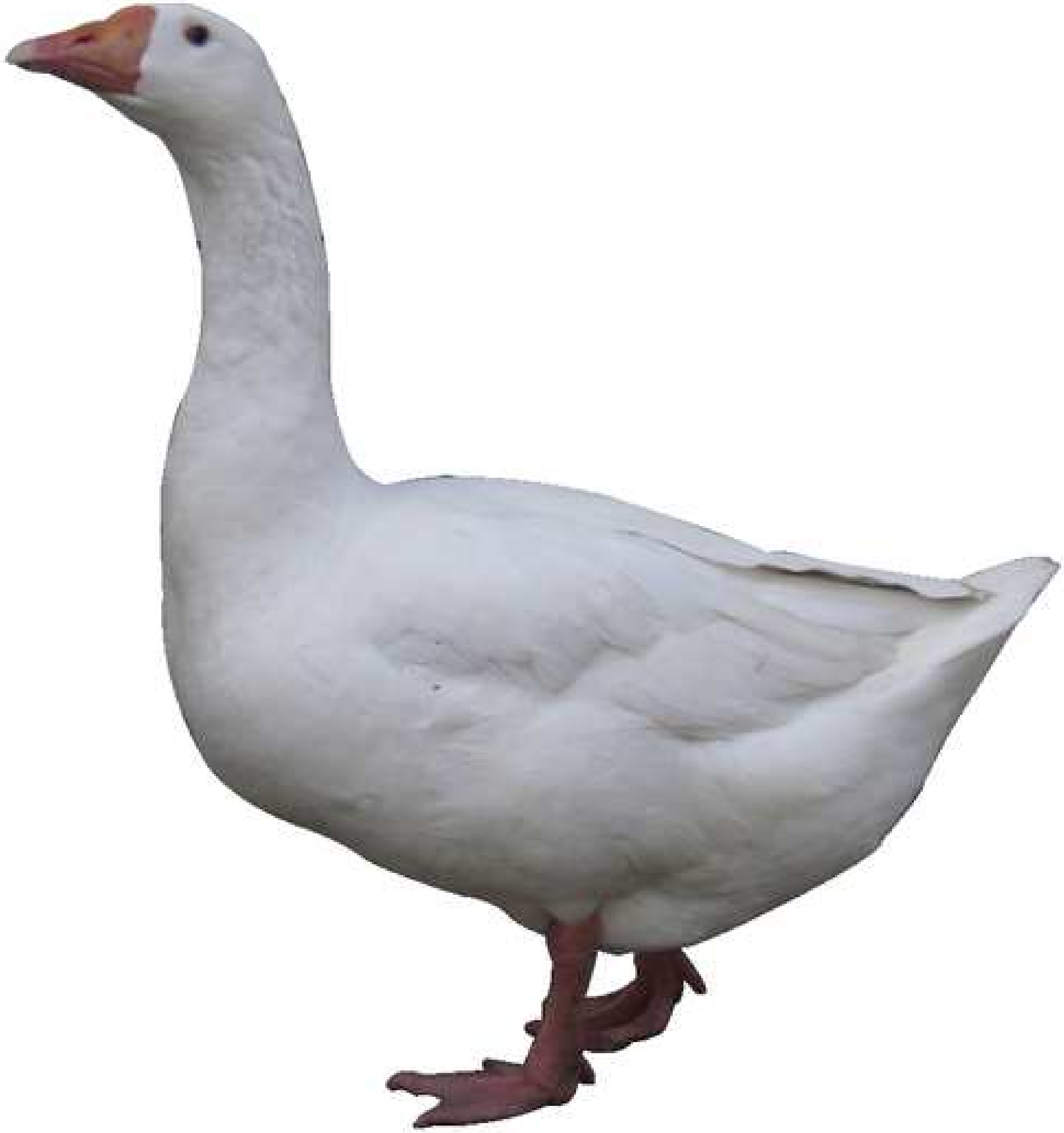

Figure 2 Comparison of the distribution and coverage of the scaffolds for the assembly with previous goose genome assemblies.

Click here to access/download;Figure;Figure 1u.tif

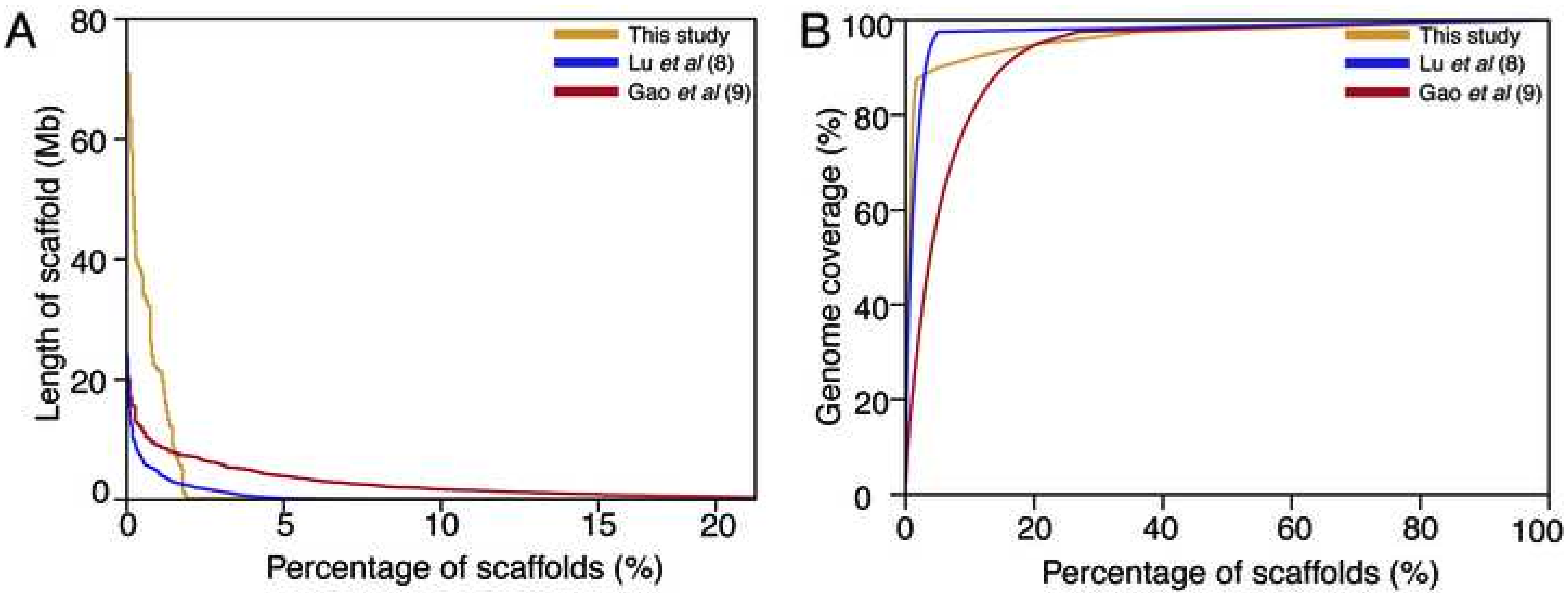

Figure 3 Dendrogram of inter-pseudo-chromosome interaction patterns generated by the average linkage algorithm.

Click here to access/download;Figure;Figure 2u.tiff

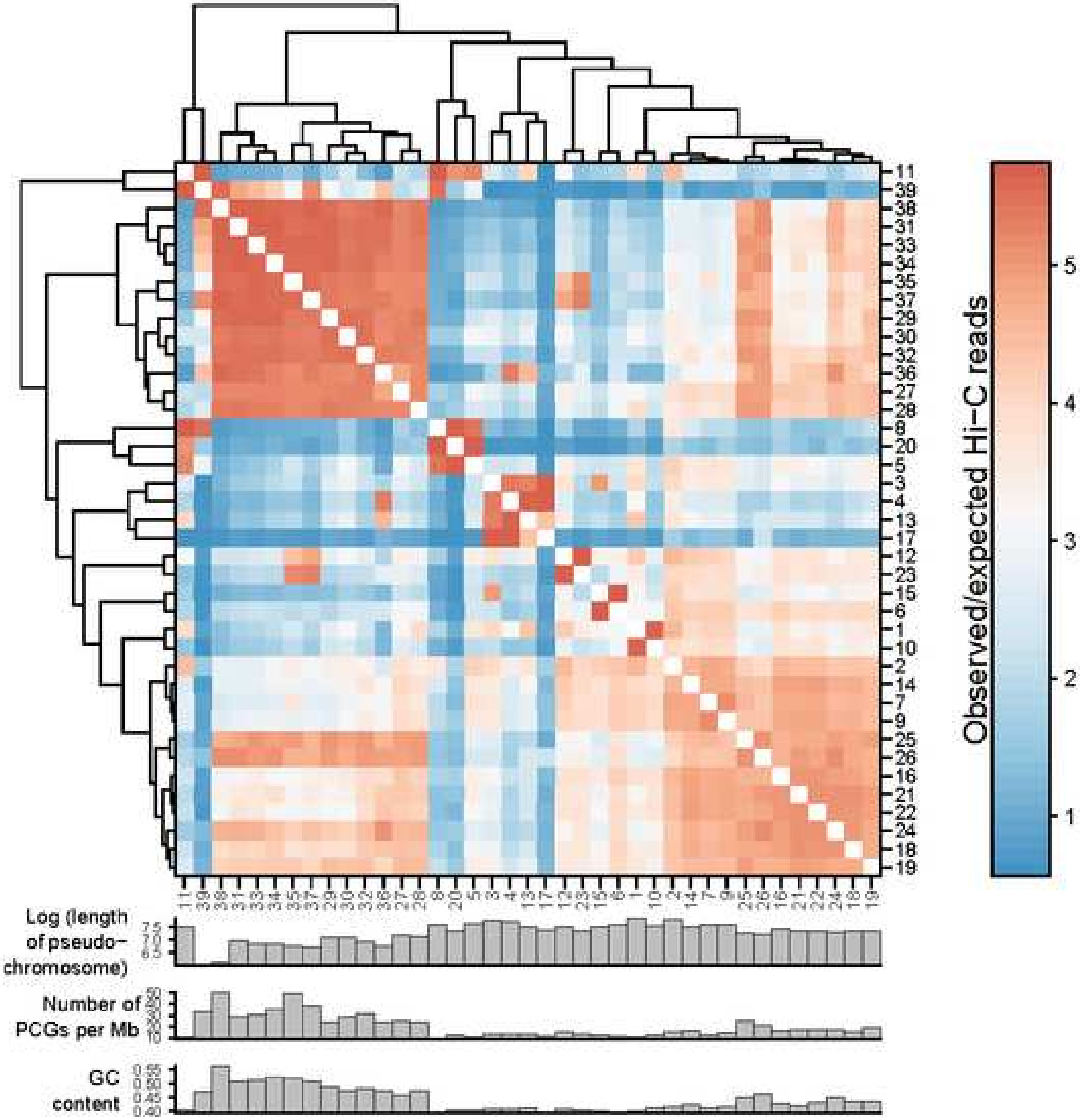

Click here to access/download
Supplementary Material
Supplymental_materials.docx

Table S1 Summary of the Pacbio initial assembly and Hi-C reads mapping used for goose genome assembly process.

Click here to access/download
Supplementary Material
Table S1.xls

Table S2 Summary of the length of pseudo-chromosomes in goose genome.

Click here to access/download
Supplementary Material
Table S2.xls

Table S3 A comparative summary of assembled repeat content between this study and previous studies.

Click here to access/download
Supplementary Material
Table S3.xls

Table S4 Comparison of the mapping rates of the wild goose resequencing data between our goose genome and two previous

Click here to access/download
Supplementary Material
Table S4.xls

Table S5 Gene ontology (GO) enrichment analysis for the lineage-specific genes annotated in goose genome.

Click here to access/download
Supplementary Material
Table S5.xls

Table S6 Functional gene categories enriched for the goose genome-specific expansion gene families.

Click here to access/download
Supplementary Material
Table S6.xls

Table S7 Functional gene categories enriched for the contraction of gene families in goose genome.

Click here to access/download
Supplementary Material
Table S7.xls

Table S8 Positively selected genes (PSGs) identified in the goose genome.

Click here to access/download
Supplementary Material
Table S8.xlsx

Table S9 The PC1 values (100 Kb) through Principal Component Analysis (PCA) and A-B index values (25 Kb).

Click here to access/download
Supplementary Material
Table S9.xlsx

Table S10 TAD in genome coordinates of our goose genome by using method of DI values.

Click here to access/download
Supplementary Material
Table S10.xlsx

Table S11 Detailed information of promoter-enhancer interactions (PEIs) identified in goose genome.

Click here to access/download
Supplementary Material
Table S11.xlsx

Figure S1 The Hi-C interaction contact heatmap of goose pseudochromosome genome assembly (bin size is 1Mb).

Click here to access/download
Supplementary Material
Figure S2u.jpg

Figure S2 The shared homologous gene families across the six species (Chicken, Goose, Human, Mouse, Pig, Zebra finch).

Click here to access/download
Supplementary Material
Figure S3u.tiff

Figure S3 The distribution of gene density in the goose genome.
Number of PCGs in each 1Mb bins was counted.

Click here to access/download
Supplementary Material
Figure s4u.tif

Figure S4 Divergence of time and the expansion, contraction gene families in the seventeen species (Ostrich, Duck, Goose, Chicken,

Click here to access/download
Supplementary Material
Figure S5.jpg

Figure S5 Resolution evaluation showing that the Hi-C data attained 2 Kb.

Click here to access/download
Supplementary Material
Figure S6.tif

Figure S6 Vioplot of PC1 values in 100 Kb bins with various number of PCGs. PC1 value indicates the chromatin activity.

Click here to access/download
Supplementary Material
Figure S7u.tif

Figure S7 TPMs of PCGs located in A compartments were consistently higher than PCGs in B compartments both at 25 Kb

Click here to access/download
Supplementary Material
Figure S8u.tif

Figure S8 TAD distribution across the goose genome assembly.

Click here to access/download
Supplementary Material
Figure S9.tif

Figure S9 TSSs of PCGs were enriched in TAD boundary regions.

Click here to access/download
Supplementary Material
Figure S10u.tif

Figure S10 Gene expression levels positively correlated with the number of its associated enhancers in all three liver tissues,

Click here to access/download
Supplementary Material
Figure S11u.tif
