# Supplementary material for: Pacific Biosciences assembly with Hi-C mapping generates an improved, chromosome-level goose genome
Source: Gigascience. 2020 Oct 24;9(10):giaa114. doi: 10.1093/gigascience/giaa114 (PMC7585555; doi:10.1093/gigascience/giaa114)
Supplement: giaa114_Supplemental_Files [file giaa114_supplemental_files.zip › Supplymental_materials.docx]

**Supplementary Methods**

**1 Goose genome assembly, annotation and the spatial organization of chromatin in liver tissues analysis by the following software:**

**1.1 Goose genome were de novo assembled by the following software:**

(1) FALCON: version 3.1, parameters: length_cutoff = 5000 length_cutoff_pr = 4500;

(2) pbsmrtpipe: version smrtlink_5.0.1, default parameters;

(3) SSPACE-LongRead: version 1-1, default parameters;

(4) PBjelly: version PBSuite_15.8.24, parameters: blasr : -minMatch 8 -minPctIdentity 75 -bestn 1 -nproc 13 -noSplitSubreads;

(5) pilon: version pilon-1.18, parameters: -Xmx400G --diploid --threads 30;

(6) Lachesis: version-201701, parameters: RE_SITE_SEQ = GATC, CLUSTER_N = 39, CLUSTER_MIN_RE_SITES = 600, CLUSTER_MAX_LINK_DENSITY = 3, CLUSTER_NONINFORMATIVE_RATIO = 0.

(7) kallisto: version 0.44.0, parameters: -i -o --bias --rf-stranded.

**1.2 Goose genome were annotated followed the software:**

(1) GCE: version1.0.0, parameters: -H 1;

(2) SOAPdenovo: version2, k-mer size of 59;

(3) GAPcloser: version1.12, parameters: -l 150 -p 31;

(4) SSPACE: version3.0, default parameters;

(5) RepeatMasker: Repeat Masker-open-4-0-6, parameters: -a -nolow -no_is -norna -parallel 1;

(6) RepeatModeler: RepeatModeler-open-1.0.11, parameters: -database genome -engine ncbi -pa 15;

(7) Tandem Repeats Finder: TRF-407b, parameters: 2 7 7 80 10 50 2000 -d -h;

(8) TBLASTN: blast-2.2.26, parameters: -e 1e-05 -F T -m 8;

(9) GeneWise: version2.4.1, parameters: -tfor/-trev -genesf -gff;

(10) Augustus: version3.2.3, param- eters: –uniqueGeneId = true–noInFrameStop = true–gff3 = on–genemodel = complete–strand = both;

(11) GlimmerHMM: version3.0.1, parameters: -g -f;

(12) SNAP: snap-2013-11-29, default parameters;

(13) Trinity: trinityrnaseq-2.1.1, parameters: –seqType fq-CPU 20–max_memory 200G–normalize_reads–full_cleanup– min_glue 2–min_kmer_cov 2–KMER_SIZE 25;

(14) PASA: PASA_r20140417, default parameters;

(15) InterPro: version29.0, perl-based version4.8, default parameters;

(16) tRNAscan-SE: tRNAscan-SE-1.3.1, default param- eters;

(17) INFERNAL: version1.1rc4 (June 2013);

(18) BLASTp: blast-2.2.26, parameters: -p blastn -e 1e-10 -v 10000 -b 10000;

(19) EVM: VidenceModeler-1.1.1, parameters: –segment- Size 200000–overlapSize 20000;

(20) Tophat: tophat-2.0.13, parameters: -p 6–max-intron-length 500000 -m 2– library-type fr-unstranded;

(21) Cufflinks: cufflinks-2.1.1, parameters: -I 500000 -p 1–library-type fr-unstranded -L CUFF;

(22) BUSCO: version3.0.2, OrthoDBv9_vertebrata;

(23) BWA: bwa-0.7.8, parameters: mem -k 32 -w 10 -B 3 -O 11 -E 4 -t 10;

(24) SAMtools: samtools-0.1.19, parameters: mpileup mpileup -m 2 -u;

(25) RAxML: version 8.0.19, default parameters;

(26) CAFÉ: Version 1.6, default parameters;

(27) BLASTP: Version 2.2.26, default parameters;

(28) PAML: Version 14.7, default parameters;

**1.3 LncRNA and TUCP were annotated followed the software:**

(1) STAR: version 2.6.0c, default parameters;

(2) Cufflinks: version 2.2.1, default parameters;

(3) TACO: version 0.7.3, parameters: --filter-min-expr 0.1 --isoform-frac 0.1 --path-kmax 20 --max-paths 20 --filter-min-length 250 --gtf-expr-attr FPKM;

(4) taco_refcomp: part of TACO in version 0.7.3, parameters: -o -r ${ref} -t

(5) CPC2: version beta of CPC2, default parameters;

(6) transeq: parts of EMBOSS in version 6.6.0, parameters: -sequence -outseq -frame 6 -clean;

(7) kallisto: version 0.44.0, parameters: -i -o --bias --rf-stranded.

**1.4 Hi-C data analysis by the following software:**

(1) Juicer: version 1.8.9, parameters: -C 8000000 -s MboI -p goose.chromosome.sizes -z goose.fa -y goose.MboI.fragment.txt -n 10G;

(2) Hi-C Domain Caller, pipeline to call domains from Hi-C experiments: http://chromosome.sdsc.edu/mouse/hi-c/download.html;

(3) PSYCHIC: parameters, res: 25000, win: 2000000, chrname: chr*, chrsize: chr*.size, output_prefix: goose.chr*.25000, output_dir: output_directory, input_matrix: goose.chr*.25000.normalized.matrix, gene_file: goose.gene.psychic.bed, skip_hierarchy: FALSE;

**2 High-throughput chromatin conformation capture (Hi-C)**

**2.1 Hi-C experiment**

Hi-C library on liver tissue of a healthy adult female (136 days old) from the Tianfu goose maternal line was performed as previously described using the MboI restriction enzyme with minor modifications ^[1]^. Library was then sequenced on the sequencing platform of BGI-seq 500.

**2.2 Hi-C data processing**

We then used an efficient open-source tool (Juicer, RRID:SCR_017226) to process Hi-C datasets ^[2]^. In brief, high quality Hi-C reads were aligned to our goose assembly using BWA-mem module (BWA, RRID:SCR_010910). After filtering abnormal alignments, duplicates and low-quality alignments (MAPQ ＜ 30), normalized contract matrix at resolution (25Kb, 100Kb) was constructed using KR algorithm.

**2.3 Identification of compartment A/B at resolution of 100 Kb and 25 Kb**

Compartment A/B analysis at 100 Kb resolution was performed using principal components analysis (PCA) as previously described ^[1]^. Briefly, by using ‘cor’ function in R, the Pearson correlation matrix was generated. Then we used the ‘prcomp’ function in R to generate principle components. Bins of 100 Kb with positive Spearman’s correlation between PC1 values and PCG number were defined as compartment A, otherwise B. Identification of compartment A/B at 25 Kb was performed by using A-B index as described previously ^[3]^.

**2.4 Identification of topologically associated domains (TADs)**

Based on the normalized contact matrix at 25 Kb, TAD was identified by using the directionality index (DI) score and a Hidden Markov Model (HMM) as previously described ^[4]^.

**2.5 Promoter-Enhancer interaction (PEI) analysis**

Based on the normalized contact matrix at 25 Kb, we used PSYCHIC software to generate raw PEIs ^[5]^. Then We filtered low confidence PEIs with interaction distance lower than 25 Kb.

**3 Gene expression quantification**

For RNA-seq data of goose liver tissue, we downloaded three RNA-seq data restricted to liver tissue (Accession number: GSM3374538, GSM3374539, GSM3374540), which was from the same goose strain of our study ^[6]^. The three high-quality datasets were aligned to our goose assembly using STAR (STAR, RRID:SCR_015899) ^[7]^. Gene-level expression was estimated as transcripts per million (TPM) using the high-speed transcript quantification tool Kallisto (Kallisto, RRID:SCR_016582) ^[8]^.

**3. References**

1. Lieberman-Aiden E, van Berkum NL, Williams L, et al. Comprehensive mapping of long-range interactions reveals folding principles of the human genome. Science. 2009; 326(5950): 289-293.

2. Rao SS, Huntley MH, Durand NC, et al. A 3D map of the human genome at kilobase resolution reveals principles of chromatin looping. Cell. 2014; 159(7): 1665-1680.

3. Rowley MJ, Nichols MH, Lyu X, et al. Evolutionarily conserved principles predict 3D chromatin organization. Mol Cell. 2017; 67(5): 837-852.

4. Dixon JR, Selvaraj S, Yue F, et al. Topological domains in mammalian genomes identified by analysis of chromatin interactions. Nature. 2012; 485(7398): 376-380.

5. Ron G, Globerson Y, Moran D, et al. Promoter-enhancer interactions identified from Hi-C data using probabilistic models and hierarchical topological domains. Nat Commun. 2017; 8(1): 2237.

6. Wang G, Jin L, Li Y, et al. Transcriptomic analysis between normal and high-intake feeding geese provides insight into adipose deposition and susceptibility to fatty liver in migratory birds. BMC Genomics. 2019; 20(1): 372.

7. Dobin A, Davis CA, Schlesinger F, et al. STAR: ultrafast universal RNA-seq aligner. Bioinformatics. 2013; 29(1): 15-21.

8. Bray NL, Pimentel H, Melsted P, et al. Near-optimal probabilistic RNA-seq quantification. Nat Biotechnol. 2016; 34(5): 525-527.
