# Supplementary figures and images for: Pacific Biosciences assembly with Hi-C mapping generates an improved, chromosome-level goose genome
Source: Gigascience. 2020 Oct 24;9(10):giaa114. doi: 10.1093/gigascience/giaa114 (PMC7585555; doi:10.1093/gigascience/giaa114)

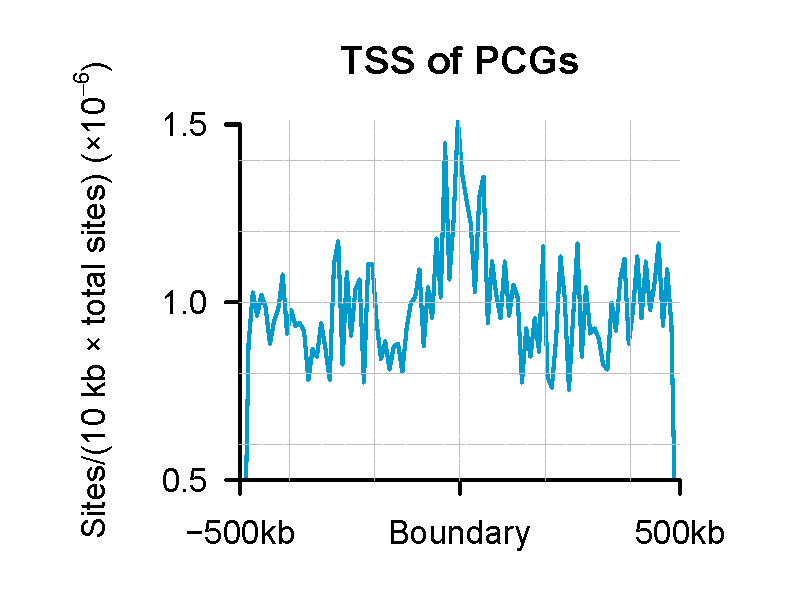

Supplement: giaa114_Supplemental_Files [file giaa114_supplemental_files.zip › Figure S10u.tif]

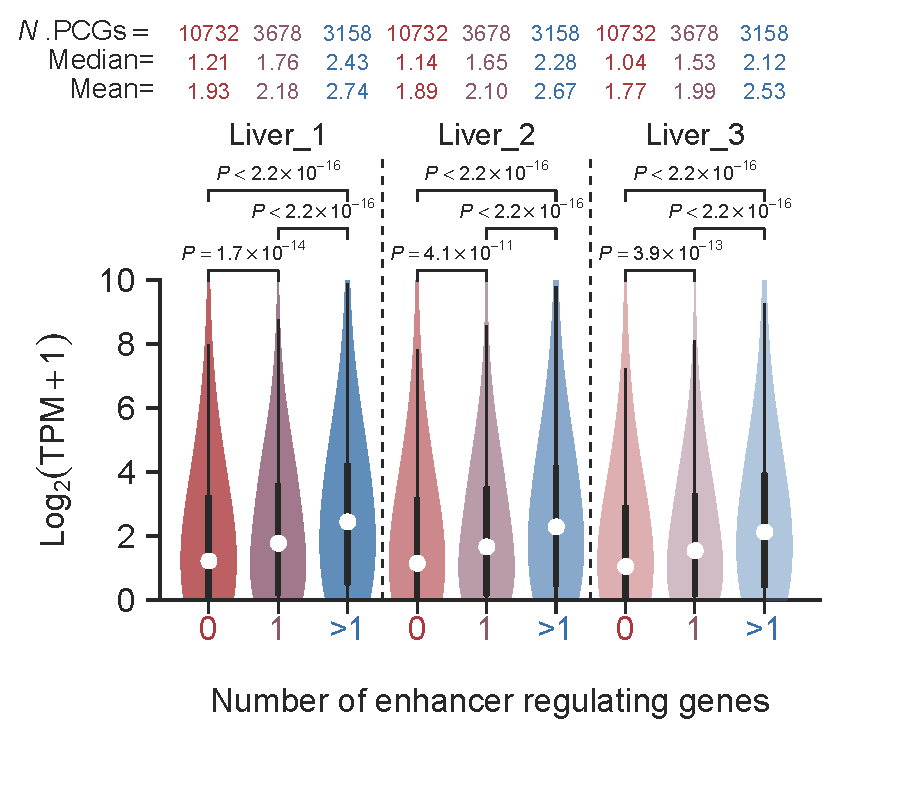

Supplement: giaa114_Supplemental_Files [file giaa114_supplemental_files.zip › Figure S11u.tif]

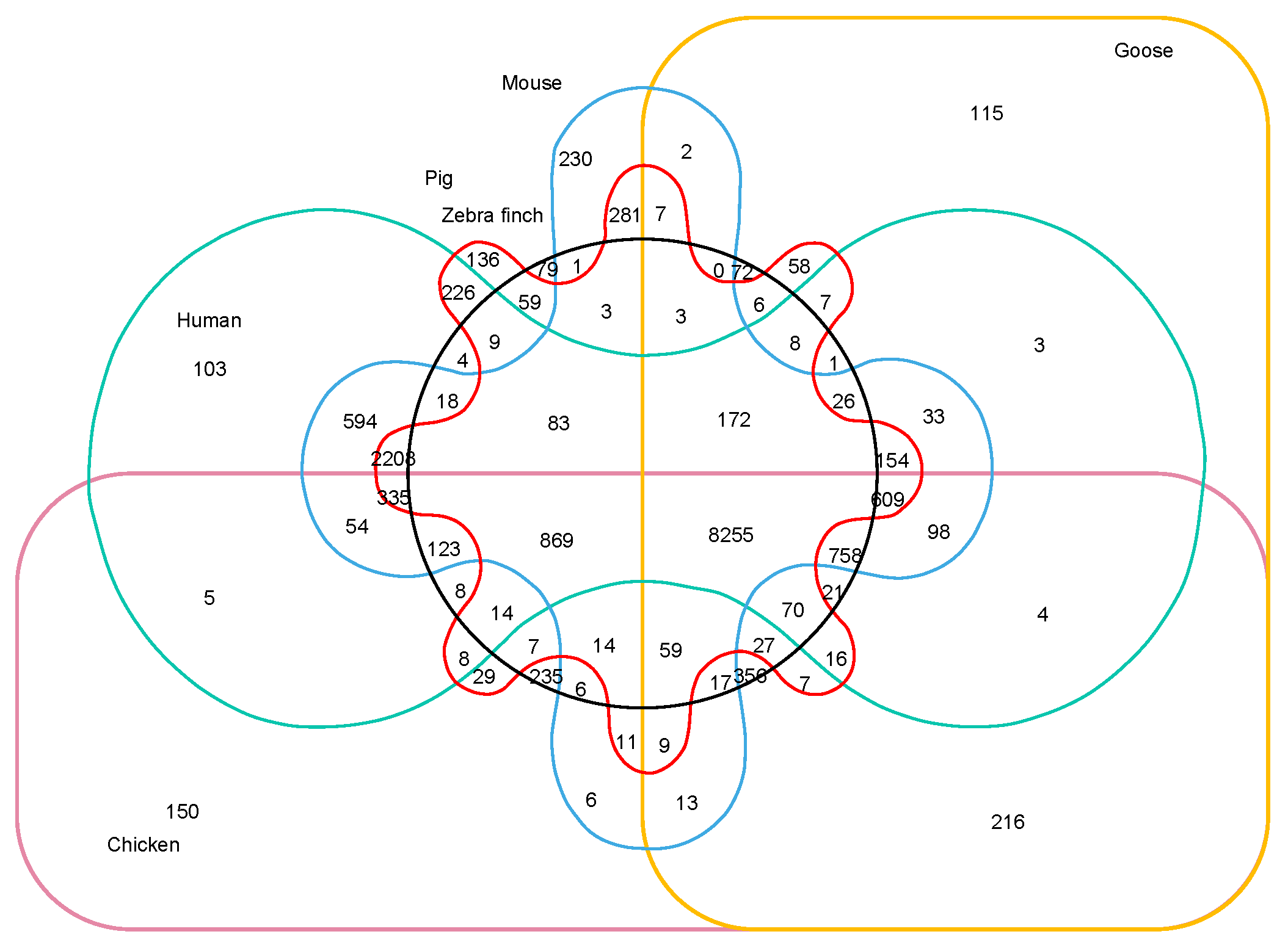

Supplement: giaa114_Supplemental_Files [file giaa114_supplemental_files.zip › Figure S3u.tiff]

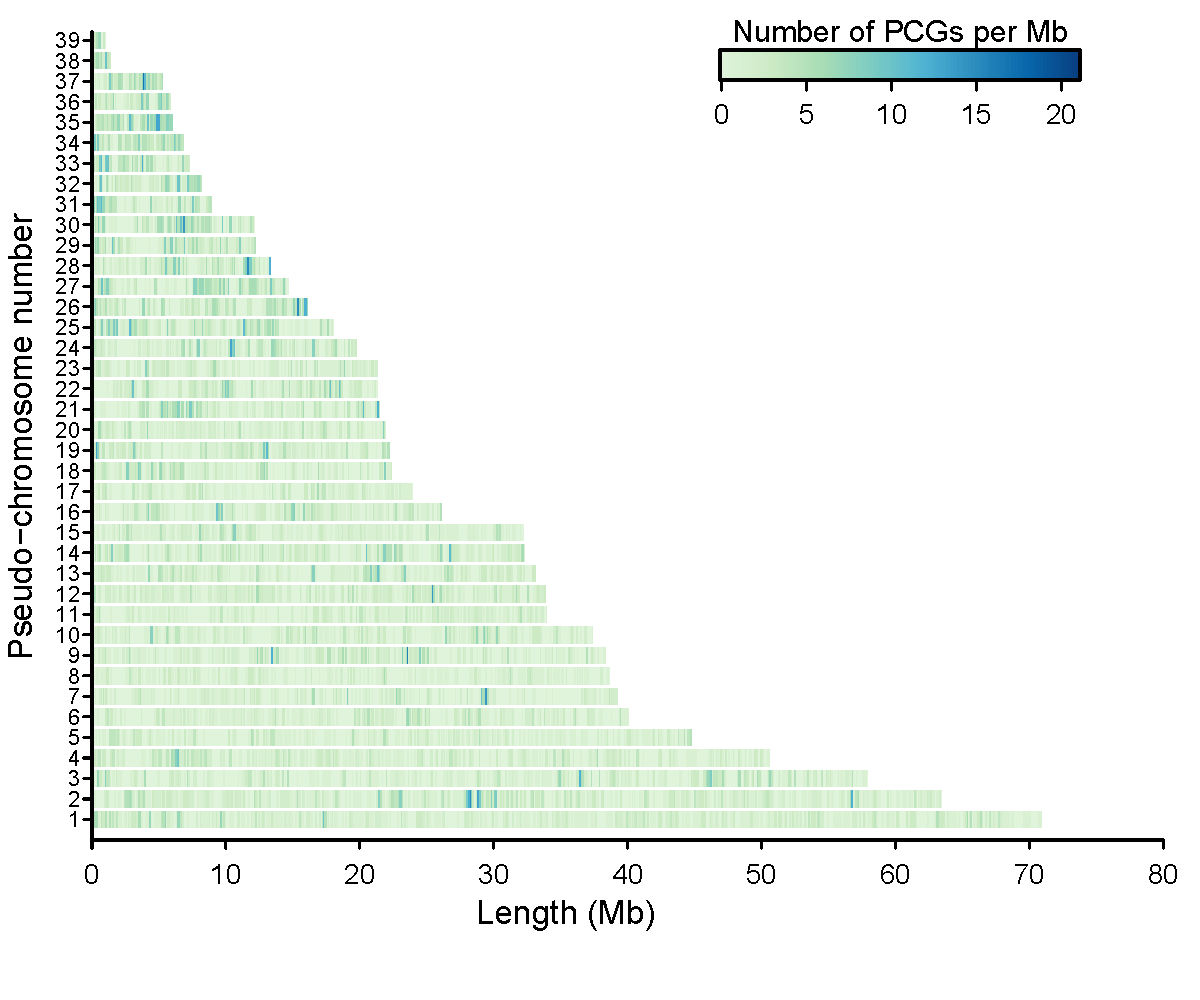

Supplement: giaa114_Supplemental_Files [file giaa114_supplemental_files.zip › Figure s4u.tif]

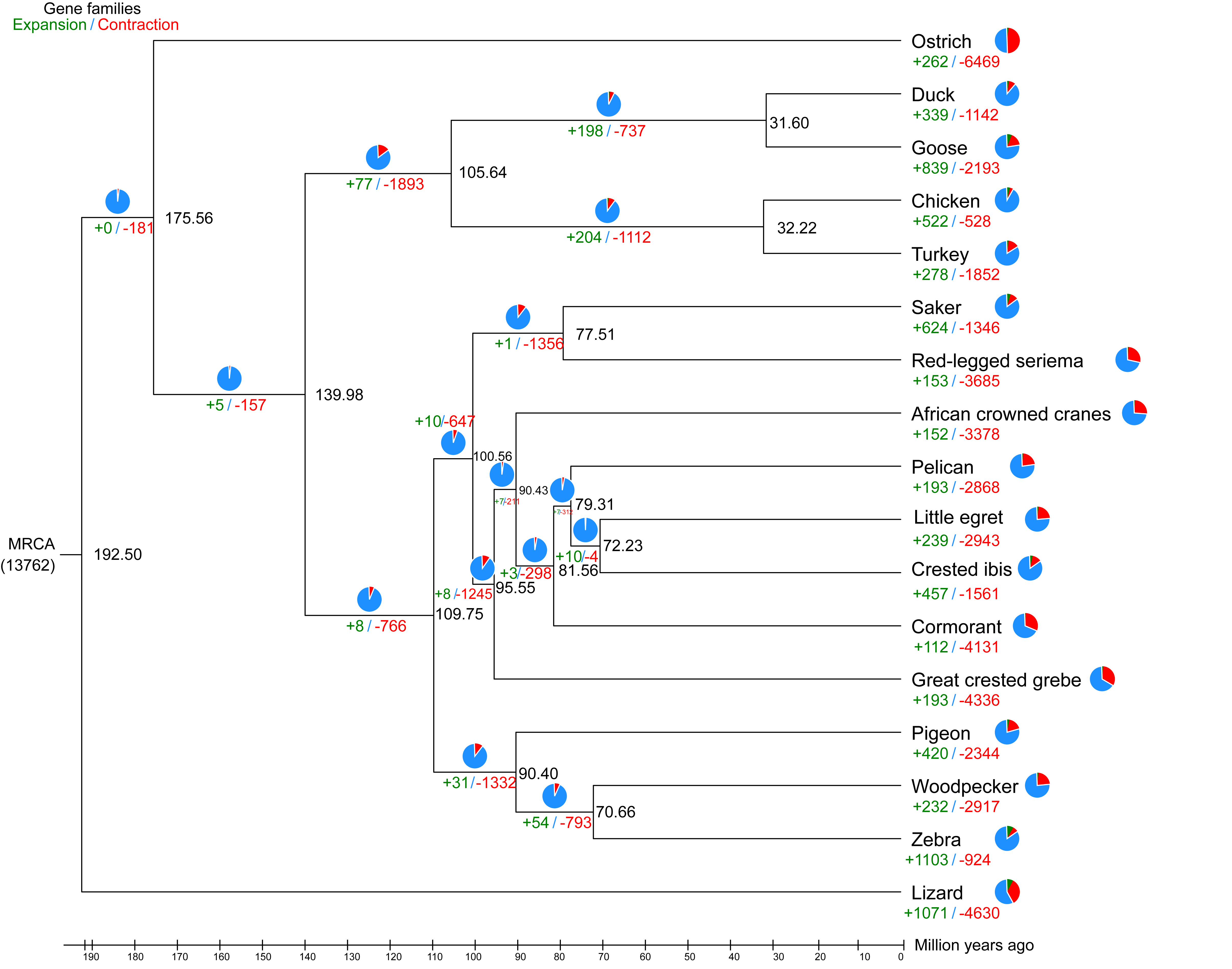

Supplement: giaa114_Supplemental_Files [file giaa114_supplemental_files.zip › Figure S5.jpg]

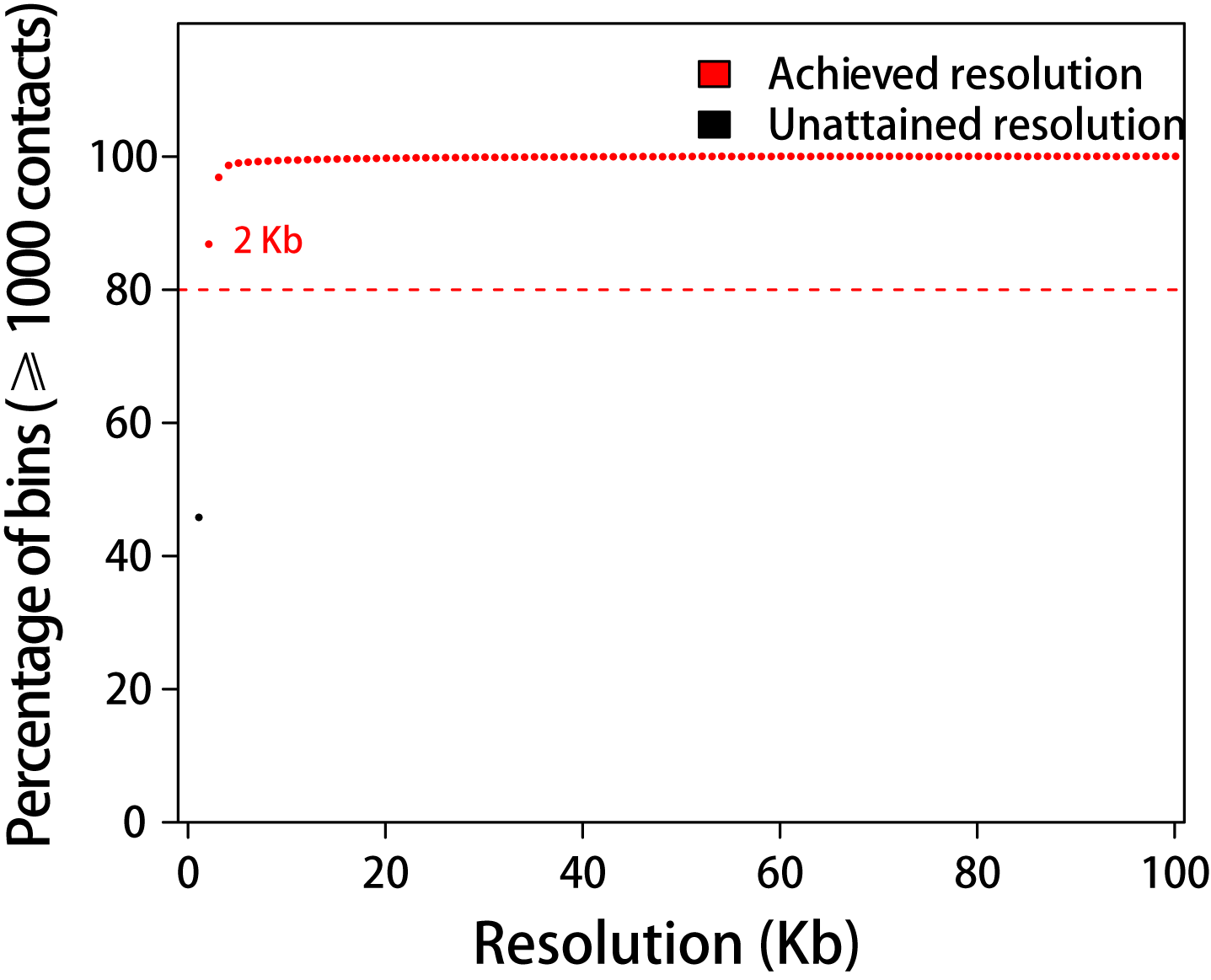

Supplement: giaa114_Supplemental_Files [file giaa114_supplemental_files.zip › Figure S6.tif]

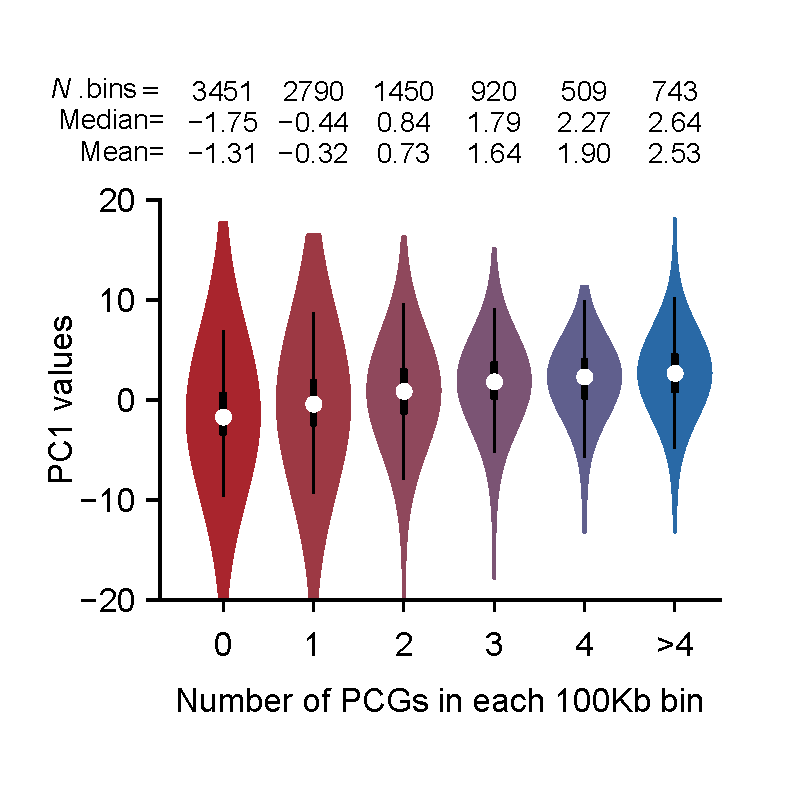

Supplement: giaa114_Supplemental_Files [file giaa114_supplemental_files.zip › Figure S7u.tif]

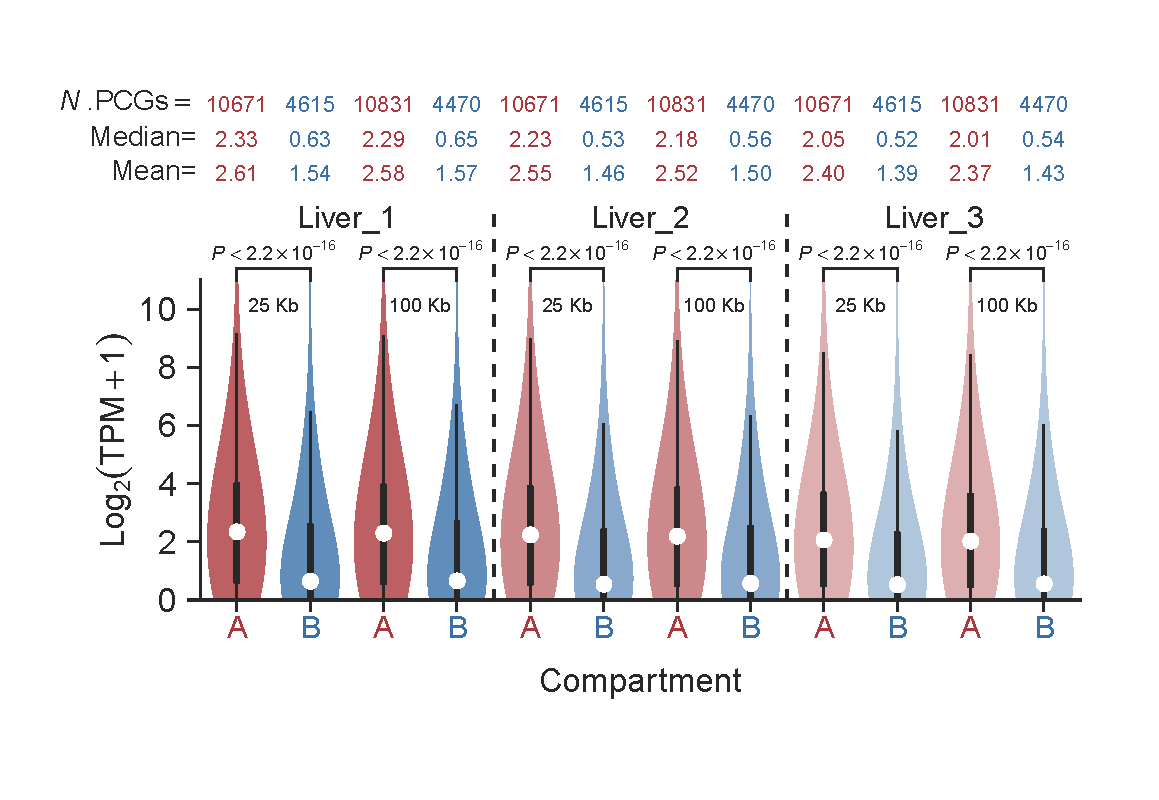

Supplement: giaa114_Supplemental_Files [file giaa114_supplemental_files.zip › Figure S8u.tif]

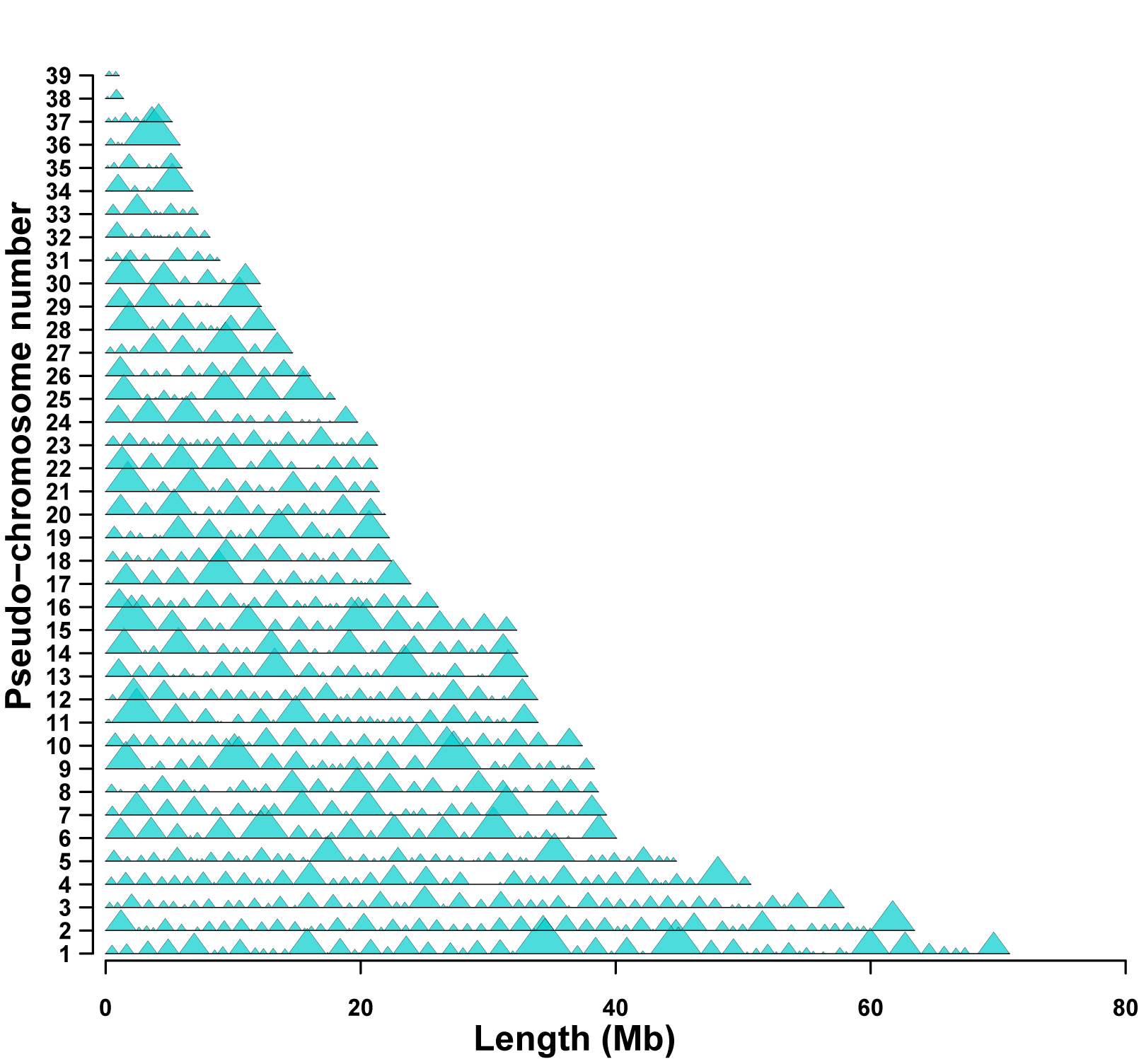

Supplement: giaa114_Supplemental_Files [file giaa114_supplemental_files.zip › Figure S9.tif]
