# Supplementary material for: Pacific Biosciences assembly with Hi-C mapping generates an improved, chromosome-level goose genome
Source: Gigascience. 2020 Oct 24;9(10):giaa114. doi: 10.1093/gigascience/giaa114 (PMC7585555; doi:10.1093/gigascience/giaa114)
Supplement: giaa114_Response_to_Reviewer_Comments_Original_Submission [file giaa114_response_to_reviewer_comments_original_submission.pdf]

Author's Response To Reviewer Comments

Close

Detailed responses to Reviewers
Below, all critiques and suggestions provided by the reviewers are cited in gray italics; our responses are in black. In red a
descriptions within the responses that indicate changes in the manuscript. Moreover, all revisions in the manuscript are m
red.

____

Reviewer 1
This paper reports on the assembly and annotation of the Tianfu goose genome, a female hybrid of A. anser x A. cygnoide
assembly is a significant improvement on earlier genomes, which were based on short read technologies. This assembly is
of three technologies: short reads, long reads and HiC maps.

Comment 1:
MAJOR POINTS
1. The assignment of 39 chromosomes to Hi-C scaffolds is very tentative and needs to be validated. For larger scaffold yo
establish homology e.g. with chicken chromosomes, which have extensive FISH/cytogenetic data at least for the
macrochromosomes. The smaller scaffolds in the HiC analysis could be parts of larger chromosomes - the HiC map sugges
mis-joins. Also in other genome projects very GC-rich, repeat-rich chromosomes (such as microchromosomes) are difficul
impossible to sequence, and are missing from the assembly. So 39 pseudo-chromosomes are found but these do not equa
39 physical chromosomes. This affects conclusions on chromosome number, genome completeness, gene density distribut
distribution of TADs, etc. As a reference goose genome these points need to be addressed.

Response 1：
Thank you for this valuable suggestion. We recognize the importance of a reference genome that comprises accurate, phy
chromosomes for future genetic and genomic studies on geese. In this study, we generated a 1.11 Gb goose genome with
and scaffold N50 values of 1.85 Mb and 33.12 Mb, respectively (Table 1). Our assembly contains 39 pseudo-chromosomes
78), which account for 88.36% of the goose genome; it is a draft goose genome assembly rather than a complete assemb
physical chromosomes. As far as we know, this genome is comparable with other chromosome-level avian genome assem
(Table 1). (The tables and figures were accessible from Response_sup.pdf at: https://fnca1-my.sharepoint.com/:b:/g/personal/guanglianggaocq_lwh_world/EQSVOZIBkJ9HlaxEnZFufdcBf_eOaki7d4jJkNUz9Yrwpw?e
We regret that our original description implied a complete genome assembly of 39 chromosomes. We have corrected this
stating throughout the manuscript that our assembly is a chromosome-level goose genome assembly comprising 39 pseud
chromosomes.

OTHER POINTS
Comment 1-1:
1. This is a chromosome-level assembly make this clear in the text.

Response 1-1:
In accordance with Reviewer 1's suggestion, we have clarified that it is a chromosome-level assembly throughout the man

Comment 1-2:
2. The hybrid approach used here is good but this is a rapidly evolving field, and is already superseded by technology (Pac
now so polishing using short reads not needed) and software (e.g. Lachesis no longer supported).

Response 1-2:
Thank you for this valuable insight. Certainly, de novo whole-genome assembly approaches change over time, and algorit
adapt in line with evolving sequencing technologies. This allows researchers to generate more continuous, complete, and a
genome assemblies. To facilitate genomic, genetic, and breeding studies on the goose, we report here an improved, chror
level goose genome for the scientific community. We expect that our current goose assembly and annotation will be helpf
researchers in different study fields. Furthermore, we remain committed to assembling a complete and accurate goose ge
sequence in the future. For this purpose, we plan to adopt the Pacbio HiFi and Pacbio isoseq technologies, and combine th
extensive FISH and cytogenetic experiments, and genetic map data.
Regarding the three software packages, LACHESIS, SALSA2, and 3D-DNA, each have advantages and limitations for de no
genome assembly. Namely, (1) while we employed the LACHESIS software to combine shotgun fragments and short jump
pair sequences with Hi-C data (to generate chromosome-scale de novo genome assemblies), LACHESIS has limitations wh
assembling polyploid genomes [1]. (2) The SALSA2 algorithm does not require that the number of chromosomes are set i
advance, which improves the accuracy of scaffolds to a certain extent; however, this algorithm can introduce many
clustering/sorting/orientation errors, and few parameters can be adjusted during operation [2]. (3) 3D-DNA corrects error

input assembly and then iteratively orients and orders contigs into a single mega-scaffold. This mega-scaffold is then brok
chromosomal ends are identified based on a Hi-C contact map. A drawback is that the error correction function in this soft
not been well applied; in the case of simulated data, the assembly error rate of 3D-DNA is 2–4 times that of SALSA2 [2].
To choose an appropriate software for our genome assembly, we randomly selected a subset of our Hi-C data and perform
novo genome assembly using SALSA2, 3D-DNA, and LACHESIS. As the quality metrics of the LACHESIS genome assembli
the best, we performed the subsequent studies in this paper with the LACHESIS software.

Comment 1-3:
3. The phrase "high-quality" is used throughout the text but not defined - so please define. It is more likely that sequence
generated (provide QC data on quality) and then software is used to filter out poor data, to leave high-quality data for ass
Response 1-3
Thank you for this useful insight. In our manuscript, the phrase "high-quality" refers to data that was filtered from three sequencing platforms and used for the genome assembly. For the short reads, we employed a Perl script written by our la
the data from the Illumina platform. As a result, the Q20 and Q30 values of the whole genome sequencing data from the
platform were greater than 96.44 % and 93.25 %, respectively (Table 2). (accessible from Response_sup.pdf at: https://
my.sharepoint.com/:b:/g/personal/guangliangaocq_lwh_world/EQSVOZIBkJ9HlaxEnZFufdcBf_eOaki7d4jJkNUz9Yrwpw?e
The Q20 and Q30 values of the Hi-C data used in our study were 97.86 % and 91.84 %, respectively (Table 2). These res
suggest that the data used in our genome assembly were "high-quality".

Comment 1-4:
4. For all software, please provide versions and source.
Response 1-4:
Following Reviewer 1's valuable suggestion, we have added the below descriptions to the supplemental materials (see line
Goose genome assembly, annotation and the spatial organization of chromatin in liver tissues analysis by the following so
Goose genome were de novo assembled by the following software:
(1) FALCON: version 3.1, parameters: length_cutoff = 5000 length_cutoff_pr = 4500;
(2) pbsmrtpipe: version smrtlink_5.0.1, default parameters;
(3) SSPACE-LongRead: version 1-1, default parameters;
(4) PBjelly: version PBSuite_15.8.24, parameters: blasr: -minMatch 8 -minPctIdentity 75 -bestn 1 -nproc 13 -noSplitSubr
(5) pilon: version pilon-1.18, parameters: -Xmx400G --diploid --threads 30;
(6) Lachesis: version-201701, parameters: RE_SITE_SEQ = GATC, CLUSTER_N = 39, CLUSTER_MIN_RE_SITES = 600, CLUSTER_MAX_LINK_DENSITY = 3, CLUSTER_NONINFORMATIVE_RATIO = 0.
(7) kallisto: version 0.44.0, parameters: -i -o --bias --rf-stranded.
Goose genome were annotated followed the software:
(1) GCE: version1.0.0, parameters: -H 1;
(2) SOAPdenovo: version2, k-mer size of 59;
(3) GAPcloser: version1.12, parameters: -l 150 -p 31;
(4) SSPACE: version3.0, default parameters;
(5) RepeatMasker: Repeat Masker-open-4-0-6, parameters: -a -nolow -no_is -norna -parallel 1;
(6) RepeatModeler: RepeatModeler-open-1.0.11, parameters: -database genome -engine ncbi -pa 15;
(7) Tandem Repeats Finder: TRF-407b, parameters: 2 7 7 80 10 50 2000 -d -h;
(8) TBLASTN: blast-2.2.26, parameters: -e 1e-05 -F T -m 8;
(9) GeneWise: version2.4.1, parameters: -tfor/-trev -genesf -gff;
(10) Augustus: version3.2.3, param- eters: –uniqueGeneId = true–noInFrameStop = true–gff3 = on–genemodel = compl
strand = both;
(11) GlimmerHMM: version3.0.1, parameters: -g -f;
(12) SNAP: snap-2013-11-29, default parameters;
(13) Trinity: trinityrnaseq-2.1.1, parameters: –seqType fq-CPU 20–max_memory 200G–normalize_reads–full_cleanup– m
2–min_kmer_cov 2–KMER_SIZE 25;
(14) PASA: PASA_r20140417, default parameters;
(15) InterPro: version29.0, perl-based version4.8, default parameters;
(16) tRNAscan-SE: tRNAscan-SE-1.3.1, default param- eters;
(17) INFERNAL: version1.1rc4 (June 2013);
(18) BLASTp: blast-2.2.26, parameters: -p blastn -e 1e-10 -v 10000 -b 10000;
(19) EVM: VidenceModeler-1.1.1, parameters: –segment- Size 200000–overlapSize 20000;
(20) Tophat: tophat-2.0.13, parameters: -p 6–max-intron-length 500000 -m 2– library-type fr-unstranded;
(21) Cufflinks: cufflinks-2.1.1, parameters: -I 500000 -p 1–library-type fr-unstranded -L CUFF;
(22) BUSCO: version3.0.2, OrthoDBv9_vertebrata;
(23) BWA: bwa-0.7.8, parameters: mem -k 32 -w 10 -B 3 -O 11 -E 4 -t 10;
(24) SAMtools: samtools-0.1.19, parameters: mpileup mpileup -m 2 -u;
(25) RAxML: version 8.0.19, default parameters;
(26) CAFÉ: Version 1.6, default parameters;

(27) BLASTP: Version 2.2.26, default parameters;
(28) PAML: Version 14.7, default parameters;
LncRNA and TUCP were annotated followed the software:
(1) STAR: version 2.6.0c, default parameters;
(2) Cufflinks: version 2.2.1, default parameters;
(3) TACO: version 0.7.3, parameters: --filter-min-expr 0.1 --isoform-frac 0.1 --path-kmax 20 --max-paths 20 --filter-min 250 --gtf-expr-attr FPKM;
(4) taco_refcomp: part of TACO in version 0.7.3, parameters: -o -r -t
(5) CPC2: version beta of CPC2, default parameters;
(6) transeq: parts of EMBOSS in version 6.6.0, parameters: -sequence -outseq -frame 6 -clean;
(7) kallisto: version 0.44.0, parameters: -i -o --bias --rf-stranded.
Hi-C data analysis by the following software:
(1) Juicer: version 1.8.9, parameters: -C 8000000 -s MboI -p goose.chromosome.sizes -z goose.fa -y goose.MboI.fragme 10G;
(2) Hi-C Domain Caller, pipeline to call domains from Hi-C experiments: http://chromosome.sdsc.edu/mouse/hi-c/downlo
(3) PSYCHIC: parameters, res: 25000, win: 2000000, chrname: chr*, chrsize: chr*.size, output_prefix: goose.chr*.2500 output_dir: output_directory, input_matrix: goose.chr*.25000.normalized.matrix, gene_file: goose.gene.psychic.bed, skip_hierarchy: FALSE.

Comment 1-5:
5. LINE 84: k-mer distribution analysis used to estimate genome size - provide reference, software, method - also mentio QC estimates (repeats, polyploidy etc).
Response 1-5:
Thank you for this useful comment. To estimate genome size, repeat regions, heterozygosity, and polyploidy prior to asse the goose genome, we employed survey software based on K-mer (k = 17) frequency distributions. This predicted the ge size, repeat ratio, and heterozygosity to be 1277.1 Mb, 39.8%, and 0.4%, respectively. In the K-mer analysis, the goose demonstrated a distribution typical of a diploid genome (Figure 1), (accessible from Response_sup.pdf at: https://fnca1-my.sharepoint.com/:b:/g/personal/guanglianggaocq_lwh_world/EQSVOZIBkJ9HlaxEnZFufdcBf_eOaki7d4jJkNUz9Yrwpw?e showing only a single major peak—which could be used to estimate the genome size. In addition, the first minor peak rep the level of heterozygosity and the second minor peak represents the level of repeat sequence [3].

Comment 1-6:
6. LINE 91: Lachesis old software no longer supported - why not used SALSA2 or 3D-DNA?
Response 1-6:
Thank you for raising this important point. Please see Response 1-2 for a discussion of this issue.

Comment 1-7:
7. Figure S1: Hi-C map suggests lots of mis-joins, have you checked and manually corrected?
Response 1-7:
Thank you for this valuable comment. In accordance with this point, we also identified mis-joins in the Hi-C map, which s that these regions of the genome might be repetitive, GC-rich, or contain structural variation. As mentioned above, we ar committed to assembling a complete and accurate goose genome sequence, and in future work aim to focus on these "mi using the latest technologies and corresponding assembly algorithms.

Comment 1-8:
8. LINES 109-111, again used the term "high-quality" for a mix of genomes, Human, Mouse, Chicken probably but duck, and zebra finch are draft and not high-quality genomes.
Response 1-8:
Thank you for this insight. We have changed "high-quality" to "chromosome-level" in line 109. As described in Table 1, wh contig N50 values of zebra finch, duck, and turkey are 12.0 Mb, 36.80 Kb, and 27 Kb, respectively, these genomes were into chromosome-level assemblies with the aid of other technologies, such as RH mapping and FISH (Table 1).

Comment 1-9:
9. LINES 114-117, pooled RNAseq used, so how can you quantify gene expression later in paper? Needs deconvolution of samples - was this done? For annotation Pacbio isoseq would be better.
Response 1-9:
RNA-seq prediction is a commonly used method for improving genome annotation, correcting predicted gene structures, new alternative splicing isoforms, and discovering new genes and transcripts. In this updated manuscript, we used data fr pooled RNA-seq analysis (abdominal fat, brain, duodenum, heart, liver, lung, muscular stomach, ovary, pancreas, pector and spleen) only for the annotation of the goose genome and not for gene expression quantification or for the spatial anal chromatin organization. Accordingly, we did not perform a deconvolution analysis of the pooled RNA-seq sample.
Nevertheless, we sincerely thank Reviewer 1 for this reminder on the correct usage of pooled RNA-seq data. We have now

that our pooled RNA-seq data were inaccurately used to explore the spatial organization of chromatin in goose liver tissue address this, we downloaded three RNA-seq datasets from liver tissues (Accession numbers: GSM3374538, GSM3374539, GSM3374540), which had been generated from the same goose strain used in our study [4]. We then quantified gene exp these three samples and used these results to update all the content related to gene expression in our study, in terms of compartments and promoter-enhancer interactions. We have revised the main text and Figures S7–S11 and Figure 2 acco Regarding Pacbio, we also thank Reviewer 1 for raising this point. Certainly, the long reads from the Pacbio isoseq platfor better annotate complete transcripts in genomes. We aim to adopt this method, and other newly developed methods, whe update the quality of the goose genome assembly or annotation in future work.

Comment 1-10:
10. Prediction of lncRNAs from assembly of short read RNA-seq is known to be poor, so LINEs 121-124, where 3,287 lncR predicted needs to be taken with caution.
Response 1-10:
Thank you for this valuable suggestion. We agree that longer reads from PacBio and ONT offer advantages for resolving c features in transcriptomes when compared to short read RNA-seq. For example, read length is the major advantage of Iso cDNA transcript sequencing and Oxford Nanopore direct RNA-seq, which can both capture entire transcripts.
Compared with mRNAs, which can be annotated by a combination of ab initio and homologous assembly approaches, lncR not conserved among species. lncRNAs can thus only be identified by transcript data, without the aid of homology. Long r be helpful for the identification and annotation of lncRNAs, and in future work, we will adopt this strategy. In this study, w identified lncRNAs by analyzing the transcript data from short read RNA-seq only, and we have clarified this point in lines of the main text.

Comment 1-11:
11. LINE 160, goose and duck diverged 32 Mya, how does this estimate compare with other data sources?
Response 1-11:
Thank you for this important comment. In this study, we first downloaded the reported divergence times between each pa species (e.g. chicken and turkey) from the TimeTree website (http://www.timetree.org/). These divergence times are esti the basis of single-copy gene families via a Bayesian algorithm called mcmctree, within the software "PAML (http://abacus.gene.ucl.ac.uk/software/paml.html)". We also used well-established divergence times to further adjust the estimated divergence times of other species and improve the accuracy of our results. The divergence times predicted in o were consistent with two previous reports: 20.8 (12.9-32.7) Mya in Lu et al. [5]; and 30.0 (21.4-38.6) Mya in Gao et al. [

Comment 1-12:
12. sections (b-d) interesting predictions from phylogenetic analyses, but all speculation, there is no other data provided t these predictions.
Response 1-12:
Thank you for raising this point. The purpose of our article was to supply a valuable resource for future genetic and genon studies on geese. Accordingly, we endeavored to explore the general characteristics of the goose genome by performing c general analyses—such as the expansion and contraction of gene families, and the identification of genes under positive s In the future, experiments on gene function will help to resolve the speculations and predictions we have presented here. revised the main text in lines 171, 191-192 and 198–211 to address this issue.

Comment 1-13:
13. LINE 192, PAML Codeml analysis is crude, and does not correct for multiple testing, with 17K genes tested there is a h positive rate, was there any correction for multiple testing, if not please correct.
Response 1-13:
Thank you for this valuable comment. In our work, only single-copy genes (n = 2389) were used for the identification of g under positive selection. We did not use all 17K genes. After we calculated the p-value for each of the candidate positively genes using PAML, we further adjusted the p-values (e.g. using the Bonferroni method, a method for multiple testing) to r false-positive results.

Comment 1-14:
14. LINE 202, the TAD analysis is restricted to liver tissue.
Response 1-14:
Thank you for raising this point. We explored the spatial organization of chromatin and gene expression in goose liver tiss with regard to topologically associating domains (TAD). TADs were largely invariable across tissues or species. We have cl this point in lines 29, 213, 224, and 229.

Comment 1-15:
15. LINES 203-204, macs and mics form sub-domains in the nucleus. Figure 4 needs more explanation, poor figure.
Response 1-15:
We have replotted Figure 4 (named Figure 2 in revised manuscript) and increased the resolution of this figure. We have a

additional explanation and changed the figure legend as follows: "Dendrogram of inter-pseudo-chromosome interaction pa
generated by the average linkage algorithm. Heatmap shows the inter-pseudo-chromosome interaction probability, as ger
calculating the observed/expected contact frequencies for chromosome pair i, j. This is overlaid on a histogram showing p
chromosome length, number of protein-coding genes, and GC percentage".

Comment 1-16:
16. LINE 205, define compartments A and B, how are these defined in Hi-C data?
Response 1-16:
Thank you for this valuable query. We have now added an explanation of the methods used for identifying compartments
as well as the methods for how the spatial organization of chromatin and gene expression were explored in the goose liver
(see lines 64–92 in the supplemental materials). These methods relate to inter-pseudo-chromosomal interaction patterns,
topologically associating domains, promoter-enhancer interactions, and gene expression quantification.

Comment 1-17:
17. LINE 206, how were TSS (transcription start sites, not defined in the set of abbreviations, please add) defined? I assu
on the pooled short read RNA-seq data. If correct, this is a poor data set, since the assembly of transcripts based on short
data only defines the most 5' RNA sequenced. So misses any internal TSS, does not correct for degraded RNA, etc.
Response 1-17:
Thank you for this comment. We agree that basing the definition of TSS sites on short read data would be inaccurate. We
for the ambiguous TSS-related description in our manuscript. We have changed the description in line 221 to 223: "the nu
protein-coding genes (PCGs) in each 100-Kb bin with at least 50% percentage overlapped with a gene was counted. The n
PCGs was significantly correlated with PC1 values".

Comment 1-18:
18. LINE 213, gene expression levels based on pooled RNAseq data is a very poor dataset, should deconvolute or at least
high-quality liver RNA set.
Response 1-18:
As described above (see Response 1-9), to address this issue we downloaded three additional RNA-seq datasets that were
restricted to liver tissue (Accession numbers: GSM3374538, GSM3374539, GSM3374540). These datasets derive from the
goose strain as used in our study, and on the basis of a new analysis of these data we have updated all the sections of ou
manuscript related to gene expression. Specifically, we have changed the following description in lines 223–225: "the tran
per kilobase millions (TPMs) of PCGs located in A compartments were significantly higher than those in B compartments",
transcripts per kilobase millions (TPMs) of PCGs located in A compartments were consistently higher than PCGs in B comp
in three liver tissues". We have also changed lines 229–230 from: "found that gene expression levels positively correlated
number of PEIs", to: "found that gene expression levels positively correlated with the number of associated enhancers in a
liver tissues".

Reviewer 2
The manuscript describes a highly contiguous genome assembly of the goose genome and provides a significant improven
the assembly of this bird. The results are described very clearly, and the data has been made publicly available. The analy
are rather straightforward, and much more could have done with the interesting data generated in this study, which to me
missed opportunity.
The authors decide to sequence an F1 animal that is a cross between A. anser and A. cygnoides. I wonder why the author
use Illumina sequencing to sequence the genome of the two parents. This would have allowed the generation of two haplo
specific assemblies and the Comparison between the genomes of these two different sub-species. Also, no indication is giv
the number of variants see in this bird, which would also have provided a good indication of the sequence divergence betw
these two sub-species. Finally, the realignment of the short-read Illumina sequences, provides a way to estimate the num
sequence errors still present in the final assembly (seen as homozygous SNPs and indels).
Response 2：
We apologize, it is apparent that our description of the Tianfu goose used for genome assembly in this study was not clear
Domesticated geese derive from the swan goose (Anser cygnoides) and the graylag goose (Anser anser). The Tianfu goos
recognized breed that has originated from crosses between the domestic Landes goose (A. cygnoides) and the Sichuan wh
(A. anser), rather than the F1 animal crossed between A. anser and A. cygnoides. The Tianfu goose is a developed breed v
many outstanding characteristics, such as excellent egg-laying performance, a fast growth rate, and strong adaptability. T
characteristics are why we selected the Tianfu goose for this study.
Until now, a high-quality reference goose genome has not been available. To provide a valuable resource for future geneti
genomic studies on geese, and facilitate related research fields, our manuscript presents the first chromosome-level asser
the goose genome. With reference to human and mouse research, in future studies we also aim to perform haplotype-reso
genome assemblies of F1 geese and parent animals, and compare differences between breeds.
Regarding the estimation of sequence errors, after we obtained our final goose assembly, we realigned the short read Illur
sequences with BWA software, and called SNPs and InDels using GATK software. As can be seen in Table 3, the proportion
homologous SNPs and InDels identified (which often reflect assembly errors) were extremely low, which indicates that our

assembly is of “high-quality”.

Table 3: Homologous SNPs and InDels in the goose genome.

Category	Number	Proportion (%)
Homologous SNPs	23,324	0.0021
Homologous InDels	8,726	0.00078

OTHER POINTS

Comment 2-1:
Figure 1 and figure 2 are not very informative and I suggest moving these to the supplementary information
Response 2-1:
We agree with this suggestion from Reviewer 2. We have removed Figure 1 and Figure 4 to the supplementary figures, an[illegible] reordered the sequence of the corresponding supplementary figures.

Comment 2-2:
Line 89-90: The authors refer to table S1 in relation to the correction of sequencing errors. However, this table does not p[illegible] any information about sequencing errors.
Response 2-2:
We apologize for this inaccurate description. We have revised the main text to address this error, see lines 87–88.

Comment 2-3:
Line 90-91: The authors refer to table S2 and Fig S1. However, table S2 shows a summary of the pseudo chromosomes, [illegible] Hi-C scaffolds. Furthermore, in table S1 the authors show that there are 2123 Hi-C scaffolds. Please elaborate and clarify.
Response 2-3:
We regret the error in this description. Indeed, we state the length of the pseudo-chromosomes in the goose genome in T[illegible] and present the Hi-C interaction contact heatmap of the pseudo-chromosomes in Figure S3. There are 2123 scaffolds in o[illegible] genome assembly. This includes 68 scaffolds of 200bp to 2000bp, 2016 scaffolds of 2000bp to 350000bp, and 39 pseudo-chromosomes that are greater than 1Mb.

Comment 2-4:
Line 119-121: Again, the reference to the table/figure does not seem to match very well with the information in the text. [illegible] suggest to add the number of PCG's to table 3. Also, does figure 2 only show the TSS for PCG or does it also include those [illegible] lncRNAs.
Response 2-4:
Thank you for this valuable suggestion. Accordingly, we have added the number of PCGs to Table 3. In Figure 2, we show [illegible] TSSs for PCGs. We have redrawn Figure 2.

Comment 2-5:
Line 128: I am confused by the comment that the current assembly has more scaffolds. Given that the assembly is impro[illegible] higher N50 values for the contigs and scaffolds, I would assume that the number would be smaller.
Response 2-5:
Thank you for raising this point. To display the quality of the genome assemblies, we analyzed the distribution of their sca[illegible] lengths. In our goose genome, with the exception of the 39 pseudo-chromosomes, lengths of scaffolds are distributed fro[illegible] 350kb (Table 4). This indicates that our assembly contains 39 pseudo-chromosomes (longer than 1Mb) and 2016 scaffold[illegible] lengths ranging from 2kb to 350kb). To supply more information for researchers, we did not filter the 2kb–350kb scaffold[illegible] genome assembly data. As a result, we have reported more scaffolds in this study than were reported in two previous stu[illegible] However, as the 39 pseudo-chromosomes we assembled account for 88.36% of the genome (Table 4, Figure 2), (The tab[illegible] figures were accessible from Response_sup.pdf at: https://fnca1-my.sharepoint.com/:b:/g/personal/guanglianggaocq_lwh_world/EQSVOZIBkJ9HlaxEnZFufdcBf_eOaki7d4jJkNUz9Yrwpw?e[illegible] this suggests that our genome assembly is an improvement on previous goose assemblies.

Comment 2-6:
Line 129-131: This statement is not supported by table 3. In fact, the other studies seem to have annotated more gene s[illegible] than the current assembly.
Response 2-6:
Thank you for this useful comment. In this study, we annotated more repeat regions (8.67%) (Table S3) and exon seque[illegible] regions (26,883,354bp, 2.41%) (Table 3) than in previous studies (Table 3). This suggests that we have generated an im[illegible] genome assembly and annotation. We have revised lines 132–133 of the manuscript to address this point.

Comment 2-7:
Line 195-196: "… indicating that disease resistance may help …..". I don't think this statement is supported by the results[illegible] tends to be mere story telling.

Response 2-7:

Thank you for identifying this issue. In lines 198–211, we have revised the original text as follows: "Some of these PSGs, [illegible] GCH1 (GTP-cyclohydrolase I), are associated with parkinsonism, dystonia, and phenylketonuria disease in humans [7, 8]. [illegible] play a role in adaptation to high-altitude environments in humans, where they relate to a lower hemoglobin level, nitric ox[illegible] concentration, and oxygen saturation in the blood. Furthermore, previous studies have shown GCH1 divergence between [illegible] populations living at different altitudes [9]. Selection acting on GCH1 in goose is likely to be related to their adaption to hi[illegible] altitude or migratory habitats. SNW1 (SNW1 Domain Containing 1) is involved in the Nuclear Factor Kappa B pathway and [illegible] associated with oculopharyngeal muscular dystrophy disease [10, 11]. The depletion of this gene in breast cells leads to th[illegible] induction of apoptosis, while the overexpression of this gene impedes neural crest development [12]. Selection acting on [illegible] goose suggests that it may confer protection from diseases and aid adaptation in changeable environments. POU2F3 is piv[illegible] the discrimination of taste qualities, such as sweet, umami and bitter characteristics. Deficiency in this gene in mice alters [illegible] electrophysiology and behavioral responses to taste characters [13, 14]. Selection acting on POU2F3 in goose is likely to b[illegible] to a requirement for seeking food in variable migratory habitats."

References

1. Burton JN, Adey A, Patwardhan RP, Qiu R, Kitzman JO, Shendure J. Chromosome-scale scaffolding of de novo genome [illegible] assemblies based on chromatin interactions. Nat Biotechnol. 2013; 31(12): 1119-1125.

2. Ghurye J, Rhie A, Walenz BP, et al. Integrating Hi-C links with assembly graphs for chromosome-scale assembly. PLoS [illegible] Biol. 2019; 15(8): e1007273.

3. Liu B, Shi Y, Yuan J, et al. Estimation of genomic characteristics by analyzing k-mer frequency in de novo genome proje[illegible] 2013.

4. Wang G, Jin L, Li Y, et al. Transcriptomic analysis between Normal and high-intake feeding geese provides insight into a[illegible] deposition and susceptibility to fatty liver in migratory birds. BMC Genomics. 2019; 20(1): 372.

5. Lu L, Chen Y, Wang Z, et al. The goose genome sequence leads to insights into the evolution of waterfowl and suscepti[illegible] fatty liver. Genome Biol. 2015; 16(1): 89.

6. Gao G, Zhao X, Li Q, et al. Genome and metagenome analyses reveal adaptive evolution of the host and interaction wit[illegible] microbiota in the goose. Sci Rep. 2016; 6: 32961.

7. Yoshino H, Nishioka K, Li Y, et al. GCH1 mutations in dopa-responsive dystonia and Parkinson's disease. J Neurol. 2018[illegible] 1860-1870.

8. Gu Y, Lu K, Yang G, et al. Mutation spectrum of six genes in Chinese phenylketonuria patients obtained through next-g[illegible] sequencing. PLoS One. 2014; 9(4): e94100.

9. Guo YB, He YX, Cui CY, et al. GCH1 plays a role in the high-altitude adaptation of Tibetans. Zool Res. 2017; 38(3): 155[illegible]

10. Verma S, De Jesus P, Chanda SK, Verma IM. SNW1, a Novel Transcriptional Regulator of the NF-κB Pathway. Mol Cell [illegible] 2019; 39(3): e00415-18.

11. Tolde O, Folk P. Stress-induced expression of p53 target genes is insensitive to SNW1/SKIP downregulation. Cell Mol [illegible] 2011; 16(3): 373-384.

12. Wu MY, Ramel MC, Howell M, Hill CS. SNW1 is a critical regulator of spatial BMP activity, neural plate border formatior[illegible] neural crest specification in vertebrate embryos. PLoS Biol. 2011; 9(2): e1000593.

13. Huang YH, Klingbeil O, He XY, et al. POU2F3 is a master regulator of a tuft cell-like variant of small cell lung cancer. G[illegible] Dev. 2018; 32(13-14): 915-928.

14. Matsumoto I, Ohmoto M, Narukawa M, Yoshihara Y, Abe K. Skn-1a (Pou2f3) specifies taste receptor cell lineage. Nat N[illegible] 2011; 14(6): 685-687.

Close
