# Supplementary material for: Pacific Biosciences assembly with Hi-C mapping generates an improved, chromosome-level goose genome
Source: Gigascience. 2020 Oct 24;9(10):giaa114. doi: 10.1093/gigascience/giaa114 (PMC7585555; doi:10.1093/gigascience/giaa114)
Supplement: giaa114_Response_to_Reviewer_Comments_Revision_1 [file giaa114_response_to_reviewer_comments_revision_1.pdf]

# Author's Response To Reviewer Comments

Close

GigaScience
em@editorialmanager.com

Dear Hans Zauner
Please find enclosed our revised manuscript, "PacBio assembly with Hi-C mapping generates an improved, chromosome-level goose genome (GIGA-D-20-00133)", which we would like to resubmit to GigaScience.
We sincerely appreciate the very thoughtful and constructive comments from the editor(s). We have gone in detail through all the comments and believe that we have adequately addressed all their questions and concerns. We have made all the changes in the revised version of the manuscript, and our point-by-point responses to the editor's comments are given below. We trust that the revised manuscript now meets the standards required for publication in GigaScience.
We look forward to hearing a positive response from you.
Best regards
Mingzhou Li
Ph.D/Professor/
Address: Institute of Animal Genetics and Breeding, College of Animal Science and Technology, Sichuan Agricultural University, Chengdu 611130, China.
E-mail: mingzhou.li@sicau.edu.cn

Detailed responses to Editor(s)
Below, our responses are in black, all revisions in the manuscript are marked in red using the word's track change.

___________________________________________

Comment 1: Please include a citation to your new GigaDB dataset (including the DOI link) to your reference list, and cite this in the data availability section and elsewhere in the manuscript, where appropriate. The citation is: [xx] Li Y, Gao G, Lin Y, Hu S, Luo Y, Wang G et al. Supporting data for "PacBio assembly with Hi-C mapping generates an improved, chromosome-level goose genome" GigaScience Database. 2020. http://dx.doi.org/10.5524/100789. In the data availability section, please write something along the lines, "Supporting data, including [data type 1], [data type 2] [etc] is available via the GigaScience repository, GigaDB [xx]".
Response 1: In the data availability section (line 248 to 250), we added “The chromosome-level goose genome assembly, annotation files, and other supporting data are available via the GigaScience GigaDB database”, and we cited the new GigaDB dataset in line 418 to 420.

Comment 2: Do you have a picture of a representative of the goose hybrid used in your study (that can be published under a CC-BY open licence)? If you have a picture, please include this as Fig. 1, usually our "genome data note " authors show a representative of the organism/breed for illustration.
Response 2: As the suggestion from editor (s), we supplied a new picture to represent the Tianfu goose as the Figure1, and reordered the sequence of the corresponding supplementary figures.

Comment 3: Please also add the NCBI taxon ID for the species in the methods section. https://www.ncbi.nlm.nih.gov/taxonomy (If this particular hybrid does not have its own ID, please mention the NCBI taxon IDs of A. anser and A. cygnoides).
Response 3: Tianfu goose is a Chinese local breed with many outstanding characteristics, such as excellent egg-laying performance, a fast growth rate, and strong adaptability. The goose belonging to Anser cygnoides domesticus (NCBI: txid381198). In line 74, we added the NCBI taxon ID (NCBI: txid381198).

Comment 4: For bioinformatics software tools you used, please add RRIDs (Reserach Resource Identifiers) in the methods section for unique identification. You can find the RRIDs here: https://scicrunch.org/resources. For example, when you first mention BUSCO in the methods section, add the following RRID in this format: (BUSCO, RRID:SCR_015008).
Response 4: In the manuscript and supplemental material files, we supplied RRIDs for most of software and marked the changes in red. However, we did not found the RRIDs for the TACO software (line 130 of the text) and the PSYCHIC software (line 86 of the supplemental material) using the website (https://www.ncbi.nlm.nih.gov/taxonomy) or google search engine.

Moreover, we also made changes elsewhere in the text.
1. In the List of abbreviations section, we revised the “Anser anser: A. anser” to “A. anser: Anser anser” in line 252. In line 253, we revised “Anser cygnoides: A. cygnoides” to “A. cygnoides: Anser cygnoides”.
2. In line 285, we deleted the funding National Natural Science Foundation of China (31872335).

Close
