# Supplementary material for: Pacific Biosciences assembly with Hi-C mapping generates an improved, chromosome-level goose genome
Source: Gigascience. 2020 Oct 24;9(10):giaa114. doi: 10.1093/gigascience/giaa114 (PMC7585555; doi:10.1093/gigascience/giaa114)
Supplement: giaa114_Reviewer_1_Report_Original_Submission — David Burt -- 6/2/2020 Reviewed [file giaa114_reviewer_1_report_original_submission.pdf]

Reviewer Report

Title: PacBio assembly with Hi-C mapping generates an improved, chromosome-level goose genome

Version: Original Submission **Date:** 6/2/2020

Reviewer name: David Burt

Reviewer Comments to Author:

This paper reports on the assembly and annotation of the Tianfu goose genome, a female hybrid of A. anser x A. cygnoides. This assembly is a significant improvement on earlier genomes, which were based on short read technologies. This assembly is a hybrid of three technologies: short reads, long reads and HiC maps.

MAJOR POINTS

1. The assignment of 39 chromosomes to Hi-C scaffolds is very tentative and needs to be validated. For larger scaffold you could establish homology e.g. with chicken chromosomes, which have extensive FISH/cytogenetic data at least for the macrochromosomes. The smaller scaffolds in the HiC analysis could be parts of larger chromosomes - the HiC map suggests some mis-joins. Also in other genome projects very GC-rich, repeat-rich chromosomes (such as microchromosomes) are difficult if not impossible to sequence, and are missing from the assembly. So 39 pseudo-chromosomes are found but these do not equate to the 39 physical chromosomes. This affects conclusions on chromosome number, genome completeness, gene density distribution, distribution of TADs, etc. As a reference goose genome these points need to be addressed.

OTHER POINTS

1. This is a chromosome-level assembly make this clear in the text.
2. The hybrid approach used here is good but this is a rapidly evolving field, and is already superseded by technology (Pacbio HiFi now so polishing using short reads not needed) and software (e.g. Lachesis no longer supported).
3. The phrase "high-quality" is used throughout the text but not defined - so please define. It is more likely that sequence data is generated (provide QC data on quality) and then software is used to filter out poor data, to leave high-quality data for assembly.
4. For all software, please provide versions and source.
5. LINE 84: k-mer distribution analysis used to estimate genome size - provide reference, software, method - also mention other QC estimates (repeats, polyploidy etc).
6. LINE 91: Lachesis old software no longer supported - why not used SALSA2 or 3D-DNA?
7. Figure S1: Hi-C map suggests lots of mis-joins, have you checked and manually corrected?
8. LINES 109-111, again used the term "high-quality" for a mix of genomes, Human, Mouse, Chicken probably but duck, turkey and zebra finch are draft and not high-quality genomes.
9. LINES 114-117, pooled RNAseq used, so how can you quantify gene expression later in paper? Needs deconvolution of pooled samples - was this done? For annotation Pacbio isoseq would be better.
10. Prediction of lncRNAs from assembly of short read RNA-seq is known to be poor, so LINEs 121-124, where 3,287 lncRNAs are predicted needs to be taken with caution.

11. LINE 160, goose and duck diverged 32 Mya, how does this estimate compare with other data sources?
12. sections (b-d) interesting predictions from phylogenetic analyses, but all speculation, there is no other data provided to back up these predictions.
13. LINE 192, PAML Codeml analysis is crude, and does not correct for multiple testing, with 17K genes tested there is a high false positive rate, was there any correction for multiple testing, if not please correct.
14. LINE 202, the TAD analysis is restricted to liver tissue.
15. LINES 203-204, macs and mics form sub-domains in the nucleus. Figure 4 needs more explanation, poor figure.
16. LINE 205, define compartments A and B, how are these defined in Hi-C data?
17. LINE 206, how were TSS (transcription start sites, not defined in the set of abbreviations, please add) defined? I assume based on the pooled short read RNA-seq data. If correct, this is a poor data set, since the assembly of transcripts based on short read data only defines the most 5' RNA sequenced. So misses any internal TSS, does not correct for degraded RNA, etc.
18. LINE 213, gene expression levels based on pooled RNAseq data is a very poor dataset, should deconvolute or at least have a high-quality liver RNA set.
11.

Level of Interest

Please indicate how interesting you found the manuscript: Choose an item.

Quality of Written English

Please indicate the quality of language in the manuscript: Choose an item.

Declaration of Competing Interests

Please complete a declaration of competing interests, considering the following questions:

- Have you in the past five years received reimbursements, fees, funding, or salary from an organisation that may in any way gain or lose financially from the publication of this manuscript, either now or in the future?
- Do you hold any stocks or shares in an organisation that may in any way gain or lose financially from the publication of this manuscript, either now or in the future?
- Do you hold or are you currently applying for any patents relating to the content of the manuscript?
- Have you received reimbursements, fees, funding, or salary from an organization that holds or has applied for patents relating to the content of the manuscript?
- Do you have any other financial competing interests?
- Do you have any non-financial competing interests in relation to this paper?

If you can answer no to all of the above, write 'I declare that I have no competing interests' below. If your reply is yes to any, please give details below.

'I declare that I have no competing interests.

I agree to the open peer review policy of the journal. I understand that my name will be included on my report to the authors and, if the manuscript is accepted for publication, my named report including any attachments I upload will be posted on the website along with the authors' responses. I agree for my report to be made available under an Open Access Creative Commons CC-BY license (http://creativecommons.org/licenses/by/4.0/). I understand that any comments which I do not wish to be included in my named report can be included as confidential comments to the editors, which will not be published.

Choose an item.

To further support our reviewers, we have joined with Publons, where you can gain additional credit to further highlight your hard work (see: https://publons.com/journal/530/gigascience). On publication of this paper, your review will be automatically added to Publons, you can then choose whether or not to claim your Publons credit. I understand this statement.

Yes Choose an item.
