# Supplementary material for: Pacific Biosciences assembly with Hi-C mapping generates an improved, chromosome-level goose genome
Source: Gigascience. 2020 Oct 24;9(10):giaa114. doi: 10.1093/gigascience/giaa114 (PMC7585555; doi:10.1093/gigascience/giaa114)
Supplement: giaa114_Reviewer_2_Report_Original_Submission — Martien Groenen -- 6/10/2020 Reviewed [file giaa114_reviewer_2_report_original_submission.pdf]

Reviewer Report

Title: PacBio assembly with Hi-C mapping generates an improved, chromosome-level goose genome

Version: Original Submission **Date:** 6/10/2020

Reviewer name: Martien Groenen

Reviewer Comments to Author:

The manuscript describes a highly contiguous genome assembly of the goose genome and provides a significant improvement of the assembly of this bird. The results are described very clearly, and the data has been made publicly available. The analyses done are rather straightforward, and much more could have done with the interesting data generated in this study, which to me seems a missed opportunity. The authors decide to sequence an F1 animal that is a cross between A. anser and A. cygnoides. I wonder why the authors did not use Illumina sequencing to sequence the genome of the two parents. This would have allowed the generation of two haplotype specific assemblies and the comparison between the genomes of these two different sub-species. Also, no indication is given for the number of variants see in this bird, which would also have provided a good indication of the sequence divergence between these two sub-species. Finally, the realignment of the short-read Illumina sequences, provides a way to estimate the number of sequence errors still present in the final assembly (seen as homozygous SNPs and indels).

Minor comments:

Figure 1 and figure 2 are not very informative and I suggest moving these to the supplementary information.

Line 89-90: The authors refer to table S1 in relation to the correction of sequencing errors. However, this table does not provide any information about sequencing errors.

Line 90-91: The authors refer to table S2 and Fig S1. However, table S2 shows a summary of the pseudo chromosomes, not of the Hi-C scaffolds. Furthermore, in table S1 the authors show that there are 2123 Hi-C scaffolds. Please elaborate and clarify.

Line 119-121: Again, the reference to the table/figure does not seem to match very well with the information in the text. I also suggest to add the number of PCG's to table 3. Also, does figure 2 only show the TSS for PCG or does it also include those for the lncRNAs.

Line 128: I am confused by the comment that the current assembly has more scaffolds. Given that the assembly is improved with higher N50 values for the contigs and scaffolds, I would assume that the number would be smaller.

Line 129-131: This statement is not supported by table 3. In fact, the other studies seem to have annotated more gene sequences than the current assembly.

Line 195-196: "… indicating that disease resistance may help …..". I don't think this statement is supported by the results and tends to be mere story telling.

Level of Interest

Please indicate how interesting you found the manuscript: Choose an item.

Quality of Written English

Please indicate the quality of language in the manuscript: Choose an item.

Declaration of Competing Interests

Please complete a declaration of competing interests, considering the following questions:

- Have you in the past five years received reimbursements, fees, funding, or salary from an organisation that may in any way gain or lose financially from the publication of this manuscript, either now or in the future?
- Do you hold any stocks or shares in an organisation that may in any way gain or lose financially from the publication of this manuscript, either now or in the future?
- Do you hold or are you currently applying for any patents relating to the content of the manuscript?
- Have you received reimbursements, fees, funding, or salary from an organization that holds or has applied for patents relating to the content of the manuscript?
- Do you have any other financial competing interests?
- Do you have any non-financial competing interests in relation to this paper?

If you can answer no to all of the above, write 'I declare that I have no competing interests' below. If your reply is yes to any, please give details below.

I declare that I have no competing interests

I agree to the open peer review policy of the journal. I understand that my name will be included on my report to the authors and, if the manuscript is accepted for publication, my named report including any attachments I upload will be posted on the website along with the authors' responses. I agree for my report to be made available under an Open Access Creative Commons CC-BY license (http://creativecommons.org/licenses/by/4.0/). I understand that any comments which I do not wish to be included in my named report can be included as confidential comments to the editors, which will not be published.

Choose an item.

To further support our reviewers, we have joined with Publons, where you can gain additional credit to further highlight your hard work (see: https://publons.com/journal/530/gigascience). On publication of this paper, your review will be automatically added to Publons, you can then choose whether or not to claim your Publons credit. I understand this statement.

Yes Choose an item.
